# Zwitterionic amidinates as effective ligands for platinum nanoparticle hydrogenation catalysts†
†Electronic supplementary information (ESI) available. See DOI: 10.1039/c6sc05551f



**DOI:** 10.1039/c6sc05551f

**Published:** 2017-02-01

**Authors:** L. M. Martínez-Prieto, I. Cano, A. Márquez, E. A. Baquero, S. Tricard, L. Cusinato, I. del Rosal, R. Poteau, Y. Coppel, K. Philippot, B. Chaudret, J. Cámpora, P. W. N. M. van Leeuwen

**Affiliations:** a LPCNO , Laboratoire de Physique et Chimie des Nano-Objets , UMR5215 INSA-CNRS-UPS , Institut des Sciences Appliquées , 135, Avenue de Rangueil , F-31077 Toulouse , France . Email: vanleeuw@insa-toulouse.fr ; Email: lmmartin@insa-toulouse.fr; b Instituto de Investigaciones Químicas , CSIC-Universidad de Sevilla , C/Américo Vespucio, 49 , 41092 Sevilla , Spain . Email: campora@iiq.csic.es; c CNRS , LCC (Laboratoire de Chimie de Coordination) , Université de Toulouse , UPS , INPT , 205 route de Narbonne, BP 44099 , F-31077-Toulouse Cedex 4 , France

## Abstract

Ligand control of metal nanoparticles (MNPs) is rapidly gaining importance as ligands can stabilize the MNPs and regulate their catalytic properties. Herein we report the first example of Pt NPs ligated by imidazolium-amidinate ligands that bind strongly through the amidinate anion to the platinum surface atoms. The binding was established by ^15^N NMR spectroscopy, a precedent for nitrogen ligands on MNPs, and XPS. Both monodentate and bidentate coordination modes were found. DFT showed a high bonding energy of up to –48 kcal mol^–1^ for bidentate bonding to two adjacent metal atoms, which decreased to –28 ± 4 kcal mol^–1^ for monodentate bonding in the absence of impediments by other ligands. While the surface is densely covered with ligands, both IR and ^13^C MAS NMR spectra proved the adsorption of CO on the surface and thus the availability of sites for catalysis. A particle size dependent Knight shift was observed in the ^13^C MAS NMR spectra for the atoms that coordinate to the surface, but for small particles, ∼1.2 nm, it almost vanished, as theory for MNPs predicts; this had not been experimentally verified before. The Pt NPs were found to be catalysts for the hydrogenation of ketones and a notable ligand effect was observed in the hydrogenation of electron-poor carbonyl groups. The catalytic activity is influenced by remote electron donor/acceptor groups introduced in the aryl-*N*-substituents of the amidinates; *p*-anisyl groups on the ligand gave catalysts several times faster the ligand containing *p*-chlorophenyl groups.

## Abstract 2

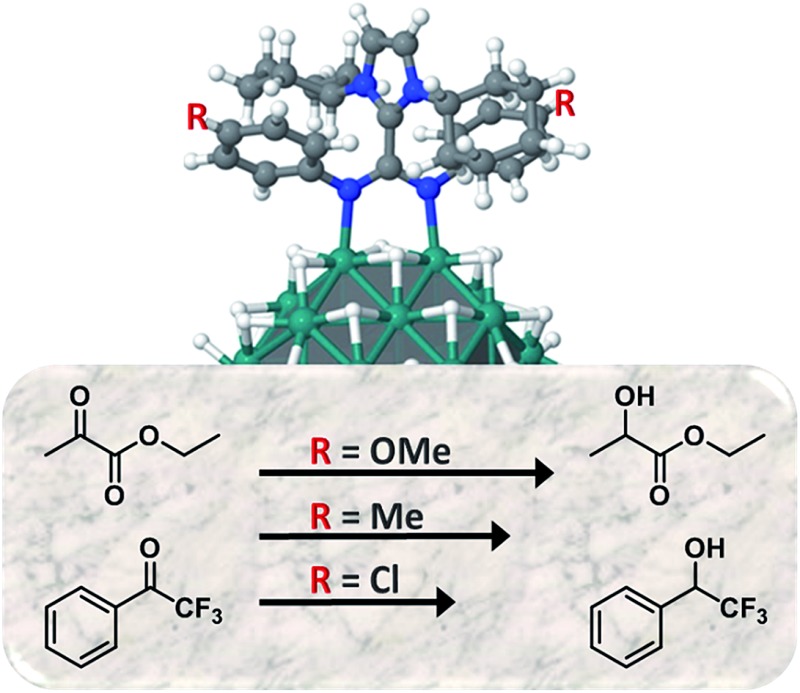
Pt NPs covered with zwitterionic amidinates as ligands exhibit an exciting ligand effect in the hydrogenation of carbonyl groups when electron donor/acceptor groups are introduced in the *N*-substituents.

## Introduction

The interest in metal nanoparticles (MNPs) is growing fast in both the academic and industrial community thanks to their applications in multiple fields such as sensors, medicine, optoelectronics and catalysis.^1^ In particular, MNPs have a high catalytic activity in some specific transformations like hydrogenation, polymerization, oxidation and C–C coupling reactions.^2^ Their high potential in catalysis arises from their particular electronic configuration and large surface area, and the potential to combine the advantages of homogeneous and heterogeneous catalysts. The stability and activity of MNPs are strongly influenced by the ligands used as stabilizers, which are able to modify their surface properties.^3^

**Scheme 1:**
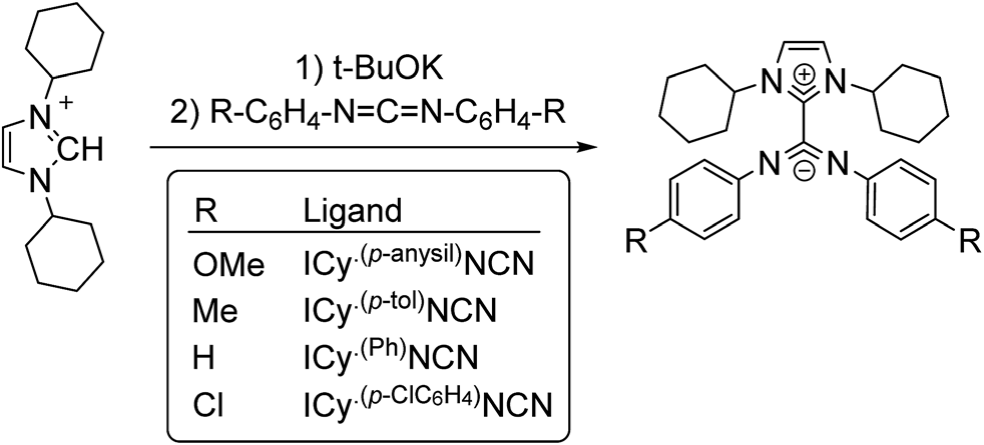
Synthesis of zwitterionic imidazolium-amidinate ligands.

The exploration of new families of ligands capable to stabilize MNPs and modify their reactivity is always a challenge. In a recent publication, the betaine-type adduct of N-heterocyclic carbenes (NHCs) and carbodiimides (Scheme 1), in particular 1,3-dicyclohexylimidazolium-2-di-*p*-tolylcarbodiimide (ICy·^(*p*-tol)^NCN), was identified as an effective ligand to produce ultra-small ruthenium nanoparticles (Ru NPs) with a size *ca.* 1 nm.^4^ Due to their zwitterionic structure, the nitrogen atoms present a large electron-donor capability and coordinate strongly to transition metals. The coordination chemistry of such imidazolium-amidinate ligands has been investigated a short while ago by some of us.^5^ In analogy with amidinates, which frequently coordinate in a μ^2^-κ^1^N, κ^1^N′ bridging mode, ICy·^(*p*-tol)^NCN forms multi-bridged binuclear “paddlewheel” complexes of copper(i). Besides, small changes on the *N*-substituents give us the possibility to modify their electronic properties. For example, introducing electron acceptor/donor moieties (–Cl or –OMe) in these pending groups enables us to modulate the surface properties of MNPs.

By the use of spectroscopic techniques, such as NMR or FT-IR, the coordination and dynamics of surface ligands of MNPs can be properly investigated. Recently, the coordination of NHC ligands to ruthenium and platinum nanoparticles was investigated by solid-state NMR spectroscopy.^6^ Moreover, NMR studies on Ru and Pt NPs stabilized by hexadecylamine (HDA) confirmed that Pt displays a Knight shift (due to the presence of free electrons on the surface) which is much stronger than that of Ru.^7^ This effect was also observed in Pt NPs of 1.2 and 2.0 nm, for which the different electronic states resulted in unequal magnitudes of Knight shift.^8^ However, ultra-small Pt NPs (<1 nm) behave as molecular species without free electrons and no Knight shift occurs.^9^ Another important instrument to characterize the surface of the nanoparticles is the use of carbon monoxide (CO) as a probe molecule, since CO coordination can be especially useful to determine the active sites.^8,10^

Theoretical calculations are of great importance in order to shed more light on experimental data, as for example to secure the assignment of experimental solid state NMR spectra or for the calculation of properties that cannot be measured experimentally.^11^ In the context of the nanoparticle surface chemistry, the theoretical calculations provide detailed information not only on the coordination mode of the species bound to the nanoparticle surface,^12^ but also on the chemical reactions that may take place on these surfaces.^13^

Ligand-stabilized metal NPs are effective catalysts for hydrogenation of olefin, carbonyl and nitro functionalities, as well as aromatics.^14^ In this context, Pt NPs have shown important chemoselectivities in hydrogenation of olefin^6b,15^ and nitro groups.^16^ Herein we present a series of Pt NPs ligated by imidazolium-amidinates (ICy·^(Ar)^NCN) which exhibit an exciting ligand effect in the hydrogenation of carbonyl groups when electron donor/acceptor groups are introduced in the *N*-substituents. These Pt NPs were characterized by the state-of-the-art techniques (TEM, HRTEM, WAXS, TGA and EA) and studied their surface chemistry through the coordination of CO (FT-IR and solid state MAS NMR). Here a clear correlation between the Knight shift of adsorbed CO and the NPs size was observed. In addition, the coordination of the imidazolium-amidinate ligands was investigated in Pt and Ru NPs by XPS, solid state ^15^N MAS NMR and DFT calculations.

## Results and discussion

### Synthesis and characterization of Pt NPs

Zwitterionic adducts of *N*,*N*-dicyclohexylimidazolidene and diarylcarbodiimide (ICy·^(Ar)^NCN; Ar = Ph, *p*-tol, *p*-anisyl, *p*-ClC_6_H_4_) were prepared similarly to previously reported betaine-type adducts,^4,5^ by reaction of N-heterocyclic carbene (ICy) and a suitable carbodiimide, namely bis(4-methylphenyl)methanediimine (^(*p*-tol)^NCN), bis(4-methoxyphenyl)methanediimine (^(*p*-anisyl)^NCN), bis(4-chlorophenyl)methanediimine (^(*p*-ClC_6_H_4_)^NCN) and ^15^N-labelled bis-phenylmethanediimine (^(Ph)^NC^15^N) (Scheme 1). Non-commercial carbodiimides were obtained in high yields by desulphurization of the corresponding thioureas with I_2_ in the presence of NEt_3_, using literature procedures.^17^ The symmetric thioureas (*p*-RC_6_H_4_)_2_C=S (R = OMe, Cl) were readily synthesized from thiophosgene and the corresponding aniline. ^15^N-labeled diphenylthiourea used to prepare ^(Ph)^NC^15^N was obtained from phenylisothiocyanate and aniline.^17*a*^ With this method we expected to form the singly ^15^N-labeled product, but the ESI-MS spectrum of the thiourea showed a signal for the corresponding anion of which the isotopic pattern was consistent with a statistical 1 : 2 : 1 mixture of the normal (^14^N_2_), monolabeled (^14^N^15^N) and doubly-labeled isotopologues (^15^N_2_). As shown in Scheme S1 (see ESI, Section S1†), scrambling of the ^15^N label indicates that aniline addition to phenylisothiocyanate is reversible.

**Scheme 2:**
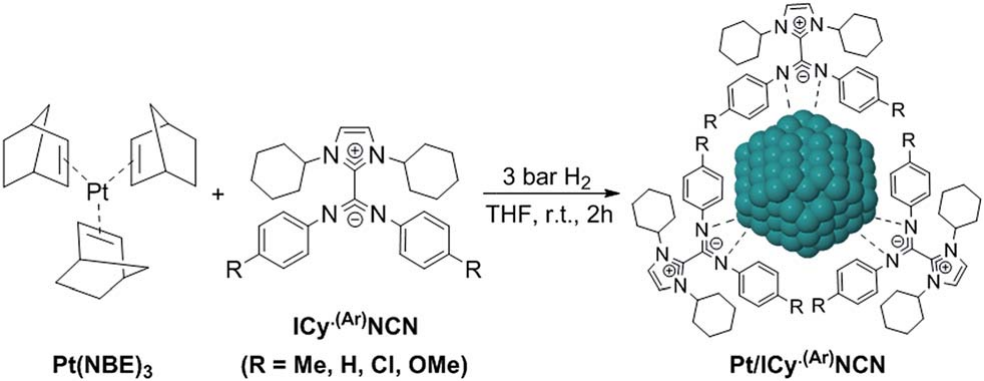
Synthesis of platinum nanoparticles using imidazolium-amidinate ligands as stabilizers.

**Fig. 1:**
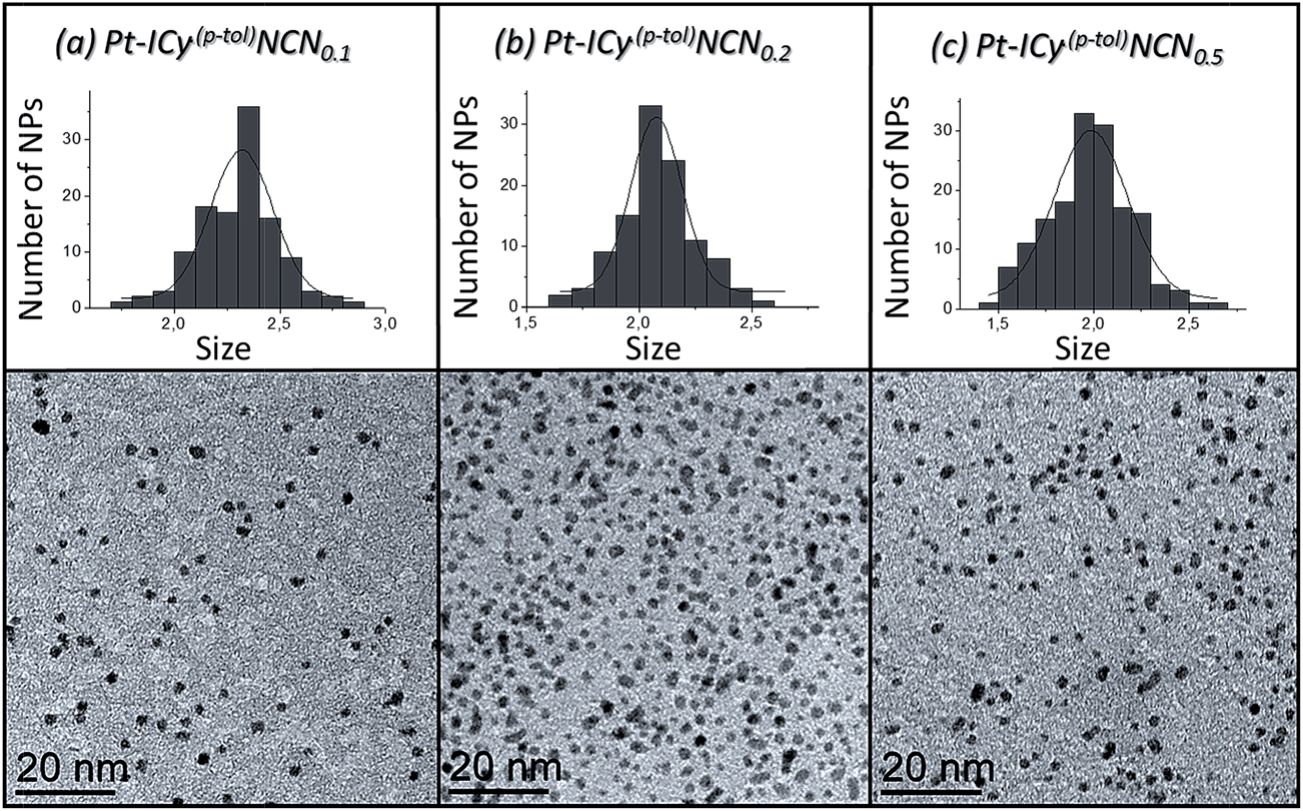
TEM images and size histograms of (a) Pt/ICy·^(*p*-tol)^NCN_0.1_, (b) Pt/ICy·^(*p*-tol)^NCN_0.2_ and (c) Pt/ICy·^(*p*-tol)^NCN_0.5_.

Most of Pt NPs reported in this paper were conveniently obtained by reaction of tris(2-norbornene)platinum(0) [Pt(NBE)_3_] in THF under 3 bar H_2_ in the presence of the corresponding imidazolium-amidinate ligand (Scheme 2). Herein we report for the first time the use of Pt(NBE)_3_ as metallic precursor in the synthesis of Pt NPs by the organometallic approach. The advantages of Pt(NBE)_3_ *versus* the typical Pt precursors [tris(dibenzylideneacetone)diplatinum (0); Pt_2_(DBA)_3_ or dimethyl(1,5-cyclooctadiene)platinum(ii); Pt(CH_3_)_2_(COD)] are evident.^7,8,18^ First, the purification of the obtained nanoparticles is simplified as the norbornane formed during the decomposition can be rapidly eliminated under vacuum and makes it this complex a “clean” precursor. And, second, the highly reactive Pt(NBE)_3_ requires less time for the formation of Pt NPs under H_2_. Thus, the use of Pt(NBE)_3_ as precursor is advantageous both for the formation and purification of Pt NPs. Different amounts of ICy·^(*p*-tol)^NCN were employed for the synthesis of Pt NPs, specifically 0.1, 0.2 and 0.5 molar equiv. (Pt/ICy·^(*p*-tol)^NCN_0.1_, Pt/ICy·^(*p*-tol)^NCN_0.2_ and Pt/ICy·^(*p*-tol)^NCN_0.5_), with the aim to obtain different sizes and reactivities. Indeed, Transmission Electronic Microscopy (TEM) micrographs of Pt/ICy·^(*p*-tol)^NCN_0.1_, Pt/ICy·^(*p*-tol)^NCN_0.2_ and Pt/ICy·^(*p*-tol)^NCN_0.5_ exhibit Pt NPs with a mean diameter of 2.3 (0.3), 2.1 (0.2) and 1.9 (0.4) nm respectively (Fig. 1), and one observes a correlation between the quantity of ligand and the average size of the obtained NPs; more ligand leads to smaller particles. This behavior was already observed in our previous work with Ru/ICy·^(*p*-tol)^NCN,^4^ and also with other type of ligands, such as NHCs,^6a,19^ aminosilanes^20^ and sulfonated diphosphines.^21^ Pt/ICy·^(*p*-tol)^NCN_0.1_ and Pt/ICy·^(*p*-tol)^NCN_0.2_ micrographs revealed NPs that are very well distributed and monodisperse in size, while the image for Pt/ICy·^(*p*-tol)^NCN_0.5_ showed a worse dispersion. For an unknown reason, the range of sizes of these NPs increases with the amount of ligand, while the monodispersity is lost.

**Fig. 2:**
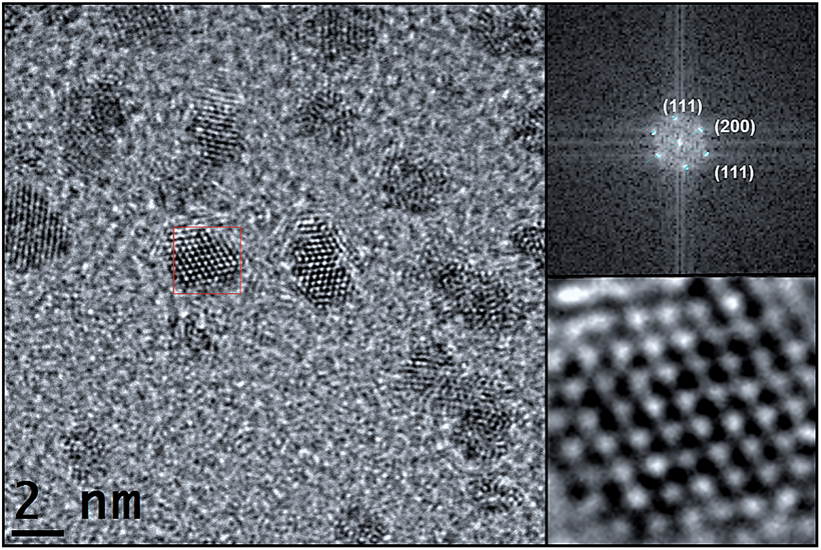
HRTEM image of Pt/ICy·^(*p*-tol)^NCN_0.2_ (left, right bottom) and fast Fourier transformation analysis (right, top) with the planar reflections.

High resolution TEM (HRTEM) micrographs of Pt/ICy·^(*p*-tol)^NCN_0.2_ show crystalline Pt NPs (Fig. 2), displaying the face centered cubic (fcc) structure typical of bulk Pt. Fast Fourier Transformation (FFT) applied to the image of Fig. 2 revealed the reflections corresponding to the (111), (200) and (111) atomic planes.

Wide-Angle X-ray Scattering (WAXS) analyses performed on solid samples of Pt/ICy·^(*p*-tol)^NCN_0.1_, Pt/ICy·^(*p*-tol)^NCN_0.2_ and Pt/ICy·^(*p*-tol)^NCN_0.5_ confirmed the crystallinity of these Pt NPs, which retain the fcc structure (see ESI, Fig. S17–S19†). The coherence lengths indicated by the Radial Distribution Function (RDF) resultant from WAXS analysis are slightly higher than the mean diameters determined by TEM. WAXS analysis revealed a coherence length of 2.5 nm for Pt/ICy·^(*p*-tol)^NCN_0.1_, 2.2 nm for Pt/ICy·^(*p*-tol)^NCN_0.2_, and 2.1 nm for Pt/ICy·^(*p*-tol)^NCN_0.5_, while the mean diameters observed by TEM were 2.3, 2.1 and 1.9 nm, respectively.

The Pt NPs were obtained in acceptable yields (60–70%; based on Pt), and the metal content was determined by thermogravimetric analysis (TGA). The Pt content was 90.1% for Pt/ICy·^(*p*-tol)^NCN_0.1_, 77.3% for Pt/ICy·^(*p*-tol)^NCN_0.2_, and 60.1% for Pt/ICy·^(*p*-tol)^NCN_0.5_. Moreover, elemental analysis (EA) of the NPs revealed that the amounts of C, H and N are in good agreement with the ratio of these elements in the ligand (C, 79.25%; H, 8.42%; N, 12.32%), suggesting that the imidazolium-amidinate ligand remains intact at the platinum surface (see ESI, Table S1†). It is important to mention that the Pt : L ratio in Pt/ICy·^(*p*-tol)^NCN_0.5_ shows a large quantity of coordinated ligand at the platinum surface, as the number of ICy·^(*p*-tol)^NCN molecules is approximately half of the surface atoms (Pt_surface_ ∼ 50% of Pt_total_).

### Surface studies

**Fig. 3:**
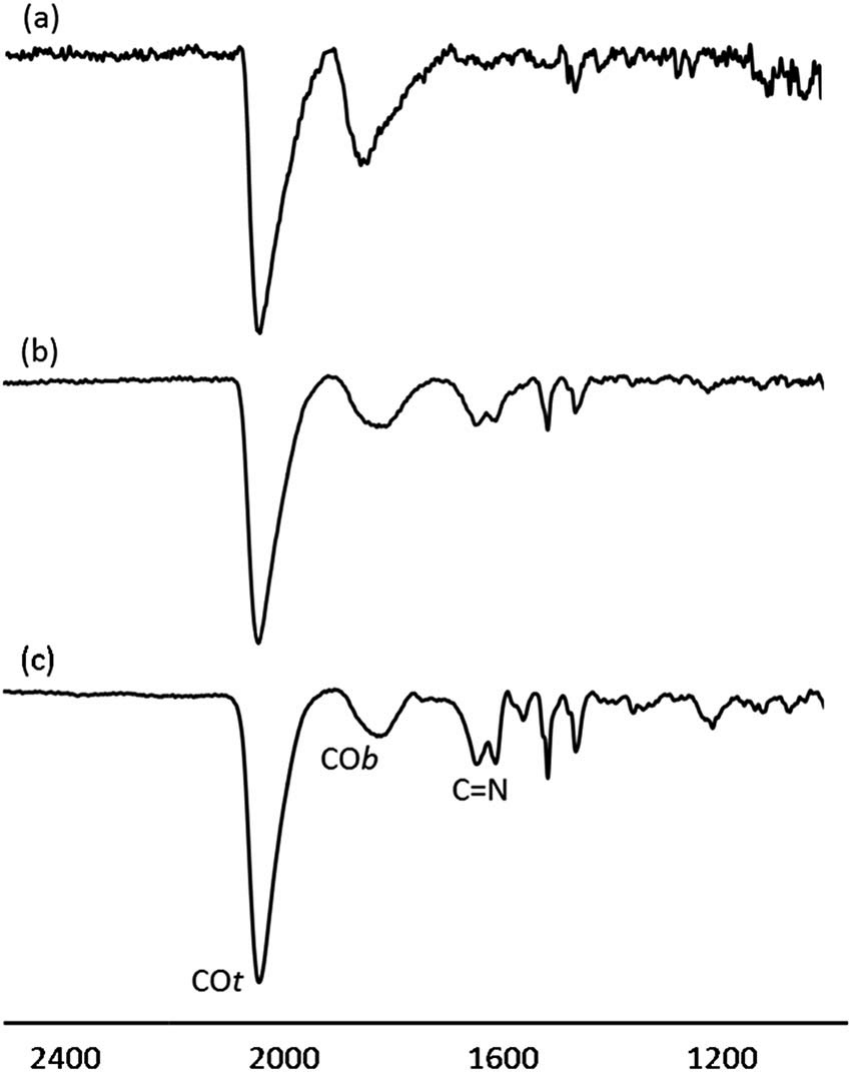
ATR FT-IR spectra of (a) Pt/ICy·^(*p*-tol)^NCN_0.1_, (b) Pt/ICy·^(*p*-tol)^NCN_0.2_ and (c) Pt/ICy·^(*p*-tol)^NCN_0.5_ after CO adsorption.

The surface chemistry of Pt/ICy·^(*p*-tol)^NCN NPs was studied by Fourier transform infrared (FT-IR) and magic angle spinning solid-state ^13^C and ^15^N NMR (MAS-NMR) with and without cross-polarization (CP). CO was used as a probe molecule as it is well known that coordination of CO allows the identification of different available surface sites on metal nanoparticles.^10,22^ Commonly, CO is adsorbed onto the metal surface either in a bridging (COb) or in a terminal (COt) mode. Fig. 3 shows attenuated total reflectance (ATR) FT-IR spectra of Pt/ICy·^(*p*-tol)^NCN NPs after bubbling CO during 5 min in a THF solution. The FT-IR spectrum of Pt/ICy·^(*p*-tol)^NCN_0.1_ [Fig. 3(a)] presents two distinct bands, at 1845 cm^–1^ and 2038 cm^–1^, which can be respectively attributed to CO coordinated on the Pt surface in a bridging (COb) and in a terminal mode (COt). The stretching band attributed to COb is very intense, evidencing that a large part of the CO is coordinated in a bridging mode. Analogous stretching vibrations for adsorbed CO were previously observed in Pt(111) crystal surfaces and Pt NPs.^23^ The IR spectrum of Pt/ICy·^(*p*-tol)^NCN_0.2_ displays similar frequencies at 1816 cm^–1^ and 2032 cm^–1^, but here the ratio COt/COb has increased [Fig. 3(b)] in comparison with the previous one. This trend persists for Pt/ICy·^(*p*-tol)^NCN_0.5_, and the intensities of CO bands (1808 and 2032 cm^–1^) decrease in relation to the characteristic C=N absorption frequencies of the amidinate ligand at 1630 and 1598 cm^–1^ [Fig. 3(c)] Here we observe an evident relation between the COt/COb ratio and the amount of ligand used. We think that both the size and the presence of more ligand on the surface increase the amount of COt at the cost of COb, such that 1.9 nm Pt/ICy·^(*p*-tol)^NCN_0.5_ NPs contains mostly COt. It is noteworthy that when we decrease the amount of ICy·^(*p*-tol)^NCN from 0.5 to 0.1 equiv., we observe a slight shift of the CO bands to higher frequency (from 1808 to 1845 cm^–1^ for COb and from 2032 to 2038 cm^–1^ for COt). This behavior is in agreement with a higher surface coverage of CO in Pt/ICy·^(*p*-tol)^NCN_0.1_, which removes more electron density from the Pt surface (see ESI, Fig. S25†). The displacement of the CO stretching frequency in relation with the quantity of CO has been previously described for surfaces and MNPs.^8,23b,24^

Thanks to FT-IR we obtained the first indication about the coordination mode of the imidazolium-amidinate ligand. Comparing the IR spectra of the ligand ICy·^(*p*-tol)^NCN (see ESI, Fig. S20†) and the particle Pt/ICy·^(*p*-tol)^NCN_0.5_ (see ESI, Fig. S26†), we observed a clear 100 cm^–1^ displacement of the strong stretching C=N bands, from 1530 and 1495 cm^–1^ to 1630 and 1598 cm^–1^, which suggests that the coordination of ICy·^(*p*-tol)^NCN takes place through the NCN moiety. These observed frequencies are in agreement with reported values.^5,25^

**Fig. 4:**
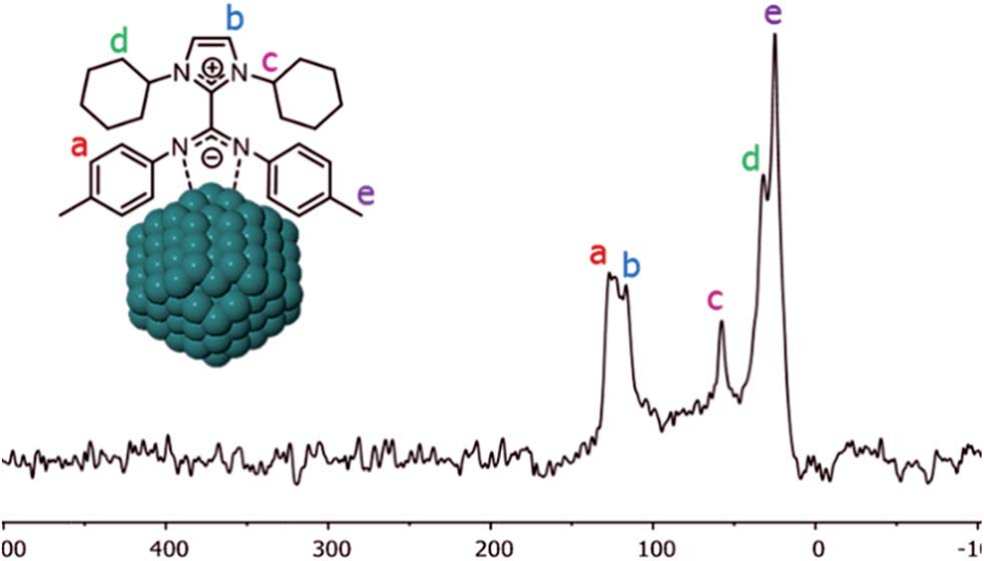
^13^C CP-MAS NMR spectrum of Pt/ICy·^(*p*-tol)^NCN_0.5_ NPs.

Coordination of CO on the Pt NPs was also studied by solid-state NMR. The ^13^C CP-MAS NMR spectrum obtained for Pt/ICy·^(*p*-tol)^NCN_0.5_ before exposition to ^13^CO displays most of the characteristic signals of the ICy·^(*p*-tol)^NCN ligand (Fig. 4). The group of peaks at 125 ppm (a) corresponds to the aromatic ring of the *p*-tolyl moiety and the signal at 116 ppm (b) belongs to the aromatic imidazolium backbone. The distinctive peak at 58 ppm (c) is attributed to the methine group next to the nitrogen atom. The cyclohexyl methylene resonances are observed between 32 ppm and 25 ppm (d) overlapping with the *p*-tolyl methyl group resonance at 25 ppm (e). As was previously observed for Ru/ICy·^(*p*-tol)^NCN, the signals for the C_*ipso*_ of the *p*-tolyl group and the imidazolium and carbodiimide quaternary NCN atoms are not clearly observable in the ^13^C spectrum (usually in the 130–150 ppm range; see ESI, Fig. S33†) due to a line broadening caused by the coordination of ICy·^(*p*-tol)^NCN at the metal surface.^4^

**Fig. 5:**
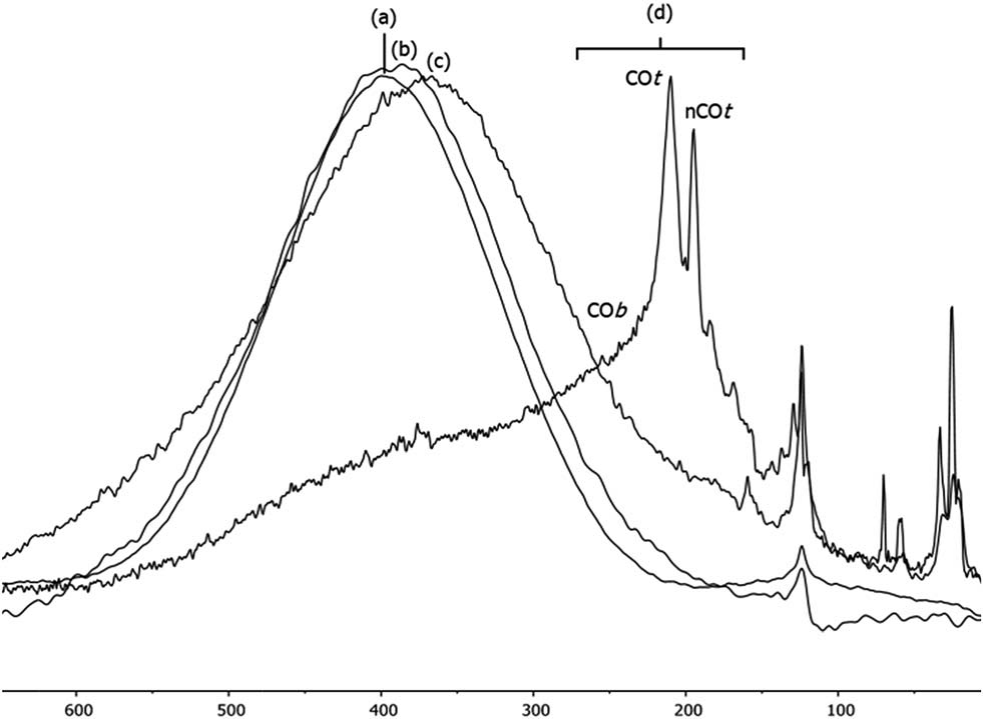
^13^C MAS NMR spectrum of (a) Pt/ICy·^(*p*-tol)^NCN_0.1_ (2.3 nm), (b) Pt/ICy·^(*p*-tol)^NCN_0.2_ (2.1 nm), (c) Pt/ICy·^(*p*-tol)^NCN_0.5_ (1.9 nm) and (d) Pt/CO/ICy·^(*p*-tol)^NCN_0.2_ (1.2 nm) after exposure to ^13^CO (1 bar, 20 h, at r.t.).

**Scheme 3:**
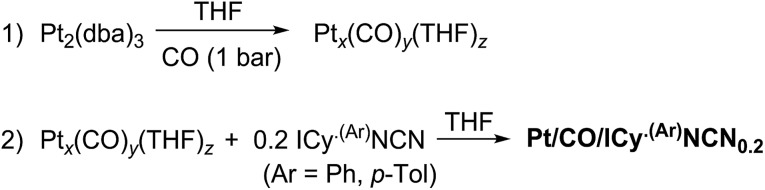
Synthesis of Pt/CO/ICy·^(*p*-tol)^NCN_0.2_ NPs.

When the Pt/ICy·^(*p*-tol)^NCN_0.5_ NPs (1.9 nm) were exposed to 1 bar of ^13^CO during 20 h at r.t., the ^13^C MAS NMR spectrum [Fig. 5(c)] showed a very broad peak centered at *ca.* 360 ppm which corresponds to coordinated CO. This broad and high frequency resonance can be assigned to a Knight-shifted ^13^CO signal. In the ^13^C MAS spectra of Pt/ICy·^(*p*-tol)^NCN_0.2_ (2.1 nm) and Pt/ICy·^(*p*-tol)^NCN_0.1_ (2.3 nm) after reaction with ^13^CO we also observed the broad peak of CO at 390 and 400 ppm respectively [Fig. 5(a) and (b)]. Thus, when the size of the NPs decreases, the CO signal is displaced to lower frequency. Because of the fast relaxation associated with the presence of surface electrons (paramagnetic effect), the efficiency of the magnetization transfer in the CP experiments is dramatically reduced and no signal for the coordinated CO could be observed in the ^13^C CP-MAS spectra (see ESI, bottom of Fig. S28, S30 and S32†). As we observed an evident relationship between the size and the magnitude of the Knight shift on the CO band, we decided to synthesize very small Pt/ICy·^(*p*-tol)^NCN NPs with the intention to suppress the Knight shift completely. Pt NPs of *ca.* 1.2 (0.3) nm (see ESI, Fig. S8†) were prepared using a well-known two-step procedure.^7,23a^ Pt_2_(DBA)_3_ was decomposed under 1 bar of CO in THF, forming the colloid Pt_*x*_(CO)_*y*_(THF)_*z*_, and after washing this colloid with pentane, 0.2 equiv. of ICy·^(*p*-tol)^NCN were added to obtain Pt/CO-ICy·^(*p*-tol)^NCN_0.2_ NPs (Scheme 3). The ^13^C MAS spectrum of Pt/CO/ICy·^(*p*-tol)^NCN_0.2_ [Fig. 5(d)] after reaction with ^13^CO showed a broad peak at 230 ppm due to bridging CO (COb) and two sharp peaks at 210 ppm and 194 ppm which correspond to terminal (COt) and multi-terminal CO (nCOt), respectively. These ones are the values expected for bridging and terminal CO in Pt NPs.^8,9,18b^ However, we still observe a remaining broad Knight-shifted CO resonance at 350 ppm due to the CO coordinated on the larger NPs. ^13^C Knight-shifted resonances have already been observed in Pt NPs,^7–9,26^ but this is the first case which reports a direct dependence of the size of Pt NPs with the degree of the Knight shift observed, perceiving a displacement of the CO band as the nanoparticle size decreases (2.3 nm–2.1 nm–1.9 nm), and at 1.2 nm the Knight shift is almost suppressed (Fig. 5).

To understand the state and coordination mode of these amidinate ligands, we used the labelled adduct 1,3-dicyclohexylimidazolium-2-di-phenylcarbodiimide (ICy·^(Ph)^NC^15^N) and studied its coordination on the NP metal surface by ^15^N MAS NMR spectroscopy. ICy·^(Ph)^NC^15^N was fully characterized by NMR (liquid and solid state) and DFT calculations (see ESI,† Section S1). Employing the aforementioned synthetic method, Pt/ICy·^(Ph)^NC^15^N_0.5_ NPs with an average size of 1.9 (0.2) nm (see ESI, Fig. S9†) were prepared (Scheme 1). These NPs were studied by ^15^N MAS NMR but we could not observe any detectable signal in ^15^N Hahn-echo NMR [see ESI, Fig. S36(a)†] not even in ^15^N CP/Hahn-echo MAS NMR [see ESI, Fig. S36(b)†], which are usually employed to detect broad NMR signals. The lack of signals is possibly due to the Knight shift as discussed above.

**Fig. 6:**
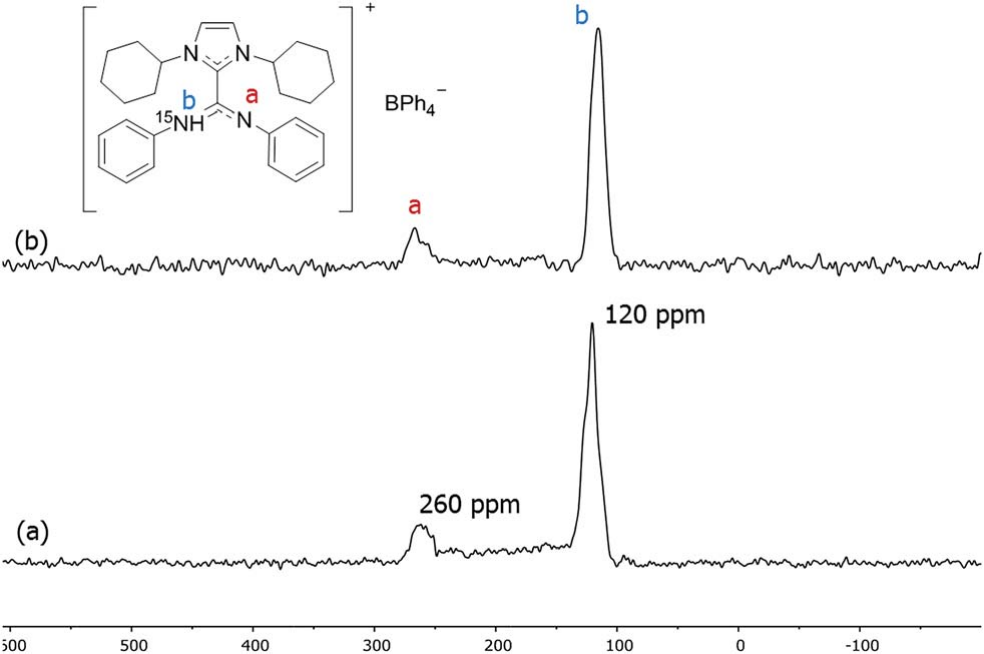
^15^N CP/Hahn-echo MAS of (a) Pt/CO/ICy·^(Ph)^NC^15^N_0.2_ and (b) [H·ICy·^(Ph)^NC^15^N]^+^·[BPh_4_]^–^.

To suppress the Knight shift we synthesized Pt NPs of *ca.* 1.2 nm with labelled ICy·^(Ph)^NC^15^N, Pt/CO/ICy·^(Ph)^NC^15^N_0.2_ (see ESI, Fig. S10†). The ^15^N CP/Hahn-echo MAS NMR spectrum displays two sharp signals at 120 and 260 ppm [Fig. 6(a)]. The sharpness of the signals points to a non-coordinated nitrogen compound, as the signals of coordinated ligands are usually broad. The intense peak at 120 ppm is reminiscent of a protonated nitrogen ligand, therefore we prepared the singly-protonated phenyl betaine adduct as its tetraphenylborate salt [ICy·^(Ph)^NC^15^NH]^+^[BPh_4_]^–^. In contrast with the strongly basic ligand Me_2_IiPr·^(iPr)^NCN reported by Kuhn,^27^ aryl imidazolium-amidinate ICy·^(Ar)^NCN (*e.g.* Ar = Tol) does not react with water to a significant extent, but they are immediately protonated by acetic acid (HOAc). [ICy·^(Ph)^NC^15^NH]^+^[BPh_4_]^–^ was obtained reacting ICy·^(Ph)^NC^15^N with HOAc in the presence of NaBPh_4_. Indeed the solid-state ^15^N CP/Hahn-echo MAS spectrum of this compound exhibits two signals at 116 and 266 ppm, attributed to the protonated and non-protonated N atoms, respectively. The former is *ca.* four-fold as intense as the latter, because the CP effect is much more intense in the protonated nitrogen due to the presence of the strong N–H dipolar coupling [Fig. 6(b)]. Solution NMR spectra in CD_2_Cl_2_ (see ESI,† Section S1) indicate that the cation exists as a 2 : 1 mixture of two isomers obtained by protonation of either one of the two N-atoms. DFT calculations confirm the experimental shifts and corroborate that the two *E*/*Z* isomers of [ICy·^(Ph)^NC^15^NH]^+^[BPh_4_]^–^ can coexist as evidenced by their relative energy difference of 1.1 kcal mol^–1^. In the same way as for non-protonated ligands, the *E*/*Z* isomers are more stable than the *Z*/*Z* isomer by –3.4 kcal mol^–1^ and –2.3 kcal mol^–1^ (see ESI, Fig. S59†). Thus, the peaks at 120 and 260 ppm in the ^15^N CP/Hahn-echo MAS NMR spectrum of Pt/CO/ICy·^(Ph)^NC^15^N_0.2_ correspond to a minor part of the ligand that has been protonated during the synthesis and the signal of which is strongly enhanced thanks to the ^15^N CP-MAS experiment. However, we were not able to detect ^15^N resonances of coordinated ICy·^(Ph)^NC^15^N in ^15^N Hahn-echo MAS NMR, most likely because of the deleterious effect of the NP surface electrons (see ESI, Fig. S38†).

**Fig. 7:**
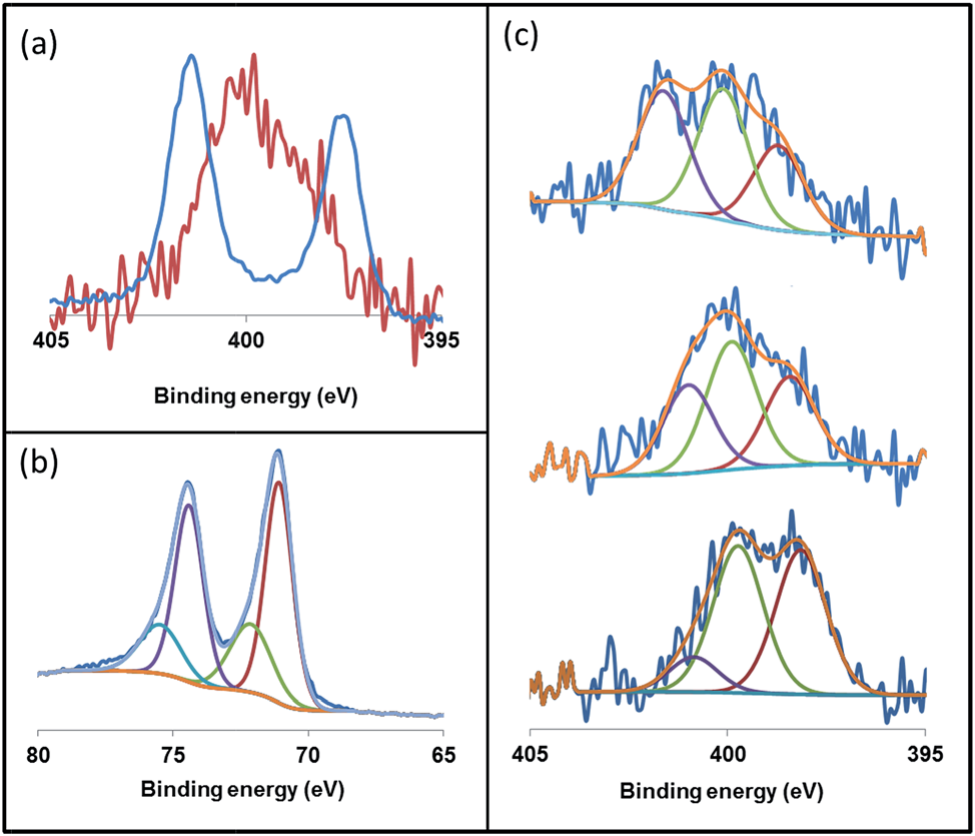
X-ray photoelectron spectroscopy (XPS) of (a) the N 1s signals of ICy·^(*p*-tol)^NCN (blue) and Pt/ICy·^(*p*-tol)^NCN_0.2_ (red), (b) the Pt 4f_5/2_ and 4f_7/2_ signals of Pt/ICy·^(*p*-tol)^NCN_0.2_ and (c) the N 1s signals of Pt/ICy·^(*p*-ClC_6_H_4_)^NCN_0.2_ (top), Pt/ICy·^(*p*-tol)^NCN_0.2_ (center) and Pt/ICy·^(*p*-anisyl)^NCN_0.2_ (bottom).

X-ray photoelectron spectroscopy (XPS) is an ideal technique to measure the elemental composition and chemical state of surface catalysts. Recently, this technique has been described as a useful tool to investigate the binding mode of NHC ligands on a metal surface.^28^ Thus, we applied XPS to study the coordination of the imidazolium-amidinate ligands in our Pt NPs. The N 1s signals of ICy·^(*p*-tol)^NCN present binding energies (BE) of 401.3 and 397.4 eV [Fig. 7(a), blue]. The highest BE peak at 401.3 eV can be assigned to the tightly bound electrons of the nitrogen atoms of the imidazolium fragment. The second peak at 397.4 eV derives from the N atoms of the amidinate group, which bear partial negative charges.^29^ The coordination of ICy·^(*p*-tol)^NCN through the latter N-atoms to the platinum surface involves a loss of electron density, which increases the binding energy (400.1 eV) compared to the N-atoms of free ICy·^(*p*-tol)^NCN (397.4 eV). This leads to the overlap of the peaks of the two fragments, as can be seen in Fig. 7(a) (red). This signal is the result of convolutions of peaks corresponding to imidazolium and amidinate N atoms, coordinated or not. In order to improve our understanding of these binding energies, we theoretically compared the natural charges of the N atoms of the ICy·^(Ph)^NCN ligand, free and coordinated in μ^2^-κ^1^N, κ^1^N′ mode to a model Ru carbonyl cluster (see ESI, Fig. S60†). Interestingly, no change is observed in the natural charges of the N atoms of the imidazolium fragment by coordination of the ligand on the cluster surface. However, the anionic character of the N atoms of the amidinate moiety is partially reduced, as a result of the balance between σ-donation and π-backdonation. Thus, the coordination of the ligand induces the decrease of the charge difference between the N atoms of both moieties leading to overlap of the peaks of the two fragments as observed in Fig. 7(a) (red). This result suggests that the coordination of the ligand to the nanoparticle is through the N atoms of the amidinate moiety. We deconvoluted the N 1s signals of Pt/ICy·^(*p*-tol)^NCN_0.2_ at 399.9 eV in three contributions with different binding energies, at 401.1, 399.7 and 398.2, corresponding to *δ* ^–^, neutral and *δ* ^+^ N atoms, respectively [Fig. 7(c), center]. When the electron-withdrawing ligand ICy·^(*p*-ClC_6_H_4_)^NCN is used as stabilizer, the contribution of the peak at 401.1 (N^*δ*+^) intensifies, generating an increase of the average BE of the N 1s signal to 400.8 eV [Fig. 7(c), top]. On the other hand, the N(1s) signals of Pt/ICy·^(*p*-anisyl)^NCN_0.2_ containing the electron donor OMe substituent, display the opposite behavior, intensifying the peak at 398.2 (N^*δ*–^) and decreasing the global BE to 398.7 eV [Fig. 7(c), bottom]. The binding energy of Pt 4f_7/2_ in the XPS spectra of Pt/ICy·^(*p*-tol)^NCN_0.2_ NPs is 71.1 eV. The main contribution is located at 71.0 eV, which is characteristic of Pt(0),^30^ and an additional contribution at 72.7 eV is attributed to the surface platinum atoms linked to the nitrogen atoms of ICy·^(*p*-tol)^NCN (Pt(*δ*+)) [Fig. 7(b)].^31^

### Coordination of imidazolium-amidinates to metal surfaces

Amidinates have a rich coordination chemistry^32^ as they can coordinate to a single metal center either in terminal (κ^1^), chelating (κ^2^) modes, and, less often they can even bind in η^3^ mode, like an allyl group. The amidinate can also bridge two metal centers, *i.e.*, in μ_2_-κ^1^N, κ^1^N′ mode. On a metal surface one N-atom can bridge more than two metal atoms and thus a variety of coordination modes results. As the measurements on Pt NPs gave not satisfactory evidence for the bonding mode in ^15^N NMR spectroscopy we turned to different metals for ^15^N NMR spectral data. As ruthenium presents insignificant or no Knight shift, we synthesized Ru/ICy·^(Ph)^NCN NPs as reported before^4^ stabilized with 0.1, 0.2, 0.5 and 1 equiv. of the ^15^N labelled ligand and studied the coordination of the ligand by solid state ^15^N MAS NMR. The resulting Ru/ICy·^(Ph)^NC^15^N NPs have sizes between 1.0 and 1.3 nm, they are very well dispersed and show a narrow size distribution (see ESI, Fig. S11–S14†). As for Pt NPs, we perceived a correlation between the size and the amount of ligand used during the synthesis; the size increases when less ligand is used.

**Fig. 8:**
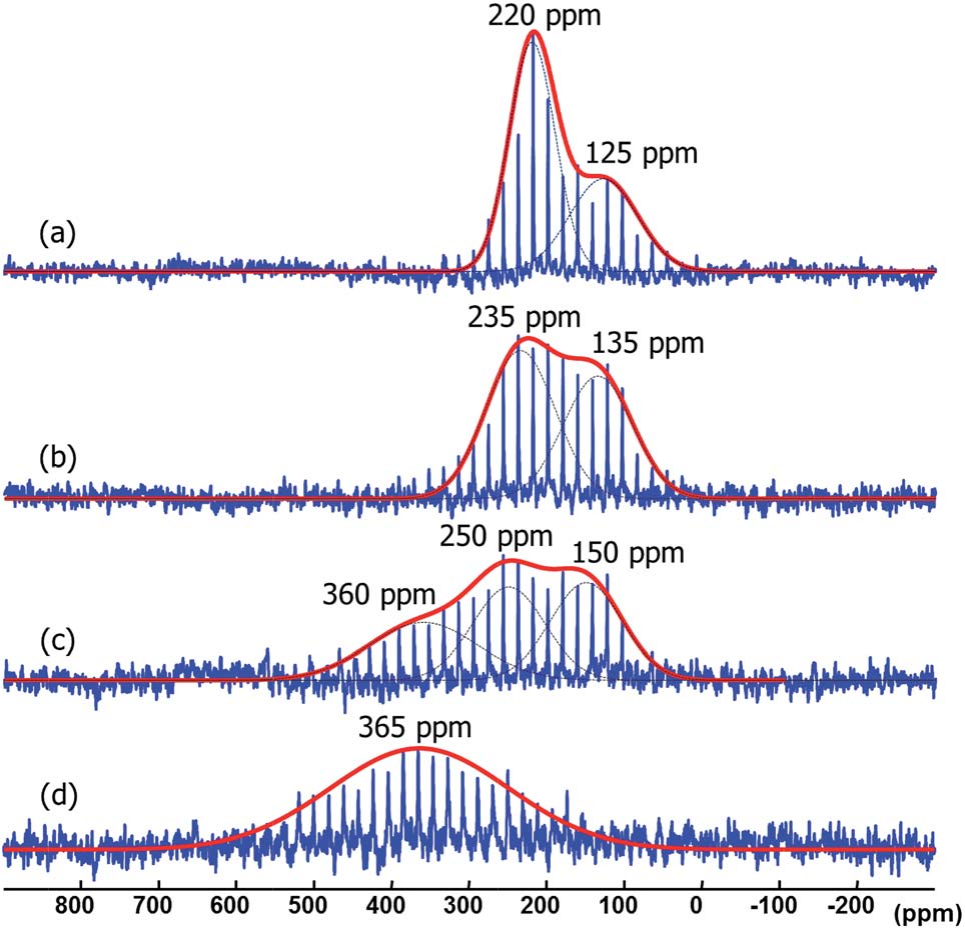
^15^N CPMG MAS NMR of Ru/ICy·^(Ph)^NC^15^N stabilized with (a) 1 equiv., (b) 0.5 equiv., (c) 0.2 equiv. and (d) 0.1 equiv.

In the ^15^N CP/Hahn-echo MAS NMR spectra of Ru/ICy·^(Ph)^NC^15^N NPs (see ESI, Fig. S43†) we can see clearly the peak around 120 ppm corresponding to the protonated amidinate ligand, as was already observed for Pt/CO/ICy·^(Ph)^NC^15^N_0.2_. Furthermore, a new broad featureless signal is observed underneath the ^15^N resonance of the protonated imidazolium-amidinate ligand. Note that for Ru NPs stabilized with 1 equiv. [see ESI, Fig. S43(a)†], we also observed a sharp peak at 212 ppm assigned to the excess of free ligand. ^15^N Hahn-echo MAS NMR spectra (see ESI, Fig. S45, S48, S54 and S57†) allowed the amplification of this new broad ^15^N resonance relative to the signal of the protonated free ligand. Unfortunately, the broadness and the poor signal to noise ratio of these experiments still preclude a clear characterization of the ^15^N signals. In order to increase the sensitivity of the ^15^N MAS experiments, we used the Carr–Purcell–Meiboom–Gill pulse sequence (CPMG).^33^ It works well for NMR resonances that have long spin-state lifetimes but significant inhomogeneous broadening. Fig. 8 displays the ^15^N CPMG MAS NMR spectra of Ru/ICy·^(Ph)^NC^15^N NPs stabilized with the different amounts of ICy·^(Ph)^NC^15^N. With this series of experiments and DFT calculations on the chemical shifts we could establish the different states and coordination modes of the amidinate at the Ru surface. The broad peak centered at *ca.* 250 (±100) ppm corresponds to coordinated ICy·^(Ph)^NC^15^N which can coordinate in different ways. Following a computational strategy successfully used for computing ^1^H and ^13^C chemical shifts, a [Ru_6_] carbonyl cluster was first used as a model for Ru NPs as larger systems cannot be conveniently handled.^34^ The chemical shifts were calculated by DFT studies and compared with the observed ones (see ESI, Fig. S61†). When ICy·^(Ph)^NC^15^N bridges two adjacent Ru atoms, the estimated deltas are 221 and 205 ppm [see ESI, Fig. S61(a)†] (κ^2^ coordination to one metal is not a stable configuration, *vide infra*). For κ^1^ coordination the calculated chemical shifts are 244 ppm for the non-coordinated nitrogen and 118 ppm for the bound one [see ESI, Fig. S61(b)†]. These values change to 262 and 134 ppm if there is π-stacking between the phenyl groups and the ruthenium surface [see ESI, Fig. S61(c)†]. In view of the broadness of the resonances centered at *ca.* 250 ppm (Δ*ν*_1/2_ = 100–300 ppm), the ^15^N NMR spectrum suggests that all three coordination modes are present.

**Fig. 9:**
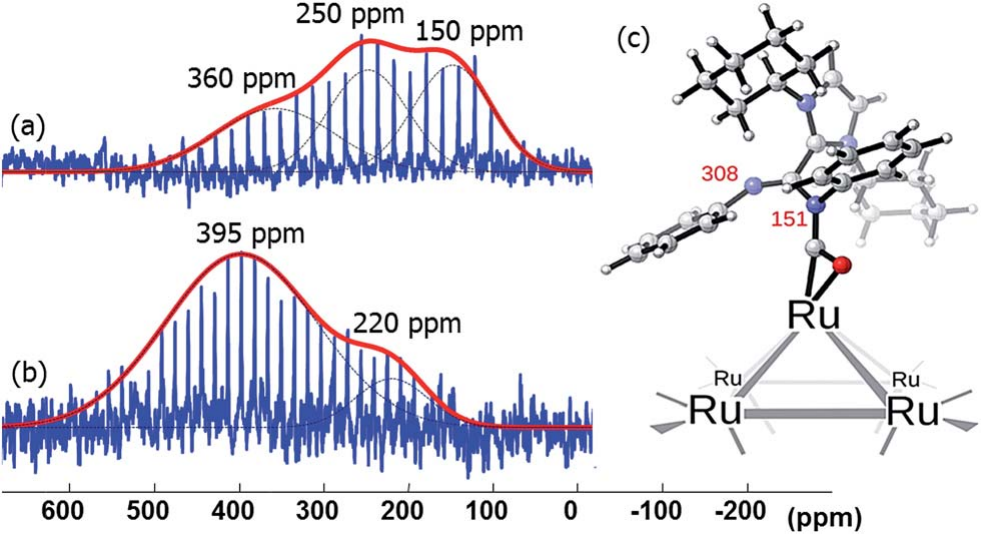
^15^N CPMG MAS NMR of Ru/ICy·^(Ph)^NC^15^N_0.2_ (a) before and (b) after exposure to ^13^CO (1 bar, 20 h, at r.t.). (c) Calculated ^15^N NMR displacements of ICy·^(Ph)^NCN after CO insertion on a [Ru_6_] cluster model.

Lastly, the broad peak observed between 315 and 380 ppm in the ^15^N CPMG MAS NMR spectra of Ru/ICy·^(Ph)^NC^15^N NPs (Fig. 8) is attributed to a species resulting from an insertion reaction of amidinate and carbon monoxide on the surface. This conclusion was corroborated by pressurization of Ru/ICy·^(Ph)^NC^15^N_0.2_ NPs with ^13^CO (1 bar, r.t., 20 h), which led to a significant increase of these resonances (Fig. 9).^35^ Unfortunately, the peak corresponding to CO^ins^, expected at 219 ppm in ^13^C MAS NMR, cannot be observed due to overlap with the broad peak of COb centered at 230 ppm (see ESI, Fig. S51†). The calculated ^15^N shifts of monocoordinated ICy·^(Ph)^NC^15^N after insertion of CO are 151 ppm for the migrated N-atom and 308 ppm for the non-coordinated N-atom (Fig. 9). After a careful analysis of NPA charges on this cluster model, we conclude that this significant unshielding of the latter is due to a delocalization of the negative charge on the CO–Ru fragment and the localization of the π bond between C and this ^15^N-atom (see ESI, Fig. S62 and S63†). A charge analysis on its Ru_55_NP counterpart leads to the same conclusion (see ESI, Fig. S64†). This 308 ppm value is in the range of ^15^N chemical shifts observed for imines.^36^

**Fig. 10:**
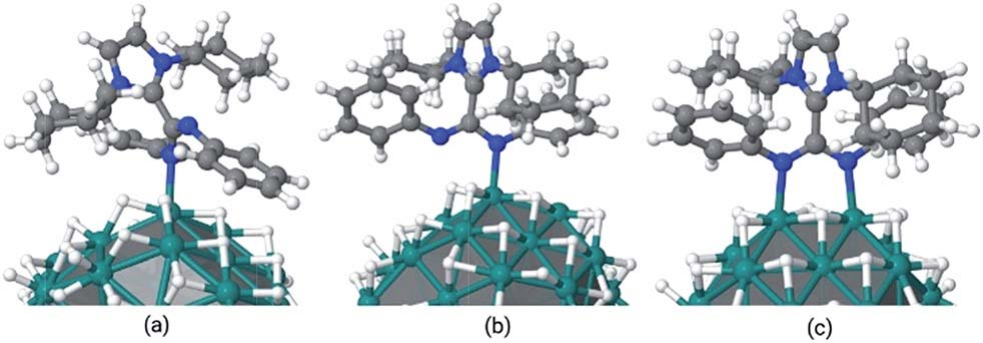
Coordination modes of ICy·^(Ph)^NCN at the Ru_55_H_70_ surface (a) κ^1^N monocoordinated *Z*/*E* conformer (*E*_ads_: –25.0 kcal mol^–1^); (b) κ^1^N monocoordinated *Z*/*Z* conformer (*E*_ads_: –33.3 kcal mol^–1^); (c) μ_2_-κ^1^N, κ^1^N′ bicoordinated *Z*/*Z* conformer (*E*_ads_: –47.9 kcal mol^–1^).

Subsequently, the energetics of ICy·^(Ph)^NCN coordination onto the Ru surface was investigated by DFT calculations. The chosen model is a Ru nanoparticle of 55 atoms with a hydride coverage of 1.6 hydrides per ruthenium surface atom, which has a size of *ca.* 1 nm (Ru_55_H_70_). This model, apart from having a size similar (∼1 nm) to our Ru/ICy·^(Ph)^NCN NPs, has been already satisfactory employed by some of us^11^ to determine the preferred surface composition of Ru NPs as a function of environmental conditions from an application of the *ab initio* thermodynamics method,^37^ that provides a connection between the microscopic and macroscopic regimes. In the present case, the DFT calculations showed that the most stable coordination mode is through the two nitrogen atoms of the amidinate as the *Z*/*Z* conformer to two adjacent Ru-atoms (μ_2_-κ^1^N, κ^1^N′), with a binding energy of –47.9 kcal mol^–1^. The binding of the same conformer through only one N atom has a stability of –33.3 kcal mol^–1^, while that of monocoordination of the *E*/*Z* conformer is –25 kcal mol^–1^ (Fig. 10). When the ligand was initially bound to a single Ru atom as bidentate (κ^2^N, N′), geometry optimization led to a more favorable terminal κ^1^N coordination mode, as observed for some recently reported Cu complexes.^5^ Were the bonding prevailingly polar, such bidentate coordination mode could be favored, as observed for amidinate or carboxylate derivatives of the alkali and rare earth metals.^38^ In summary, the adsorption energy per Ru–N bond of a single amidinate which is not sterically discomforted by its mutual interaction with other ICy·^(Ph)^NCN ligands lies between –24 and –33 kcal mol^–1^.

It is interesting to note that the interaction between the ICy·^(Ph)^NCN ligand and the Ru_55_ nanoparticle does not involve a displacement of the surface hydrides, considering that the loss of four H_2_ molecules is disfavored by +20.6 kcal mol^–1^ (see ESI, Fig. S65†). The maximum number of μ_2_-κ^1^N, κ^1^N′ coordinated ICy·^(Ph)^NCN ligands that we can fit on the Ru_55_ surface is six (see ESI, Fig. S66†), which corresponds with Ru NPs stabilized with 0.1 equiv. of the ligand. The adsorption strength per betaine is lowered from –47.9 kcal mol^–1^ in the case of a single ligand to –29.1 kcal mol^–1^ for the maximum number of them (*i.e.*, 6). For κ^1^ coordinated amidinates one could fit perhaps a few more ligands on the surface, which is still energetically favorable. Thus, the Ru/ICy·^(Ph)^NC^15^N NPs stabilized with 0.2, 0.5 and 1 equiv. have a second sphere of non-coordinated ICy·^(Ph)^NC^15^N which is strongly bonded to the first sphere by ionic and π–π stacking interactions. At least some part of the second sphere is present in the protonated form as indicated by ^15^N NMR.

We shall now evaluate the coordination energy of the species resulting from an insertion reaction of CO into the Ru–N bonds, which we shall call CO^ins^, responsible for the broad peak observed between 315 and 380 ppm in the ^15^N NMR spectrum of carbonylated nanoparticles using the [Ru_55_] cluster model. We initially considered a Ru_55_(CO)_66_ cluster, with a coverage corresponding to 1.5 CO per surface Ru atom, as established in previous *ab initio* thermodynamics calculations.^12^ The adsorption energies of μ_2_-κ^1^N, κ^1^N′ bicoordinated ICy·^(Ph)^NCN, κ^1^N monocoordinated *Z*/*Z* conformer and the CO^ins^ compound are slightly endothermic by 3–5 kcal mol^–1^ whatever the ligand, probably owing to the crowding of carbon monoxides on the surface. This is why we considered the adsorption properties on a Ru_55_(CO)_59_ cluster. The adsorption energy of the CO^ins^ compound becomes exothermic by –27.3 kcal mol^–1^ – a value similar to the Ru–N bond energy – and its grafting mode is similar to what has been obtained on the small [Ru_6_] cluster (see ESI, Fig. S67†). Although its adsorption is strongly exothermic, it is significantly less strongly bound than bicoordinated ICy·^(Ph)^NCN ligands (μ_2_-κ^1^N, κ^1^N′ and κ^2^N, N′) (between –47 kcal mol^–1^ and –76 kcal mol^–1^ depending on the grafting site, *i.e.* between –24 and –38 kcal mol^–1^ per Ru–N bond, close to the values calculated for the Ru_55_H_70_ model). These results suggest that, according to thermodynamics, CO insertion will more likely occur on the monocoordinated amidinate ligands.

In conclusion, the DFT calculations fully support the ^15^NMR measurements in that the Ru NPs are covered with μ_2_-κ^1^N, κ^1^N′ amidinates, κ^1^ coordinated amidinates, and free ligand in part in protonated form. The counter anion of the latter is not known as the synthesis of the MNPs does not involve salts. In the absence of conclusive ^15^NMR measurements for Pt NPs we propose that the coordination mode is the same as that found for Ru.

### Catalytic studies

To probe the catalytic activity and chemoselectivity of the new NPs we chose Pt/ICy·^(*p*-tol)^NCN_0.2_ as model system and tested it in the hydrogenation of several substrates containing various functional groups such as olefinic bonds, carbonyl groups, and aromatic rings. In addition, we investigated the influence of *N*-aryl groups with different electronic properties using Pt NPs ligated to functionalized imidazolium-amidinate ligands (Pt/ICy·^(*p*-anisyl)^NCN_0.2_ and Pt/ICy·^(*p*-ClC_6_H_4_)^NCN_0.2_; see ESI Fig. S15 and S16†). The catalytic results gave interesting differences in terms of activity depending on the type of stabilizing ligand used. In general, all nanocatalysts hydrogenated olefinic and C=O bonds, but, as was expected for Pt NPs, they were not capable of hydrogenating aromatic rings; *e.g.* the hydrogenation of styrene catalyzed by Pt/ICy·^(*p*-tol)^NCN_0.2_, gave selectively ethylbenzene with a maximum turn over frequency (TOF) of 75 300 h^–1^ (Table 1). This high TOF value demonstrates the high catalytic power of this system in hydrogenation of olefinic double bonds. This is peculiar in view of the high coverage with strongly bound imidazolium-amidinate ligands and indicates that these ligands play a favorable role as electron donors.

**Table 1:** Hydrogenation of styrene catalyzed by Pt/ICy·^(*p*-tol)^NCN_0.2_

Entry	Substrate	Products^*c*^	Conv.^*c*^ (%)	TOF (h^–1^)
1^*a*^	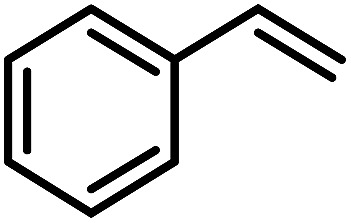	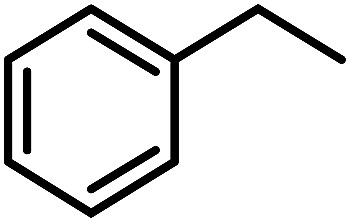	>99	12 800
2^*b*^			10	75 300

^*a*^Reaction conditions: substrate (32 mmol), Pt NPs (0.0025 mmol of Pt assuming 77.3% of Pt from TGA analysis), THF (3 mL), 1 h, *T* = 333 K, *P* = 5 bar.

^*b*^Reaction conditions: substrate (320 mmol), Pt NPs (0.0025 mmol of Pt assuming 77.3% of Pt from TGA analysis), THF (10 mL), 10 min, *T* = 333 K, *P* = 5 bar.

^*c*^Conversions and products identities were determined by ^1^H NMR (average of two runs).

Interestingly, 1.2 nm Pt/CO/ICy·^(Ph)^NC^15^N_0.2_ NPs prepared from Pt_2_(DBA)_3_ and CO (Scheme 3) were totally inactive in the hydrogenation of styrene, due to their surface poisoned by CO. Indeed, as expected from previous works^7,23a^ the IR spectrum of such NPs, recorded immediately after the synthesis, presented two characteristic bands for COt and COb at 2041 and 1859 cm^–1^, respectively which remained essentially unchanged when these are exposed to CO (see ESI, Fig. S24†). This confirmed that the Pt surface of Pt/CO/ICy·^(Ph)^NC^15^N_0.2_ NPs was totally covered with CO due to their synthesis conditions, thus explaining why these NPs are completely inactive as hydrogenation catalyst.

The catalytic behavior of Pt NPs Pt/ICy·^(*p*-anisyl)^NCN_0.2_, Pt/ICy·^(*p*-ClC_6_H_4_)^NCN_0.2_ and Pt/ICy·^(*p*-tol)^NCN_0.2_ was studied in the hydrogenation of 4-phenyl-3-buten-2-one, 3-methyl-2-cyclohexenone and 4-nitrobenzaldehyde (Table 2, entries 1–9). All reactions were carried out with Pt NPs synthesized with 0.2 equiv. of the corresponding amidinate ligand as the results changed little for Pt NPs prepared with 0.1 or 0.5 equiv. (Table S2†). In all cases, the olefins were smoothly hydrogenated (in less than 1 h), but the carbonyl groups reacted more slowly. After 20 h of reaction, ketone and aldehyde groups were partially hydrogenated in different grades depending on the *N*-aryl group of the ligand. We observed a slight increase in activity with the electron-donor strength of these substituents. Thus, the NPs stabilized with the strongest donor ligand, Pt/ICy·^(*p*-anisyl)^NCN_0.2_, were the most active systems in the hydrogenation of the carbonyl group. This trend was more pronounced for ethyl pyruvate (Table 2, entries 10–12). Significant differences of reactivity between the three nanosystems were observed in the hydrogenation of this substrate. This ligand effect was confirmed in the hydrogenation of 2,2,2-trifluoroacetophenone (Table 2, entries 13–15), for which also the Pt NPs ligated by the stronger donor ligand ICy·^(*p*-anisyl)^NCN provided the most active nanocatalyst for this reaction. We conclude that more electron-rich Pt NPs yield faster catalysts, but as yet we cannot speculate on the mechanism.

**Table 2:** Hydrogenation reactions catalyzed by Pt/ICy·^(*p*-anisyl)^NCN_0.2_, Pt/ICy·^(*p*-tol)^NCN_0.2_ and Pt/ICy·^(*p*-ClC_6_H_4_)^NCN_0.2_

Entry/Pt NPs	Substrate	Products^*b*^	Conv.^*c*^ (%), select.^*c*^
1/Pt/ICy·^(*p*-anisyl)^NCN_0.2_ ^*a*^	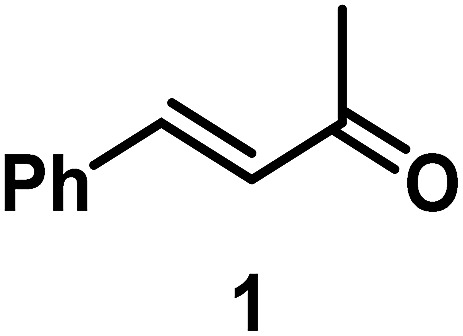	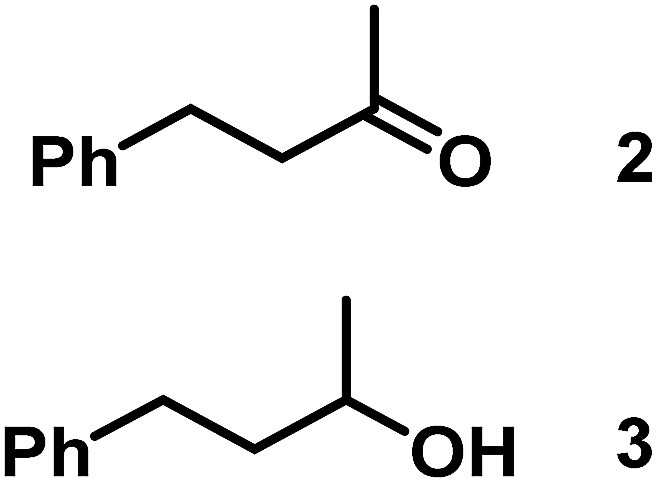	>99, **2/3** = 79 : 21
2/Pt/ICy·^(*p*-tol)^NCN_0.2_ ^*a*^			>99, **2/3** = 81 : 19
3/Pt/ICy·^(*p*-ClC_6_H_4_)^NCN_0.2_ ^*a*^			>99, **2/3** = 86 : 14
4/Pt/ICy·^(*p*-anisyl)^NCN_0.2_ ^*a*^	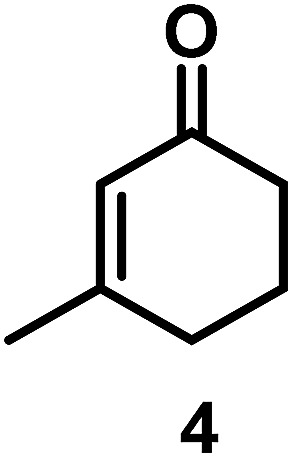	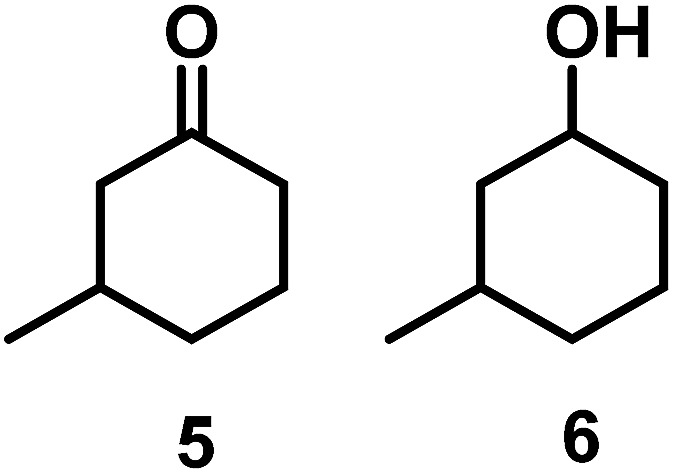	>99, **5/6** = 67 : 33
5/Pt/ICy·^(*p*-tol)^NCN_0.2_ ^*a*^			>99, **5/6** = 68 : 32
6/Pt/ICy·^(*p*-ClC_6_H_4_)^NCN_0.2_ ^*a*^			>99, **5/6** = 78 : 22
7/Pt/ICy·^(*p*-anisyl)^NCN_0.2_ ^*a*^	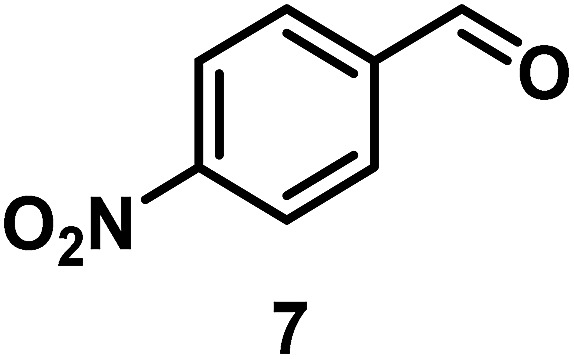	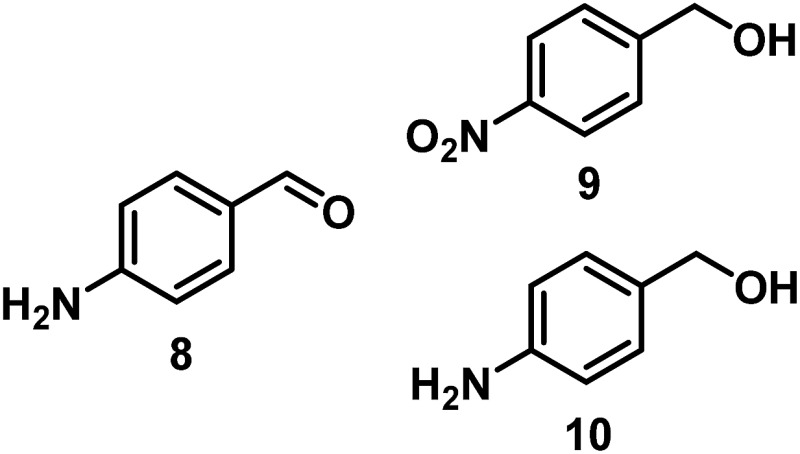	98, **8/9/10** = 52 : 2 : 44
8/Pt/ICy·^(*p*-tol)^NCN_0.2_ ^*a*^			98, **8/9/10** = 53 : 1 : 44
9/Pt/ICy·^(*p*-ClC_6_H_4_)^NCN_0.2_ ^*a*^			98, **8/9/10** = 56 : 4 : 38
10/Pt/ICy·^(*p*-anisyl)^NCN_0.2_ ^*b*^	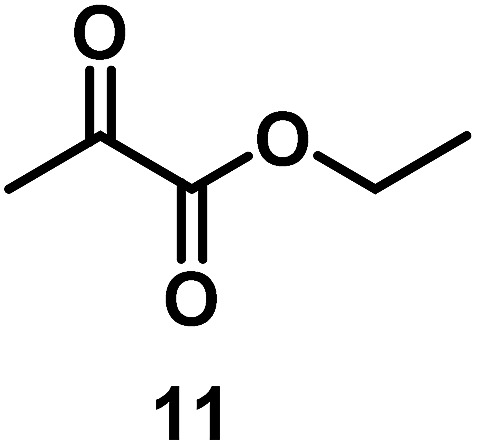	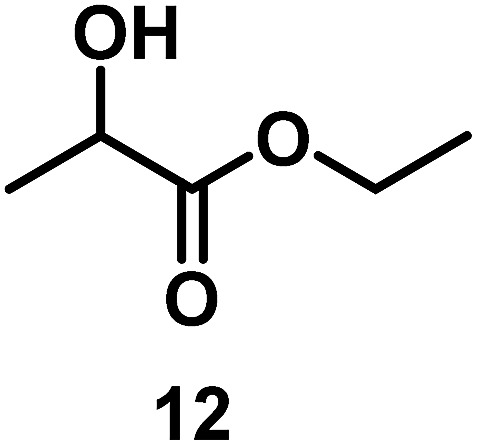	99
11/Pt/ICy·^(*p*-tol)^NCN_0.2_ ^*b*^			86
12/Pt/ICy·^(*p*-ClC_6_H_4_)^NCN_0.2_ ^*b*^			24
13/Pt/ICy·^(*p*-anisyl)^NCN_0.2_ ^*b*^	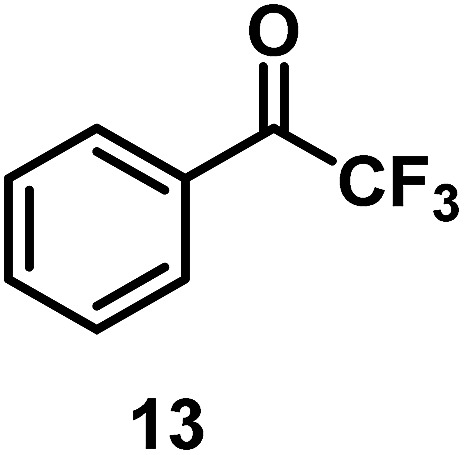	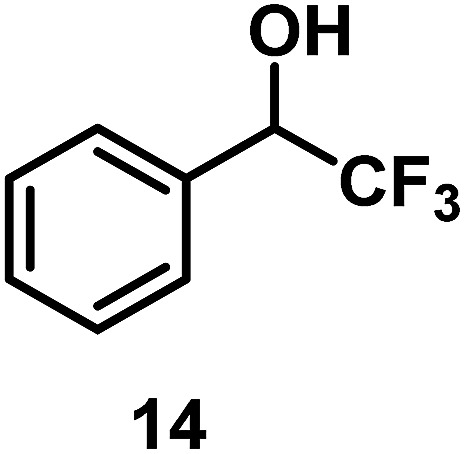	96
14/Pt/ICy·^(*p*-tol)^NCN_0.2_ ^*b*^			47
15/Pt/ICy·^(*p*-ClC_6_H_4_)^NCN_0.2_ ^*b*^			33

^*a*^Reaction conditions: substrate (0.5 mmol), Pt NPs (0.0025 mmol of Pt assuming 77.3% of Pt from TGA analysis), THF (0.75 mL), 20 h, r.t., 5 bar.

^*b*^Reaction conditions: substrate (0.5 mmol), Pt NPs (0.0025 mmol of Pt assuming 77.3% of Pt from TGA analysis), THF (0.75 mL), 3 h, r.t., 5 bar.

^*c*^Conversions, selectivities and products identities were determined by ^1^H NMR (average of two runs).

## Conclusions

We have successfully ligated zwitterionic amidinates to Pt NPs which leads to the peculiar property that the charges of the anions need no compensation by cationic metal ions as in clusters that contain thiolates on the surface for instance. Usually N-based ligands are weakly binding ligands to MNPs, but unexpectedly amidinates bind strongly to the Pt surface atoms. We have shown that ^15^N NMR spectroscopy can be used for the study of the adsorption mode of the nitrogen ligand to the surface with the use of ^15^N enriched ligands. The disturbing Knight shifts on the chemical shifts of the surface bound nuclei could be largely suppressed when small Pt NPs were used. Amidinates may well find broader application in MNP and metal nanocluster (MNC) synthesis and catalysis. Even more so because subtle electronic modification of the *N*-aryl groups of the amidinates has an effect on the catalytic performance of the Pt NPs.
