# Supplementary material for: Zwitterionic amidinates as effective ligands for platinum nanoparticle hydrogenation catalysts
Source: Chem Sci. 2017 Feb 1;8(4):2931–41. doi: 10.1039/c6sc05551f (PMC5376718; doi:10.1039/c6sc05551f)
Supplement: Supplementary file 1 [file SC-008-C6SC05551F-s001.pdf]

Electronic Supplementary Material (ESI) for Chemical Science.
This journal is © The Royal Society of Chemistry 2017

† Electronic Supporting Information

Zwitterionic amidinates as effective ligands for platinum nanoparticle hydrogenation catalysts

L. M. Martínez-Prieto,[a,*] I. Cano,[a] A. Márquez,[b] E. A. Baquero,[a] S. Tricard,[a] L. Cusynato,[a] I. del Rosal,[a] R. Poteau,[a] Y. Coppel,[c] K. Philippot,[c] B. Chaudret,[a] J. Campora[b,*] and P. W. N. M. van Leeuwen[a,*]

[a] LPCNO; Laboratoire de Physique et Chimie des Nano-Objets, UMR5215 INSA-CNRS-UPS, Institut des Sciences appliquées, 135, Avenue de Rangueil, F-31077 Toulouse, France.

[b] Instituto de Investigaciones Químicas, CSIC-Universidad de Sevilla. C/ Américo Vespucio, 49, 41092 Sevilla, Spain.

[c] CNRS, LCC (Laboratoire de Chimie de Coordination), 205 route de Narbonne, BP 44099, F-31077-Toulouse Cedex 4, France ; Université de Toulouse, UPS, INPT, F-31077-Toulouse Cedex 4, France.

Table of contents

S1. Experimental section……S2
S2. TEM data……S15
S3. WAXS data……S19
S4. FT-IR data……S21
S5. Analytical data……S25
S6. Catalytic data……S25
S7. MAS NMR data……S26
S8. DFT data……S42
S9. XPS data……S46

Experimental section

General, materials and characterization techniques.

All chemical operations were carried out using standard Schlenk tubes, Fischer–Porter bottle techniques or in a glove-box under argon atmosphere. Solvents were purified before use: THF (Sigma-Aldrich) by distillation under argon atmosphere and pentane (SDS) by filtration on adequate column of a purification apparatus (MBraun).

$Pt_2(DBA)_3$ was purchased from Strem, CO from Air liquide, ^{13}CO (^{13}C, 99.14%) from Eurisotop, styrene (99%), and 4-phenyl-3-buten-2-one (99%), 4- nitrobenzaldehyde (98%), 3-methyl-2-cyclohexenone (98%), and ethyl pyruvate (98%) from Sigma Aldrich. All reagents were used without purification. $Pt(NBE)_3$ was synthesized by modifying the reported procedure[1] as follows: in a 500-mL three-necked round-bottomed flask fitted with an addition funnel, and a magnetic stirring bar, diethylether (30 mL) was added to a mixture of $PtCl_2COD$ (COD= 1,5-cyclooctadiene)[2] (3.3554 g, 8.972 mmol) and 2-norbornene (6.500 g, 69.09 mmol). The resulting white suspension was stirred in a cold bath at -78 °C. After that, a freshly prepared solution of (1,3,5,7-cyclooctatetraene)dilithium (40 mL, 0.24 M in diethyl ether) was transferred with a syringe to the addition funnel and then added over a 30 minutes period to the stirred white suspension while the temperature was maintained at -78 °C. After that, the resulting light brown suspension was stirred at -78 °C during 30 additional minutes. The reaction mixture was then allowed to warm to room temperature and the volatile material removed under vacuum until dryness (2 h at 0.04 mbar). Then, three extractions with hexane (30 mL each) were carried out over the brown solid. The extract was then filtered through a column of activated alumina (5 x 0.6 cm) under argon atmosphere. The colorless filtrate obtained was evaporated under vacuum until dryness. The platinum complex was then obtained as a white solid and dried during 1 h under vacuum at room temperature (2.610 g, 61% yield). The product was stored in a 2-norbornene atmosphere.

Thermal Analyses (TGA) and Elemental Analysis. TGA analyses were performed in a TGA/DSC 1 STAR System equipped with an ultra-microbalance UMX5, a gas switch GC200 and sensors DTA and DSC. The samples were analyzed through a two steps oxidation/reduction method. First the sample was heated from 25 °C to 500 °C at 10 °C/min under air (2h). After cooling down, it was heated again from 25 °C to 700 °C at 30°C/min under a gas mixture Ar/H_2 4% (3h). Elemental analyses were performed at "Mikroanalytisches Labor Kolbe" in Mülheim.

---

[1] Crascall, L. E.; Spencer, J. L.; Doyle, R. A.; Angelici, R. J. *Inorg. Synth.*, **1990**, *28*, 126.

[2] (a) Tronnier, A.; Poethig, A.; Herdtweck, E.; Strassner, T.; *Organometallics*, **2014**, *33*, 898. (b) McDermott, J. X.; White, J. F.; Whitesides, G. M.; *J. Am. Chem. Soc.* **1976**, *98*, 6521.

Wide-angle X-ray scattering (WAXS). WAXS was performed at CEMES-CNRS. Samples were sealed in 1.0 mm diameter Lindemann glass capillaries. The samples were irradiated with graphite monochromatized molybdenum Kα (0.071069 nm) radiation and the X-ray intensity scattered measurements were performed using a dedicated two-axis diffractometer. Radial distribution functions (RDF) were obtained after Fourier transformation of the reduced intensity functions.

Nuclear Magnetic Resonance (NMR). ^{1}H spectra were recorded on a Bruker Avance 400 spectrometer.

Solid state NMR (MAS-NMR). Solid-state NMR experiments were recorded at the LCC (Toulouse) on a Bruker Avance 400 spectrometer equipped with 3.2 mm probes. Samples were spun between 14 to 20 kHz at the magic angle using ZrO_2 rotors. ^{13}C MAS experiments were performed with a recycle delay of 20 s. ^{13}C CP/MAS spectra were recorded with a recycle delay of 2 s and a contact time of 4 ms. ^{15}N MAS experiments were performed with a recycle delay of 1s. ^{15}N CP-MAS spectra were recorded with a recycle delay of 2s and a contact time of 3ms. Hahn-echo scheme were synchronized with the spinning rate. ^{15}N CPMG were acquired with 50 echoes, a delay between train of 180° pulse of 10 rotor periods and a recycle delay of 1s.

Transmission Electron Microscopy (TEM) and High resolution TEM (HRTEM). Pt NPs were observed by TEM and HRTEM after deposition of a drop of a solution of the isolated nanoparticles after dispersion in THF on a copper grid. TEM analyses were performed at the UMS-Castaing by using a JEOL JEM 1011 CX-T electron microscope operating at 100 kV with a point resolution of 4.5 Å. The approximation of the particles mean size was made through a manual analysis of enlarged micrographs by measuring a number of particles on a given grid. HRTEM observations were carried out with a JEOL JEM 2010 electron microscope working at 200 kV with a resolution point of 2.35 Å. FFT treatments have been carried out with Digital Micrograph Version 3.7.4.

Infrared spectroscopy (IR). ATR IR-FT spectra were recorded on a Thermo Scientific Nicolet 6700 spectrometer in the range 4000–600 cm^{-1}.

X-Ray Photoelectron Spectroscopy (XPS). XPS analyses were performed at CIRIMAT Laboratory (Toulouse) using a Thermoelectron Kalpha device. The photoelectron emission spectra were recorded using Al-Kα radiation (hv= 1486.6 eV) from a monochromatized source. The analyzed area was about 0.15 mm^2. The pass energy was fixed at 40 eV. The spectrometer energy calibration was made using the Au $4f_{7/2}$ (83.9 ± 0.1 eV) and $Cu2p_{3/2}$ (932.8 ± 0.1 eV) photoelectron lines. XPS spectra were recorded in direct mode N(Ec). The background signal was removed using the Shirley method. The atomic

concentrations were determined from photoelectron peak areas using the atomic sensitivity factors reported by Scofield, taking into account the transmission function of the analyzer. The photoelectron peaks were analyzed by Gaussian/ Lorentzian (G/L=50) peak fitting.

DFT calculations. All DFT calculations on the [Ru_6] clusters were performed with Gaussian09.[3] Geometries were fully optimized in gas phase without symmetry constraints, employing the B3PW91 functional[4] and the Stuttgart effective core potential for Ru,[5] augmented with a polarization function (ζ_f=1.235). For the other elements (H, C, O, and P), Pople's double-ζ basis set 6-31G(d,p)[6] was used. Calculations of vibrational frequencies were systematically done in order to characterize the nature of stationary points. Among the various theories available to compute chemical shielding tensors, the Gauge Including Atomic Orbital (GIAO) method has been adopted for the numerous advantages it presents. Calculating a theoretical chemical shift requires the knowledge of the chemical shielding of a reference, since it is explicitly calculated as $\delta = (\sigma_{ref} - \sigma)$, in ppm. We have shown in previous studies on ruthenium clusters that DFT-GIAO provides ^1H and ^{13}C chemical shifts in excellent agreement with experiments.[7] The experimental reference chemical shift for ^{15}N corresponds to ammoniac in its liquid phase. The theoretical magnetic shielding for NH_3 has been calculated using the same strategy as in Ref. [8] (ab initio molecular dynamic simulations combined with the calculations of magnetic shielding on extracted cluster's structures from molecular dynamics). We have also used the Natural Population Analysis (NPA)[9] included in the Natural Bond Orbital (NBO)[10] routines available in Gaussian09, yielding the often called "NBO charges".

[3] Frisch, M. J.; Trucks, G. W.; Schlegel, H. B.; Scuseria, G. E.; Robb, M. A.; Cheeseman, J. R.; Scalmani, G.; Barone, V.; Mennucci, B.; Petersson, G. A.; Nakatsuji, H.; Caricato, M.; Li, X.; Hratchian, H. P.; Izmaylov, A. F.; Bloino, J.; Zheng, G.; Sonnenberg, J. L.; Hada, M.; Ehara, M.; Toyota, K.; Fukuda, R.; Hasegawa, J.; Ishida, M.; Nakajima, T.; Honda, Y.; Kitao, O.; Nakai, H.; Vreven, T.; Montgomery, Jr., J. A.; Peralta, J. E.; Ogliaro, F.; Bearpark, M.; Heyd, J. J.; Brothers, E.; Kudin, K. N.; Staroverov, V. N.; Kobayashi, R.; Normand, J.; Raghavachari, K.; Rendell, A.; Burant, J. C.; Iyengar, S. S.; Tomasi, J.; Cossi, M.; Rega, N.; Millam, J. M.; Klene, M.; Knox, J. E.; Cross, J. B.; Bakken, V.; Adamo, C.; Jaramillo, J.; Gomperts, R.; Stratmann, R. E.; Yazyev, O.; Austin, A. J.; Cammi, R.; Pomelli, C.; Ochterski, J. W.; Martin, R. L.; Morokuma, K.; Zakrzewski, V. G.; Voth, G. A.; Salvador, P.; Dannenberg, J. J.; Dapprich, S.; Daniels, A. D.; Farkas., &O.; .; Foresman, J. B.; Ortiz, J. V.; Cioslowski, J. & Fox, D. J. Gaussian 09 Revision D.01

[4] Becke, A. D. *J. Chem. Phys*. **1993**, *98*, 5648 *and references therein.*

[5] Küchle, W.; Dolg, M.; Stoll, H. & Preuss, H. *Mol. Phys*. **1991**, *74*, 1245.

[6] (a) Hariharan, P. C. & Pople, J. A. *Theor. Chem. Acc*. **1973**, *28*, 213. (b) Hehre, W. J.; Ditchfield, R. & Pople, J. A. *J. Chem. Phys*. **1972**, *56*, 2257.

[7] (a) del Rosal, I.; Maron, L.; Poteau, R.; Jolibois, F. *Dalton Trans.* **2008**, 30, 3959. (b) del Rosal, I.; Gutmann, T.; Maron, L.; Jolibois, F.; Chaudret, B.; Walaszek, B.; Limbach, H.H.; Poteau, R.; Buntkowsky. G. *Phys. Chem. Chem. Phys.* **2009**, 11, 5657. (b) del Rosal, I.; Jolibois, F.; Maron, L.; Philippot, K.; Chaudret, B.; Poteau, R. *Dalton Trans.* **2009**, 12, 2142.

[8] Gerber, I. C. & Jolibois, F. *Phys. Chem. Chem. Phys*. **2015**, *17*, 12222.

[9] Reed, A. E.; Weinstock, R. B. & Weinhold, F. *J. Chem. Phys*. **1985**, *83*, 735.

[10] (a) Reed, A. E.; Curtiss, L. A. & Weinhold, F. *Chem. Rev*. **1988**, *88*, 899. (b) Weinhold, F. & Landis, C. R. *Chem. Educ. Res. Pract. Eur*. **2001**, *2*, 91.

DFT calculations on the [Ru_{55}] hcp nanoparticles were performed with the Vienna ab initio simulation package, VASP[11] within the framework of density functional theory. Projector augmented waves (PAW)[12] were used, with a plane-wave kinetic energy cutoff of 500 eV. All the calculations used the Perdew-Burke-Ernzerhof form of the generalized gradient approximation.[13] The supercell used was 27 x 27.5 x 28 Å large, ensuring at least 16 Å of vacuum between to successive images of ligand-covered Ru_{55}. Γ-centered[14] calculations were performed with a Gaussian smearing (σ) of 0.02 eV, the energies being therefore extrapolated for σ=0.00 eV. The atoms positions were optimized until the criterion of the residual forces on any direction being less than 0.02 eV/Å was met. Also Reliable atom-projected density of states (pDOS), directly based on plane-wave DFT output as given by the VASP package, were computed with the Lobster package.[15]

Synthesis of zwitterionic imidazolium-amidinates

General Considerations.

Carbodiimides $RC_6H_4N{=}C{=}NC_6H_4R$ were obtained from the corresponding thioureas according to the procedure reported by Patel.[16] The synthesis of diphenylurea (from phenylisocyanate and aniline) and diphenylcarbodiimide is described in ref. 3. Bis-*p*-methoxythiourea was synthesized from thiophosgene and *p*-anisidine.[17] We also prepared bis *p*-chlorophenylthiourea using the latter procedure, albeit a synthesis from CS_2 has been described recently.[18] Phenyl isothiocyanate, anilines and 98% aniline-^{15}N were purchased from Aldrich and used without further purification. 1,3-Dicyclohexylimidazolium tetrafluoborate was prepared by a literature method.[19] The synthesis of imidazolium-amidinate ligands was carried out under inert argon atmosphere using standard Schlenk techniques.

Synthesis of ICy·$^{(Ph)}$NCN: 16.2 mL of a 0.5 M solution of ^{t}BuOK in THF (8.1 mmol) were slowly added to a stirred suspension of 2.56 g (8.1 mmol) of N,N-dicyclohexylimidazolium tetrafluoborate in 20 mL of the same solvent, cooled to -80 ºC. The cooling bath was removed and the stirring was continued for 30 min at r.t. The mixture was cooled again to -80 ºC and a solution of diphenylcarbodiimide (1.58 g, 8.1 mmol) in 10 mL of THF was added dropwise. As the carbodiimide reacts with the heterocyclic

[11] (a) Kresse, G.; Fürthmuller, J. *Phys. Rev. B* **1996**, *54*, 11169. (b) Kresse, G.; Fürthmuller, J. *Comput. Mater. Sci.* **1996**, *6*, 15.
[12] (a) Blöchl, P. E. Phys. Rev. B 1994, 50, 17953. (b) Kresse, G.; Joubert, D. Phys. Rev. B 1999, 59, 1758.
[13] Perdew, J. P.; Burke, K. & Ernzerhof, M. *Phys. Rev. Lett*. **1997**, *78*, 1396.
[14] Monkhorst, H. J. & Pack, J. D. *Phys. Rev. B* **1976**, *13*, 5188.
[15] (a) Dronskowski, R.; Blöchl, P. E. *J. Phys. Chem*. **1993**, *97*, 8617. (b) Deringer, V. L.; Tchougréeff, A. L.; Dronskowski, R. *J. Phys. Chem. A* **2011**, *115*, 5461. (c) Maintz, S.; Deringer, V. L.; Tchougréeff, A. L.; Dronskowski, R. *J. Comp. Chem*. **2013**, *34*, 2557.
[16] Ali, A. R.; Ghosh, H.; Patel, B. K. *Tetrahedron Lett.* **2010**, *51*, 1019.
[17] Kuhn, N.; Steinman, M.; Weyers, G.; Henkel, G. *Z. Naturforsch., B: J. Chem. Sci.* **1999**, *54*, 434.
[18] Zhao, D.; Liu, H.; Zheng, L.; He, G.; Qu, D.; Han, S. *Med. Chem. Res.* **2013**, *22*, 3743.
[19] Archer, R. H.; Carpenter, J. R.; Hwang, S. J.; Burton, A. W.; Chen, C. Y.; Zones, S. L.; Davis, M. E. *Chem. Mater.* **2010**, *22*, 2563.

carbene, a yellow color develops. The mixture was allowed to stir at the r.t. for 2h, and then was taken to dryness under reduced pressure. Extraction in dichloromethane, and filtration through a celite pad afforded a clear yellow solution, which was evaporated. The solid residue was washed with 20 mL of diethyl ether, leaving the product as a yellow powder (1.90 g, 4.5 mmol, 55.0 % yield). ^{1}H NMR (400 MHz, CD_2Cl_2, 25ºC): δ 1.18 (m, 2H, Cy 4CH_{eq}H); 1.42 (m, 8H, Cy 3CHH_{ax}+2CHH_{ax}); 1.71 (d, $^{2}J_{HH}$ = 12 Hz, 2H, Cy 4CHH_{ax}); 1.83 (d, 4H, $^{2}J_{HH}$ = 12 Hz, 2H, Cy 3CH_{eq}H); 1.87 (v br s, 4H, Cy 2CH_{eq}H); 4.79 (m, 2H, Cy 1C*H*); 6.72 (t, 2H, $^{2}J_{HH}$ = 7.2 Hz, Ph *p*-H); 7.03 (s, 2H, Imdz C*H*); 7.05 (br s, 4H, Ph *o*-H); 7.14 (t, 4H, $^{2}J_{HH}$ = 7.2 Hz, Ph *m*-H). ^{13}C{^{1}H} NMR (100 MHz, CD_2Cl_2, 25ºC): δ 25.4 (Cy 4CH_2); 25.7 (Cy 3 CH_2); 33.5 (br, Cy 2 CH_2); 58.7 (Cy 1 *C*H); 118.5 (Imdz *C*H); 119.4 (Ph *p*-*C*H); 129.1 (br, Ph *o*-CH); 128.6 (Ph *m*-*C*H); 145.1 (C_q(NPh)$_2$); 146.4 (Imdz C_q); 153.2 (Ph *ipso*-C_q). IR (Nujol mull, cm^{-1}): 1587 w, 1561 w, 1561 st ($\upsilon_{C=N}$). ESI-ES (from dichloromethane): m/z 427.4 ([M+H]$^+$). Elemental Analysis Calcd. for $C_{28}H_{34}N_4$: C, 78.83, H 8.03; N, 13.13; Found, C, 78.47; H, 8.20; N, 13.34.

Synthesis of ICy$^{\cdot(p\text{-}anisyl)}$NCN: This compound was prepared similarly, starting from 0.76 g (3 mmol) of bis-*p*-methoxyphenylcarbodiimide. The crude product was purified by recrystallization from a CH_2Cl_2/hexane mixture, affording two crops of 0.43 and 0.19 g of yellow crystals (1.26 mmol, 42.1 % yield). ^{1}H NMR (400 MHz, CD_2Cl_2, 25ºC): δ 1.18 (m, 2H, Cy 4CH_{eq}H); 1.42 (m, 8H, Cy 3CHH_{ax} + 2CHH_{ax}); 1.71 (d, $^{2}J_{HH}$ = 12 Hz, 2H, Cy 4CHH_{ax}); 1.83 (d, 4H, $^{2}J_{HH}$ = 12 Hz, 2H, Cy 3CH_{eq}H); 1.70 (v br s, 4H, Cy 2CH_{eq}H); 3.71 (s, 6H, -OCH_3); 4.57 (m, 2H, Cy 1C*H*); 6.72 (d, 4H, $^{2}J_{HH}$ = 8.5 Hz, Ar *m*-H); 6.99 (br s, 4H, Ar *o*-H); 7.02 (s, 2H, Imdz C*H*). ^{13}C{^{1}H} NMR (100 MHz, CD_2Cl_2, 25ºC): δ 25.4 (Cy 4CH_2); 25.7 (Cy 3 CH_2); 33.5 (br, Cy 2 CH_2); 55.8 (-OCH_3); 58.5 (Cy 1 *C*H); 114.1 (Ar, *m*-*C*H); 116.5 (Imdz *C*H); 123.8 (br, Ar o-*C*H); 144.8 (Cq(NAr)$_2$); 146.9 (br, Imdz C_q + Ar ipso-C_q-N); 153.6 (Ar *p*-C_q-OMe). IR (Nujol mull, cm^{-1}): 1587 w, 1579 m, 1562 m ($\upsilon_{C=N}$). ESI-ES (from dichloromethane): m/z 487.5 ([M+H]$^+$). Elemental Analysis Calcd. for $C_{30}H_{38}N_4O_2$: C, 74.04, H 7.87; N, 11.51; Found, C, 70.52; H, 7.84; N, 10.60.

Synthesis of ICy$^{\cdot(p\text{-}ClC_6H_4)}$NCN: This compound was prepared similarly, starting from 1.00 g (3.8 mmol) of bis-*p*-chlorophenylcarbodiimide. The crude product was purified by recrystallization from a CH_2Cl_2/hexane mixture, affording 0.62 g of yellow crystals (1.26 mmol, 33.0 % yield). ^{1}H NMR (400 MHz, CD_2Cl_2, 25ºC): δ 1.18 (m, 2H, Cy 4CH_{eq}H); 1.42 (m, 8H, Cy 3CHH_{ax} + 2CHH_{ax}); 1.71 (d, $^{2}J_{HH}$ = 12 Hz, 2H, Cy 4CHH_{ax}); 1.84 (d, 4H, $^{2}J_{HH}$ = 12 Hz, 2H, Cy 3CH_{eq}H); 1.86 (v br s, 4H, Cy 2CH_{eq}H); 4.54 (m, 2H, Cy 1C*H*); 6.97 (br s, 4H, Ar *o*-H); 7.02 (s, 2H, Imdz C*H*); 7.05 (d, 4H, $^{2}J_{HH}$ = 8.5 Hz, Ar *m*-H). ^{13}C{^{1}H} NMR (100 MHz, CD_2Cl_2, 25ºC): δ 25.3 (Cy 4CH_2); 25.7 (Cy 3 CH_2); 33.6 (br, Cy 2 CH_2); 55.8 (-OCH_3); 58.7 (Cy 1 *C*H); 116.8 (Imdz *C*H); 124.2 (overlapping signal, Ar *ipso*-*C*-N), 124.3 (br, Ar o-*C*H); 128.5 (Ar, *m*-*C*H); 145.1 (Cq(NAr)$_2$); 145.8 (br, Imdz C_q); 151.4 (Ar 4-C_q-Cl). IR (Nujol mull, cm^{-1}): 1588 m, 1575 m, 1562 m ($\upsilon_{C=N}$).

ESI-ES (from methanol): m/z 495.6 ([M+H]$^+$). Elemental Analysis Calcd. for $C_{28}H_{32}Cl_2N_4$: C, 67.87, H 6.51; N, 11.31; Found, C, 68.03; H, 6.15; N, 10.98.

Synthesis of ICy·$^{(Ph)}$NC^{15}N: The labeled ligand was prepared as shown in Scheme S1, starting from 1 g (10.7 mmol) of 98% aniline-15N. The final yield was 2.03 g (4.9 mmol, 69.9 % yield from the labeled thiourea). The product was recrystallized from a dichloromethane/hexane mixture. The IR spectrum (nujol) and solution ^{1}H and ^{13}C{^{1}H} NMR spectra showed no significant differences with the non-labeled compound, except for the slight broadening of the quaternary C atoms directly bound to the "amidinate" N atoms due to unresolved ^{15}N-^{13}C coupling. CP-MAS ^{15}N NMR: two signals in 1:1 ratio at δ 211, 218 ppm corresponding to the no equivalents nitrogen atoms of the *E/Z* conformation. ESI-MS (from dichloromethane): Signal centered at m/z 428.4 for ([M+H]$^+$) with an isotope pattern corresponding to a 1:2:1 mixture of $^{15}N_2$, $^{15}N^{14}N$ and $^{14}N_2$ isotopologues (427, 13.5%; 428, 47.9 %; 429, 30.4%; 430, 7.3%; 431, 0.9%). Elemental Analysis Calcd. for $C_{28}H_{34}N_3{}^{15}N$ (%): C, 78.65; H, 8.01; N, 13.34; Found, C, 78.61; H, 8.82; N, 13.41.

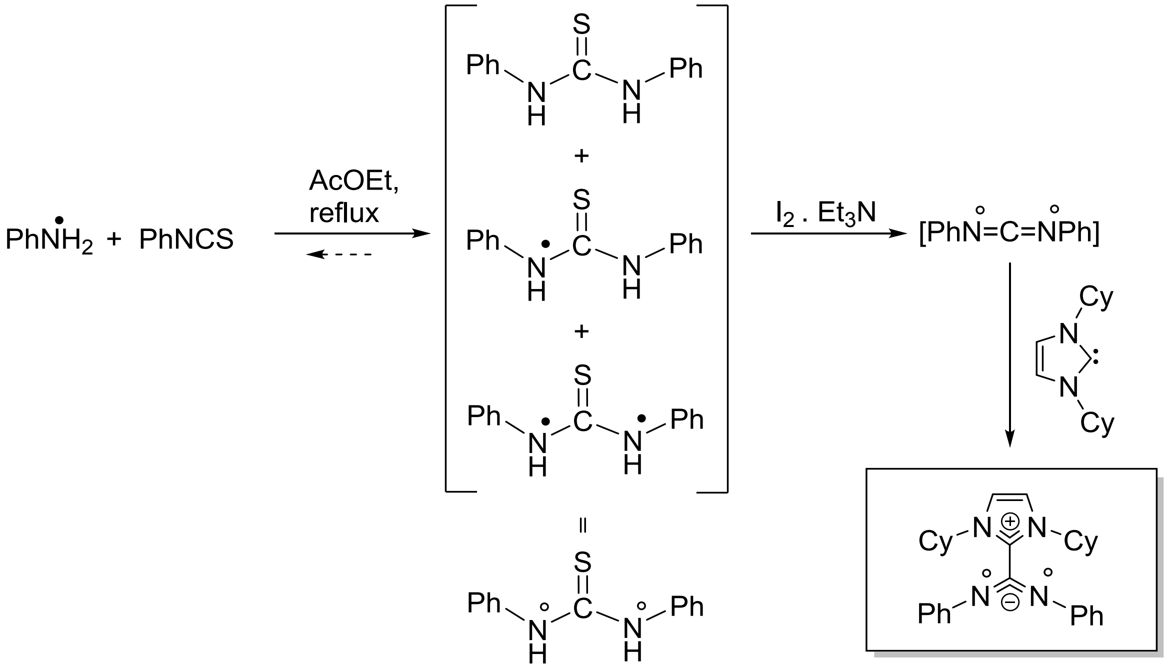

ICy·$^{(Ph)}$NC^{15}N

Sc

heme S1. Synthesis of ICy· $^{(Ph)}$NC^{15}N. Black dots are associated to 1abeled ^{15}N atoms.

The ^{15}N CP-MAS NMR spectrum of the free ligand ICy·$^{(Ph)}$NC^{15}N exhibited two signals of roughly the same intensity at 211 and 218 ppm (Figure 7). X-ray data for a series of ICy·$^{(Ar)}$NCN ligands showed that in the solid state the amidinate fragment invariably adopts an *anti/syn (E/Z)* configuration with regard

to the partial double C=N bonds.[20] However, the ^{1}H and ^{13}C NMR spectra of the ICy·$^{(PhR)}$NCN recorded in CD_2Cl_2 (see experimental section) show a single set of signals for the aromatic groups, indicating that there is a fast exchange between the *E/Z* and *Z/E* configurations of the C-N bonds of the amidinate in solution. At r.t., the signals corresponding to the aromatic ortho-^{13}C and ^{1}H nuclei appear significantly broadened. DFT calculations confirmed that the peaks at 211 and 218 ppm of the Figure S1 are attributed to the two non-equivalent nitrogen atoms of the isomer *E/Z* (Figure S3). Furthermore, the *E/Z* configuration is more stable than the syn/syn (*Z/Z)* by 10.2 kcal.mol^{-1}, a significant energy difference which rules out the possibility of the experimental observation of the *Z/Z* isomer. Thus, for free ICy.(^{Ph})NCN, the Z/Z isomer is only an unstable intermediate on the Z/E inversion pathway that takes place through an accessible transition state located at +18.3 kcal.mol^{-1} with respect to the anti/syn structures. In the same way, in the ^{13}C CP-MAS NMR spectrum, various signals of ICy·$^{(Ph)}$NC^{15}N are duplicated due to this conformation where the phenyls groups are not equivalent (Figure S2). The assignment of this spectrum was also corroborated by theoretical studies (Figure S4).

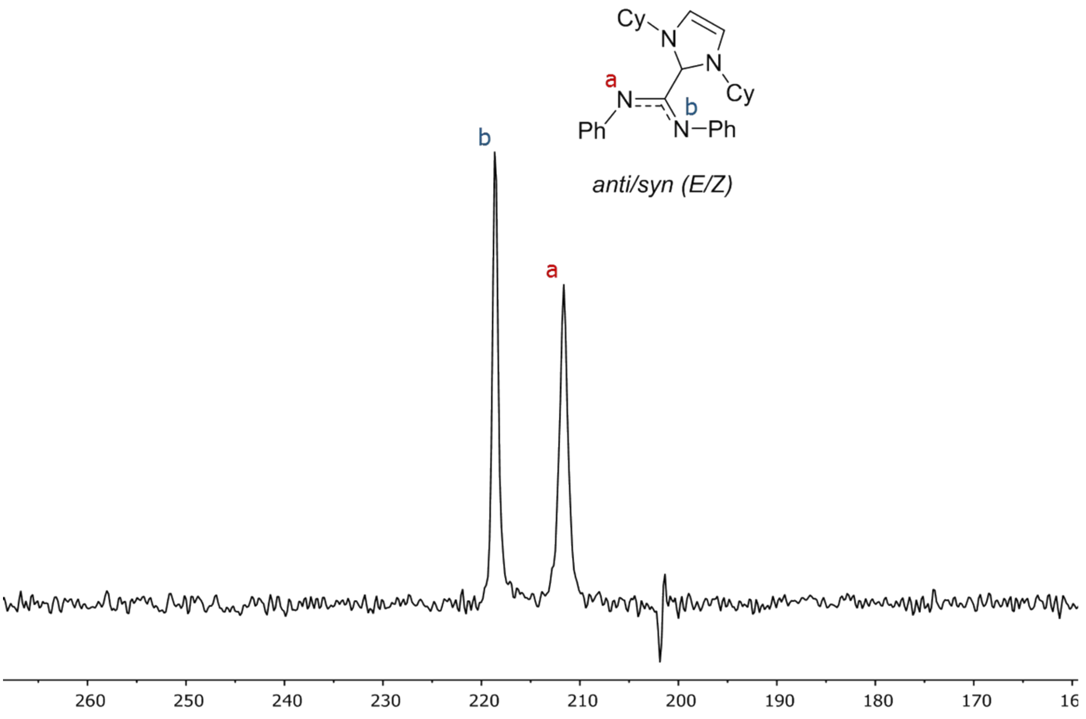

Figure S1. ^{15}N CP-MAS NMR spectrum of E/Z isomer of ICy·$^{(Ph)}$NC^{15}N.

[20] Márquez, A.; Ávila, E.; Urbaneja, C.; Álvarez, E.; Palma, P.; Cámpora J. *Inorg. Chem.* **2015**, *54*, 11007.

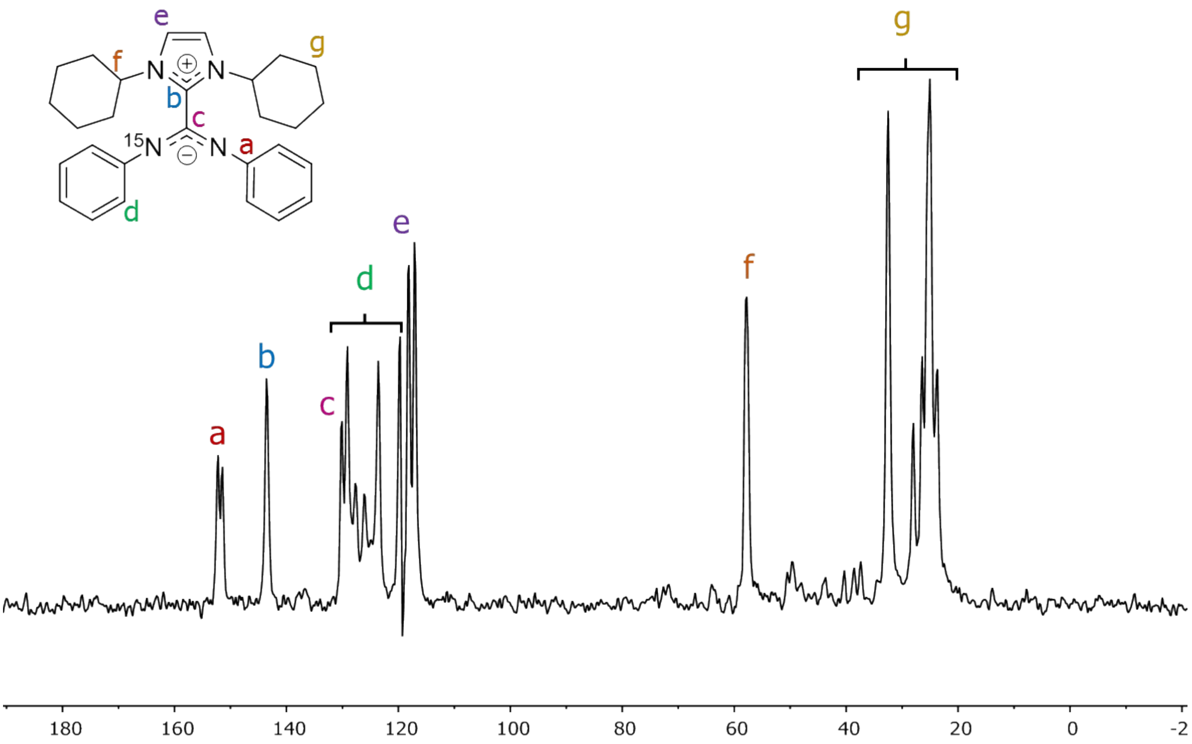

Figure S2. ^{13}C CP-MAS NMR spectrum of ICy$^{\cdot(Ph)}$NC^{15}N.

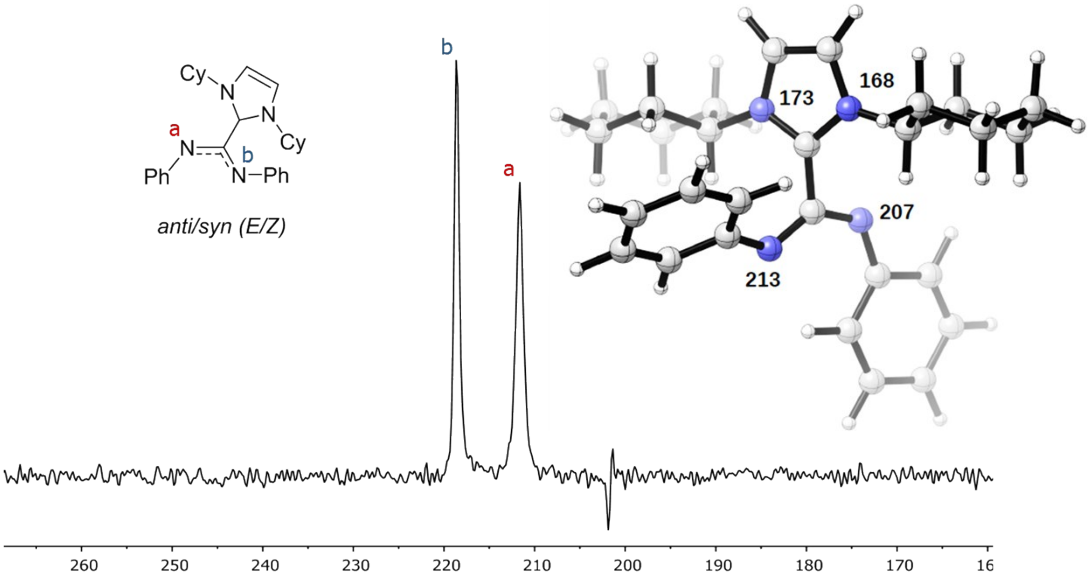

^{15}N signal	Observed (ppm)	Calculated (ppm)
a	218	213
b	211	207

Figure S3. Observed and calculated (at the DFT-B3PW91 level of theory) ^{15}N MAS NMR signals of ICy$^{\cdot(Ph)}$NC^{15}N.

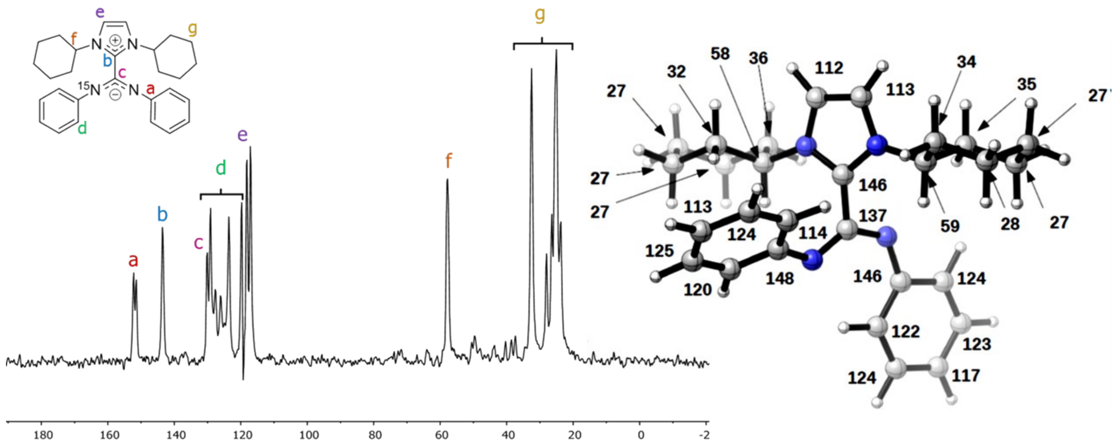

^{13}C signal	Observed (ppm)	Calculated (ppm)
a	152/151	148/146
b	143	146
c	131	137
d	130-120	125-113
e	117/118	112/113
f	58	59/58
g	32-23	36-27

Figure S4. Observed and calculated (at the DFT-B3PW91 level of theory) ^{13}C MAS NMR signals of $ICy^{\cdot(Ph)}NC^{15}N$.

Synthesis of $[H{\cdot}ICy^{\cdot(Ph)}NCN]^{+}\cdot[BPh4]^{-}$: 0.5 mL of a 1 M solution of AcOH in dichloromethane was added drop-wise to a stirred suspension containing of 0.214 g (0.5 mmol) of $ICyCDI^{Ph}$ and 0.717 g of $NaBPh_4$ in 15 mL of the same solvent. As the acid was added, the yellow color of the starting imidazolium-amidinate ligand fades away. The mixture was stirred for 2 h, and any remaining solids were removed by filtration through Celite®. The clear solution was taken to dryness leaving a foamy residue that was converted in a pale yellow solid by stirring with hexane (crude yield, 320 mg, 81 %). This solid was recrystallized from CH_2Cl_2/hexane to afford the product as colorless crystals (76.6 % yield). NMR spectra showed that the cation exists in solution as a 2:1 mixture of *E/Z* and *Z/E* isomers. ^{1}H NMR (400 MHz, CD_2Cl_2, 25ºC): δ 1.1 – 2.0 (m, 20 H 2Cy 2, 3 and 4-C*H*$_2$); 3.94 (m, ~ 0.66 H, Cy 1 *C*H, minor isomer); 4.21(m, ~ 1.33 H, Cy 1 *C*H, major isomer); 6.38 (s, ~ 0.66 H, Imdz C*H*, minor isomer); 6.57 (s, ~ 1.33 H, Imdz C*H*, major isomer); 6.57 (d, ~ 1.33 H, $^{3}J_{HH}$ = 7.6 Hz, NHPh *o*-H, major isomer); 6.89 (t, 4H, $^{3}J_{HH}$ = 7.2 Hz, BPh_4 *p-H*); 7.03 (t, 8H, $^{3}J_{HH}$ = 7.4 Hz, BPh_4 *m-H*); 7.05 (partly hidden br s, ~ 0.66 H, N*H*, minor isomer); 7.19 (br s, ~ 1.33 H, N*H*, major isomer); 7.20 – 7.32 (m, ~1.6 H, *H* arom.); 7.29 (d, $^{3}J_{HH}$ = 8.0 Hz, N-Ph *o*-H, major+minor isomers); 7.35 (m, 8H, BPh_4 *o-H*); 7.52 (t, $^{3}J_{HH}$ = 7.9 Hz, N-Ph *m*-H, major+minor isomers). $^{13}C\{^{1}H\}$ NMR (100 MHz, CD_2Cl_2, 25ºC): δ 24.7 (Cy 4*C*H$_2$, minor isomer); 24.8 (Cy 4*C*H$_2$, major

isomer); 25.5 (Cy 3CH_2, minor isomer); 25.7 (Cy 4CH_2, major isomer); 33.38 (br, Cy 2CH_2, major + minor isomer); 60.45 (Cy 1*C*H, major + minor isomer); 120.3 (*C*H arom); 120.8 (*C*H arom); 120.9 (br, *C*H arom); 121.0 (Imdz *C*H, minor isomer); 121.0 (Imdz *C*H, major isomer); 122.3 (BPh_4 *p*-*C*H); 125.9 (br, *C*H arom); 126.1 (BPh_4 *m*-*C*H); 127.5 (*C*H arom); 130.0 (br, *C*H arom); 130.7 (*C*H arom); 130.9 (*C*H arom); 135.8 (NH-Ph *ipso*-C_q, major isom.); 136.3 (N-Ph *ipso*-C_q, major+minor isom.); 134.5 (BPh_4 *o*-C*H*); 136.8 (NH-Ph *ipso*-C_q, minor isom.); 139.3 (*C*(NPh)NHPh major+minor isom.); 145.9 (Imdz C_q); 154.4 (m, BPh_4 *ipso*-C_q). IR (nujol mull, cm^{-1}): 3325 (st, υ N-H); 1629, 1577 (st, υ C=N). ESI-ES (from methanol): m/z 427.4 ($[M+H]^+$). Elemental Analysis Calcd for $C_{42}H_{37}BN_4$: C, 83.40; H 7.67; N, 7.48; Found, C, 83.40; H, 7.51; N, 7.29.

Synthesis of $[H{\cdot}ICy^{\cdot(Ph)}NC^{15}N]^+{\cdot}[BPh4]^-$: The ^{15}N-labeled salt was prepared similarly in 70 % crude yield (58.1 %, recrystallized) starting from $ICy^{\cdot(Ph)}NC^{15}N$. The IR spectrum (nujol) and solution ^1H and $^{13}C\{^1H\}$ NMR spectra showed no significant differences with the non-labeled compound, except for the slight broadening of the quaternary *ipso*-C atoms of the Ph groups directly bound to the "amidine" N atoms due to unresolved ^{15}N-^{13}C coupling. The signal of the *C*(NPh)NHPh is clearly split in a multiplet (overlapping singlet, doublet and triplet signals for each of the isotopologues with $^1J_{NC}$= 13 Hz). CP-MAS ^{15}N NMR: δ 260 (low intensity, =N-Ph); 120 (higher intensity, -NHPh). The ESI-MS spectrum (from methanol) showed a signal for the ^{15}N-labeled molecular ion with the isotope pattern described previously for $ICy^{\cdot(Ph)}NC^{15}N$.

Synthesis of Pt NPs.

$Pt/ICy^{\cdot(p\text{-}tol)}NCN_{0,1}$: A 100 ml Fischer–Porter bottle provided with a stirring bar was charged with $Pt(NBE)_3$ (100 mg, 0.21 mmol) and 30 ml of THF, previously degassed by three freeze-pump cycles. The resulting solution was cooled at -80°C and a THF solution of 30 ml containing 0.1 equiv. of $ICy^{\cdot(p\text{-}tol)}NCN$ ligand (9.54 mg, 0.021 mmol) was added. The Fischer-Porter was then pressurized with 3 bar of H_2, and the solution was allowed to reach the room temperature under vigorous stirring. A black homogeneous solution was immediately formed. The stirring was continued for 2 h at r.t. After that period of time, the remaining H_2 pressure was released and the solution was concentrated to 2-3 mL. After adding 50 ml of pentane a resulting black precipitate was obtained and it was washed twice with pentane (50 mL) and dried overnight under vacuum. The size of the NPs was measured by TEM on a sample of at least 100 nanoparticles, which afforded a mean value of 2.3 (0.3) nm. $[ICy^{\cdot(p\text{-}tol)}NCN]/[Pt]$ = 0.1. Elemental analysis and ICP gave the following composition. Calcd: Pt, 81.20; C, 15.05; H 1.59; N, 2.34. Found, Pt, 80.11; C, 7.34; H 0.79; N, 1.13.

Pt/ICy·$^{(p\text{-}tol)}$NCN$_{0,2}$: These Pt NPs were prepared as described for Pt/ICy·$^{(p\text{-tol})}$NCN$_{0,1}$. [Pt(NBE)$_3$] (100 mg, 0.21 mmol), ICy·$^{(p\text{-tol})}$NCN (19.08 mg, 0.042 mmol). The size of the NPs was measured by TEM on a sample of at least 100 nanoparticles, which afforded a mean value of 2.1 (0.2) nm. [ICy·$^{(p\text{-tol})}$NCN]/[Pt] = 0.2. Elemental analysis and ICP gave the following composition. Calcd: Pt, 68.21; C, 25.19; H 2.67; N, 3.93. Found, Pt, 63.16; C, 10.60; H 1.13; N, 1.62.

Pt/ICy·$^{(p\text{-}tol)}$NCN$_{0,5}$: These Pt NPs were prepared as described for Pt/ICy·$^{(p\text{-tol})}$NCN$_{0,1}$. [Pt(NBE)$_3$] (100 mg, 0.21 mmol), ICy·$^{(p\text{-tol})}$NCN (47.7 mg, 0.105 mmol). The size of the NPs was measured by TEM on a sample of at least 100 nanoparticles, which afforded a mean value of 1.9 (0.4) nm. [ICy·$^{(p\text{-tol})}$NCN]/[Pt] = 0.5. Elemental analysis and ICP gave the following composition. Calcd: Pt, 46.72; C, 42.21; H 5.51; N, 6.50. Found, Pt, 48.14; C, 29.90; H 3.19; N, 4.57.

Pt/ICy·$^{(p\text{-}anisyl)}$NCN$_{0,2}$: These Pt NPs were prepared as described for Pt/ICy·$^{(p\text{-tol})}$NCN$_{0,1}$. [Pt(NBE)$_3$] (100 mg, 0.21 mmol), ICy·$^{(p\text{-anisyl})}$NCN (20.43 mg, 0.042 mmol). The size of the NPs was measured by TEM on a sample of at least 100 nanoparticles, which afforded a mean value of 2.0 (0.3) nm.
Pt/ICy·$^{(p\text{-}ClC6H4)}$NCN$_{0,2}$: These Pt NPs were prepared as described for Pt/ICy·$^{(p\text{-tol})}$NCN$_{0,1}$. [Pt(NBE)$_3$] (100 mg, 0.21 mmol), ICy·$^{(p\text{-ClC6H4})}$NCN (20.8 mg, 0.042 mmol). The size of the NPs was measured by TEM on a sample of at least 100 nanoparticles, which afforded a mean value of 1.9 (0.4) nm.

Pt/ICy·$^{(Ph)}$NC^{15}N$_{0,5}$: These Pt NPs were prepared as described for Pt/ICy·$^{(p\text{-tol})}$NCN$_{0,1}$. [Pt(NBE)$_3$] (100 mg, 0.21 mmol), ICy·$^{(Ph)}$NC^{15}N (44.9 mg, 0.105 mmol). The size of the NPs was measured by TEM on a sample of at least 100 nanoparticles, which afforded a mean value of 1.9 (0.3) nm.

Pt/CO/ICy·$^{(p\text{-}tol)}$NCN$_{0,2}$: A solution of $Pt_2(DBA)_3$ (180 mg, 0.16 mmol, 0.32 mmol Pt) in 40 mL of freshly distilled and degassed THF in a Fischer–Porter bottle was pressurized with 1 bar of CO for 30 min under vigorous stirring. During this time the solution changed from deep purple to brown. The solution was further removed by filtration and transferred into a schlenck tube for solvent evaporation under vacuum. The brown precipitate was then washed with pentane (3 × 35 ml) until the solution turned colorless to eliminate dba and dried under vacuum, giving then rise to $Pt_x(CO)_y(THF)_z$. A solution of ICy·$^{(p\text{-tol})}$NCN (29.01 mg, 0.064 mmol) in 10 mL THF was added into a dispersion of $Pt_x(CO)_y(THF)_z$ in 20 mL THF. The reaction mixture was stirred vigorously overnight and after that the solution was concentrated to 2-3 mL. After adding 30 ml of pentane, a resulting black precipitate was obtained and it was washed twice with pentane (20 mL) and dried overnight under vacuum. The size of the NPs was measured by TEM on a sample of at least 100 nanoparticles, which afforded a mean value of 1.2 (0.3) nm.

Pt/CO/ICy·$^{(Ph)}$NC^{15}N$_{0,2}$: These Pt NPs were prepared as described for Pt/CO/ICy·$^{(p\text{-tol})}$NCN$_{0,2}$. [$Pt_2(DBA)_3$] (180 mg, 0.16 mmol, 0.32 mmol Pt), ICy·$^{(Ph)}$NC^{15}N (27.4 mg, 0.064 mmol). The size of the NPs was measured by TEM on a sample of at least 100 nanoparticles, which afforded a mean value of 1.2 (0.2) nm.

Synthesis of Ru NPs.

Ru/ICy·$^{(Ph)}$NC^{15}N$_{0,1}$: A 250 ml Fischer–Porter bottle provided with a stirring bar was charged with Ru(COD)(COT) (150 mg, 0.48 mmol) and 75 ml of THF, previously degassed by three freeze-pump cycles. The resulting yellow solution was cooled at -80°C and a THF solution of (75 ml) containing 0.1 equiv. of ICy·$^{(Ph)}$NC^{15}N ligand (20.52 mg, 0.048 mmol) was added. The Fischer-Porter was then pressurized with 3 bar of H_2, and the solution was allowed to reach the r.t. under vigourous stirring. A black homogeneous solution was immediately formed. The stirring was continued for 20 h at r.t. After that period of time, the remaining H_2 pressure was released, the solution was transferred to a Schlenk tube and concentrated to 10 ml before adding 50 ml of pentane. The resulting black precipitate was washed twice with pentane (50 mL) and dried overnight under vacuum. The size of the NPs was measured by TEM on a sample of at least 200 nanoparticles, which afforded a mean value of 1.3 (0.3) nm. [ICy·$^{(Ph)}$NC^{15}N]/[Ru] = 0.1.

Ru/ICy·$^{(Ph)}$NC^{15}N$_{0,2}$: These Ru NPs were prepared as described for Ru/ICy·$^{(Ph)}$NC^{15}N$_{0,1}$. [Ru(COD)(COT)] (150 mg, 0.48 mmol), ICy·$^{(Ph)}$NC^{15}N (41.04 mg, 0.096 mmol). The size of the NPs was measured by TEM on a sample of at least 100 nanoparticles, which afforded a mean value of 1.2 (0.2) nm. [ICy·$^{(Ph)}$NC^{15}N]/[Ru] = 0.2.

Ru/ICy·$^{(Ph)}$NC^{15}N$_{0,5}$: These Ru NPs were prepared as described for Ru/ICy·$^{(Ph)}$NC^{15}N$_{0,1}$. [Ru(COD)(COT)] (150 mg, 0.48 mmol), ICy·$^{(Ph)}$NC^{15}N (102.6 mg, 0.24 mmol). The size of the NPs was measured by TEM on a sample of at least 100 nanoparticles, which afforded a mean value of 1.1 (0.1) nm. [ICy·$^{(Ph)}$NC^{15}N]/[Ru] = 0.5.

Ru/ICy·$^{(Ph)}$NC^{15}N$_{1}$: These Ru NPs were prepared as described for Ru/ICy·$^{(Ph)}$NC^{15}N$_{0,1}$. [Ru(COD)(COT)] (150 mg, 0.48 mmol), ICy·$^{(Ph)}$NC^{15}N (205.2 mg, 0.48 mmol). The size of the NPs was measured by TEM on a sample of at least 100 nanoparticles, which afforded a mean value of 1.0 (0.2) nm. [ICy·$^{(Ph)}$NC^{15}N]/[Ru] = 1.

Catalytic hydrogenation reactions.

Catalytic experiments were performed in a HEL 24-multireactor (volume of the tubes 1.5 mL). In a typical experiment, Pt NPs (0.0025 mmol of Pt assuming % of Pt from ICP analysis) in 0.75 mL of THF (as a standard solution, prepared immediately prior to use) was mixed with 0.5 mmol of substrate in 1.5 mL vials and the reactor was sealed under nitrogen before pressurization. The reactor was then pressurized with 3 bars of hydrogen and depressurized three times to purge and finally pressurized to 5 bars. The reactor was stirred during 20h at room temperature. After that, the reactor was slowly depressurized and samples from each reaction were passed through silica and analyzed by ^{1}H NMR.

Surfaces studies of Pt and Ru NPs through coordination of CO and ^{13}CO

For CO coordination studies, metal NPs were introduced in a Fischer-Porter bottle and were pressurized with 3 bar of H_2 for 20 h to avoid the presence of oxygen on the surface. After this period of time, the dihydrogen gas was evacuated under vacuum for 15 min. The Fischer-Porter bottle was further pressurized with 1 bar of CO/^{13}CO for 20 h. Then, the gas was evacuated under vacuum for 5 min. ^{13}C solid state NMR was recorded after transfer of the sample into a NMR rotor

FT-IR spectra were recorded from samples prepared in THF solutions after bubbling CO during 5 min.

TEM data

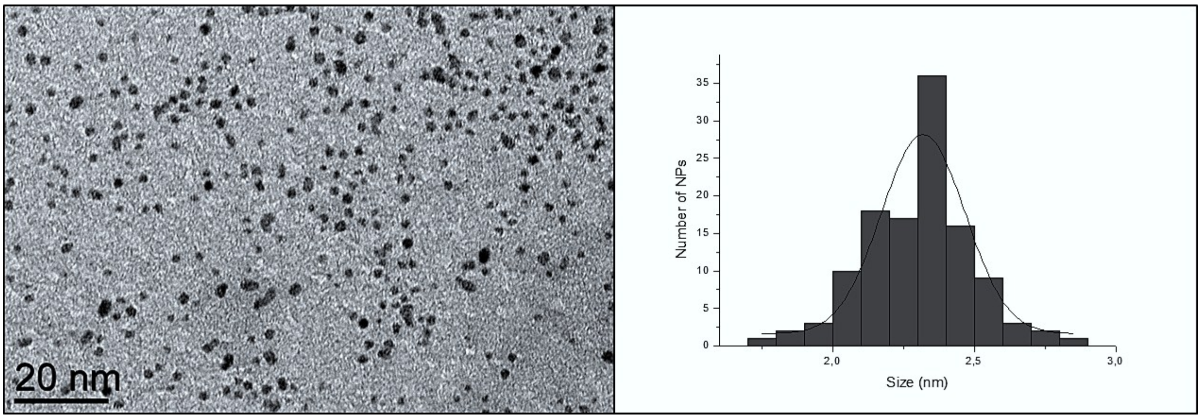

Figure S5. TEM micrographs of Pt/ICy$^{\cdot(p\text{-tol})}$NCN$_{0,1}$ (left) and the corresponding size histogram (right). Mean size = 2.3 (0.3) nm.

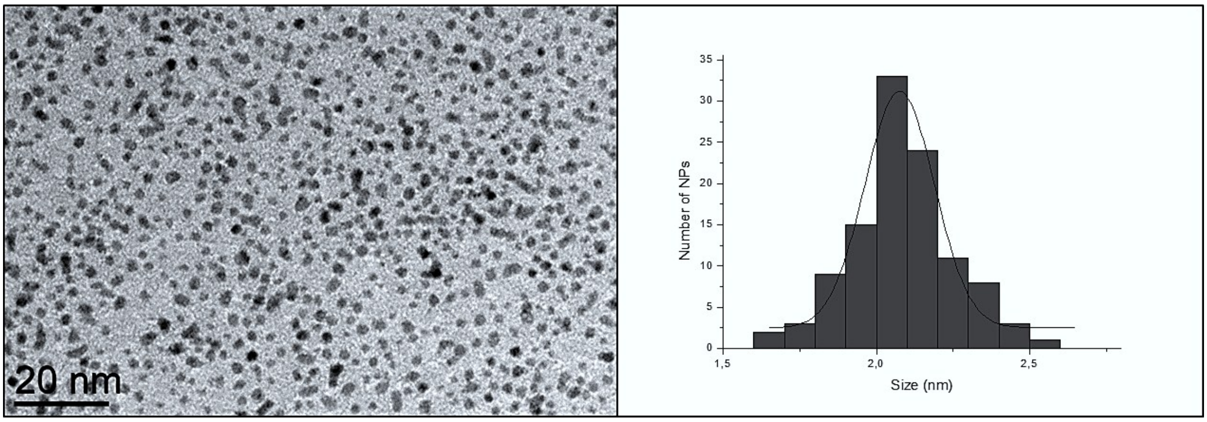

Figure S6. TEM micrographs of Pt/ICy$^{\cdot(p\text{-tol})}$NCN$_{0,2}$ (left) and the corresponding size histogram (right). Mean size = 2.1 (0.2) nm.

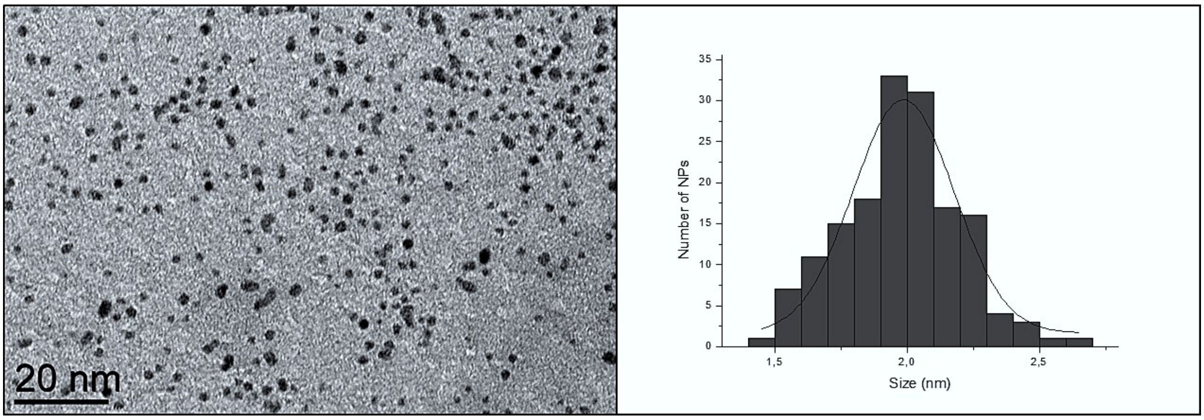

Figure S7. TEM micrographs of Pt/ICy$^{\cdot(p\text{-tol})}$NCN$_{0,5}$ (left) and the corresponding size histogram (right). Mean size = 1.9 (0.4) nm.

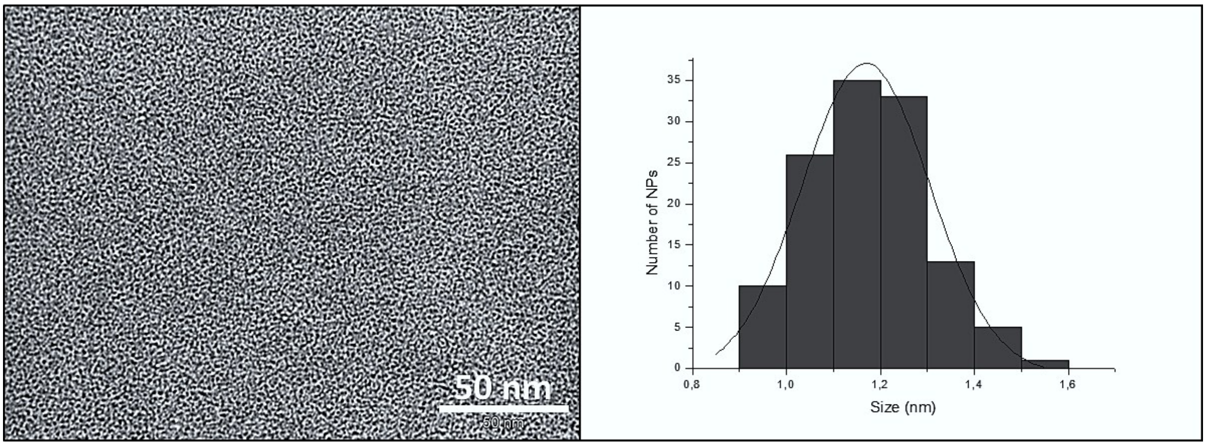

Figure S8. TEM micrographs of $Pt/CO/ICy^{\cdot(p\text{-tol})}NCN_{0,2}$ (left) and the corresponding size histogram (right). Mean size = 1.2 (0.3) nm.

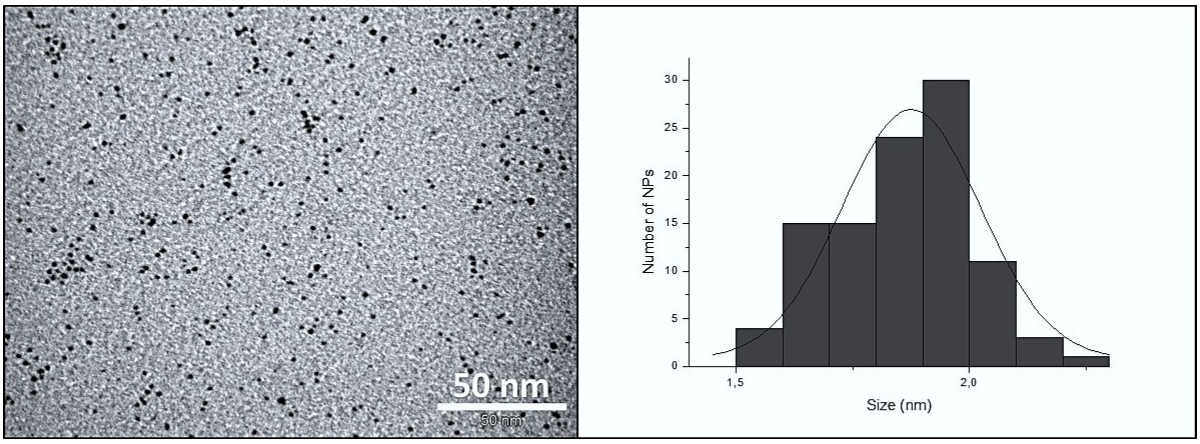

Figure S9. TEM micrographs of $Pt/ICy^{\cdot(Ph)}NC^{15}N_{0,5}$ (left) and the corresponding size histogram (right). Mean size = 1.9 (0.3) nm.

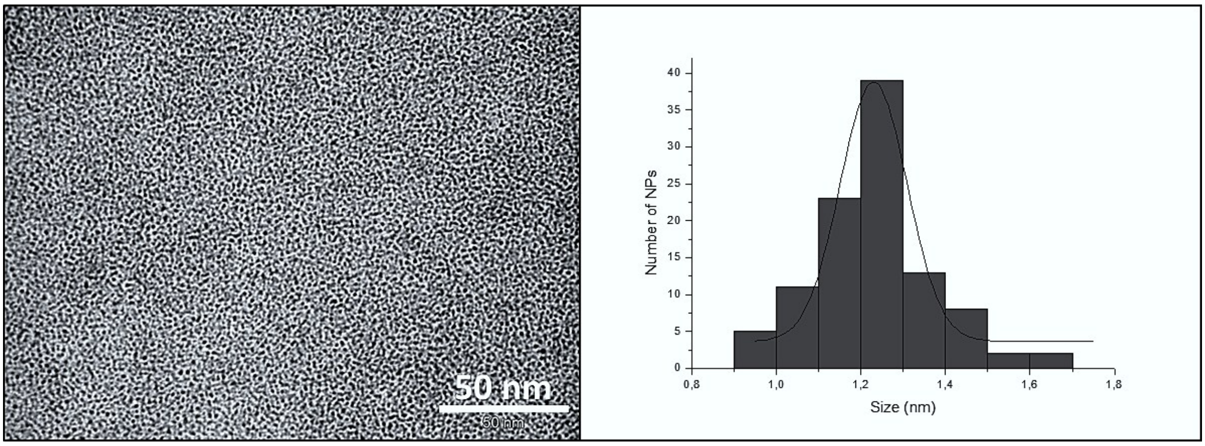

Figure S10. TEM micrographs of $Pt/CO/ICy^{\cdot(Ph)}NC^{15}N_{0,2}$ (left) and the corresponding size histogram (right). Mean size = 1.2 (0.2) nm.

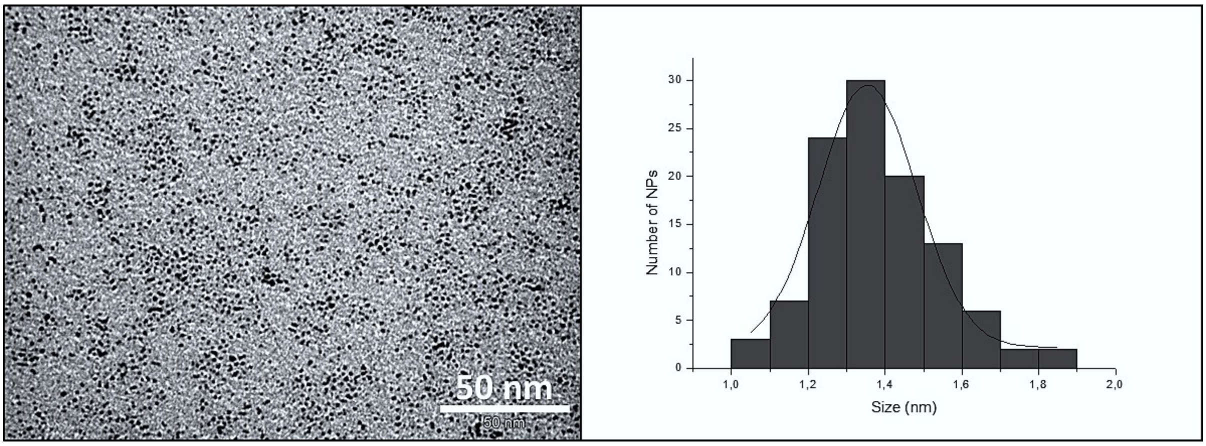

Figure S11. TEM micrographs of $Ru/ICy^{\cdot(Ph)}NC^{15}N_{0.1}$ (left) and the corresponding size histogram (right). Mean size = 1.3 (0.3) nm.

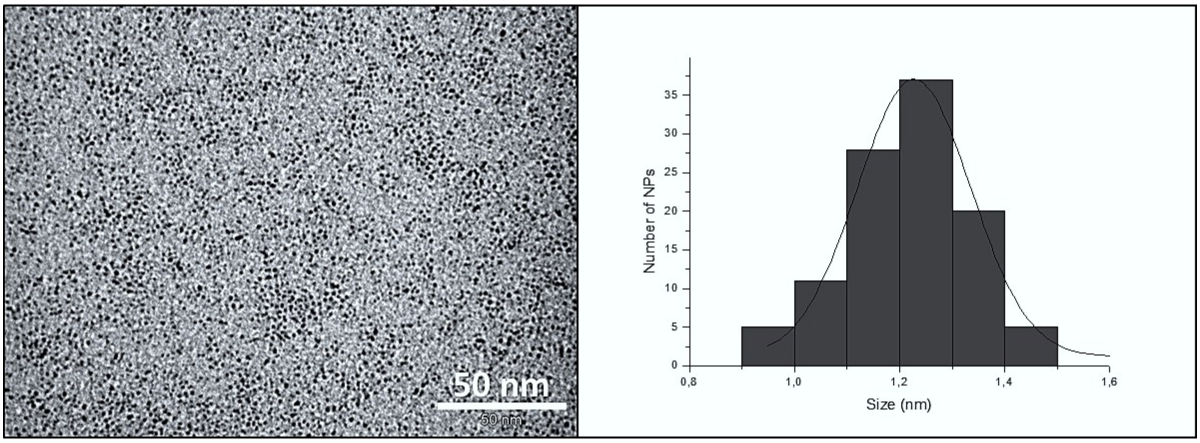

Figure S12. TEM micrographs of $Ru/ICy^{\cdot(Ph)}NC^{15}N_{0.2}$ (left) and the corresponding size histogram (right). Mean size = 1.2 (0.2) nm.

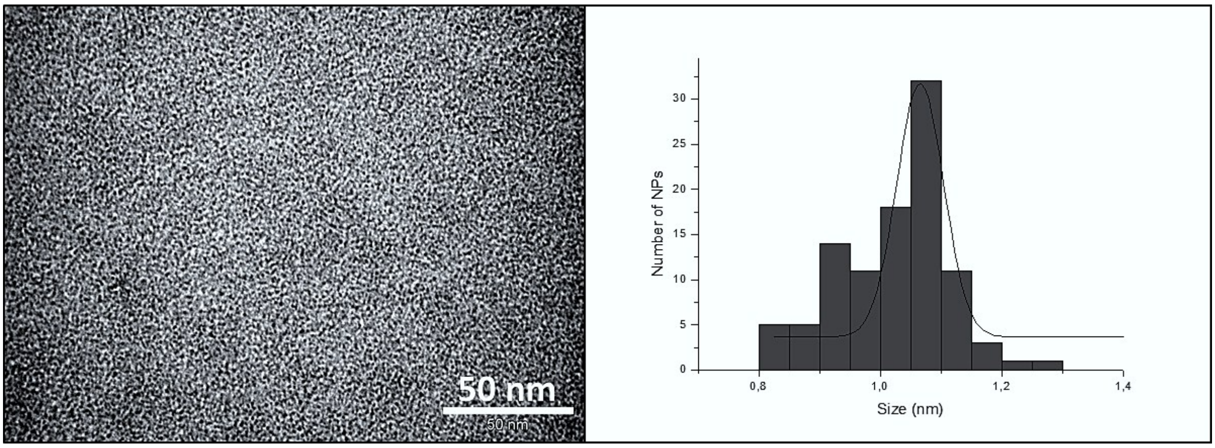

Figure S13. TEM micrographs of $Ru/ICy^{\cdot(Ph)}NC^{15}N_{0.5}$ (left) and the corresponding size histogram (right). Mean size = 1.1 (0.1) nm.

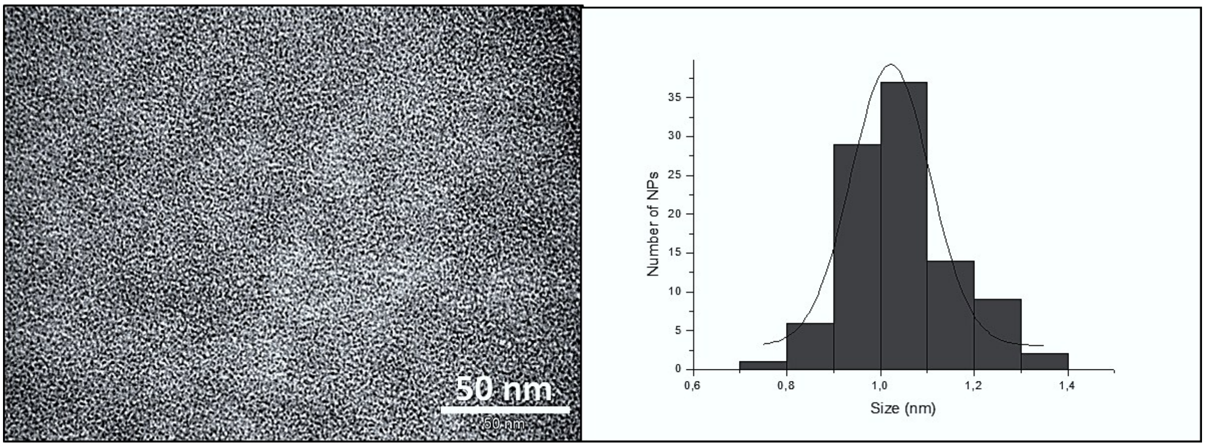

Figure S14. TEM micrographs of Ru/ICy$^{\cdot(Ph)}$NC^{15}N$_1$ (left) and the corresponding size histogram (right). Mean size = 1.0 (0.2) nm.

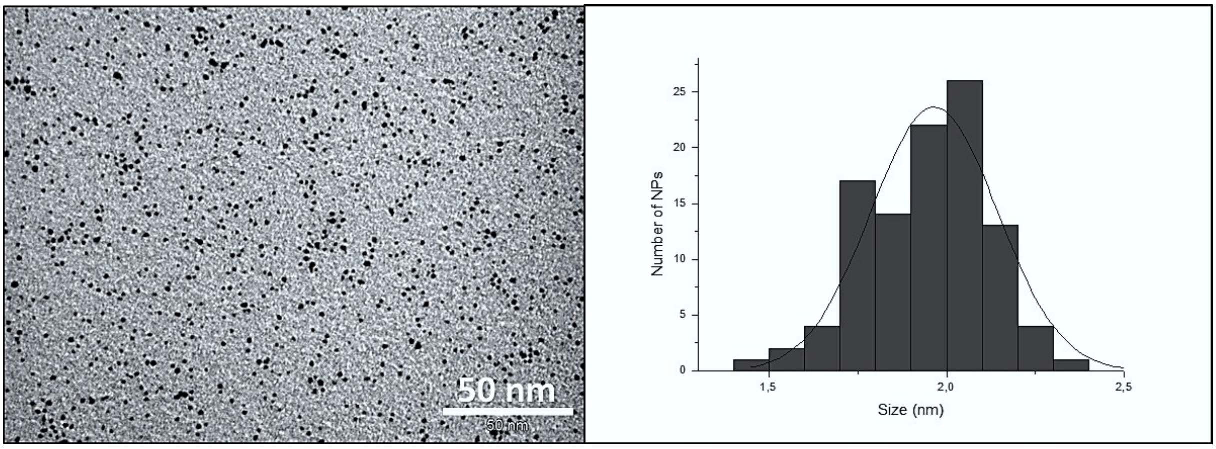

Figure S15. TEM micrographs of Pt/ICy$^{\cdot(p\text{-anisyl})}NCN_{0.2}$ (left) and the corresponding size histogram (right). Mean size = 2.0 (0.3) nm.

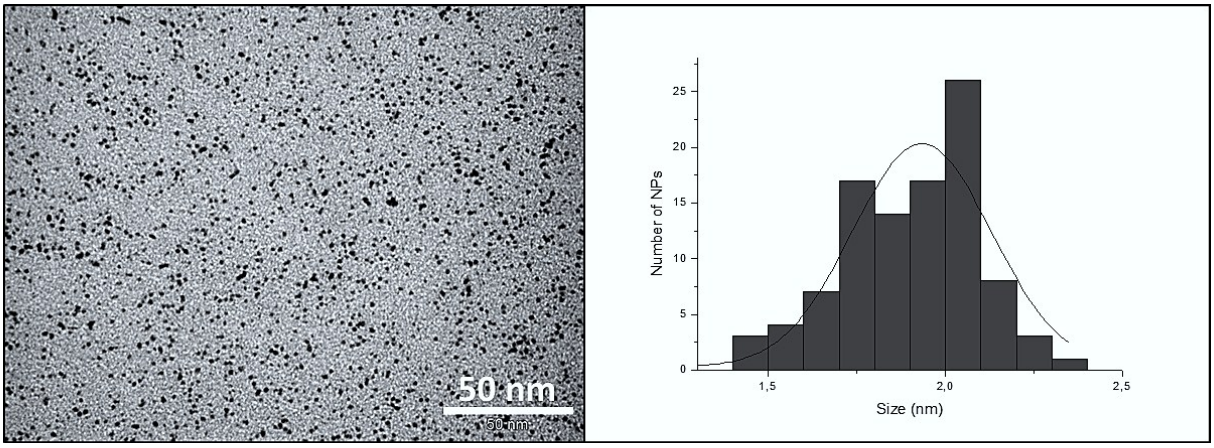

Figure S16. TEM micrographs of Pt/ICy$^{\cdot(p\text{-}ClC_6H_4)}NCN_{0.2}$ (left) and the corresponding size histogram (right). Mean size = 1.9 (0.4) nm.

WAXS data

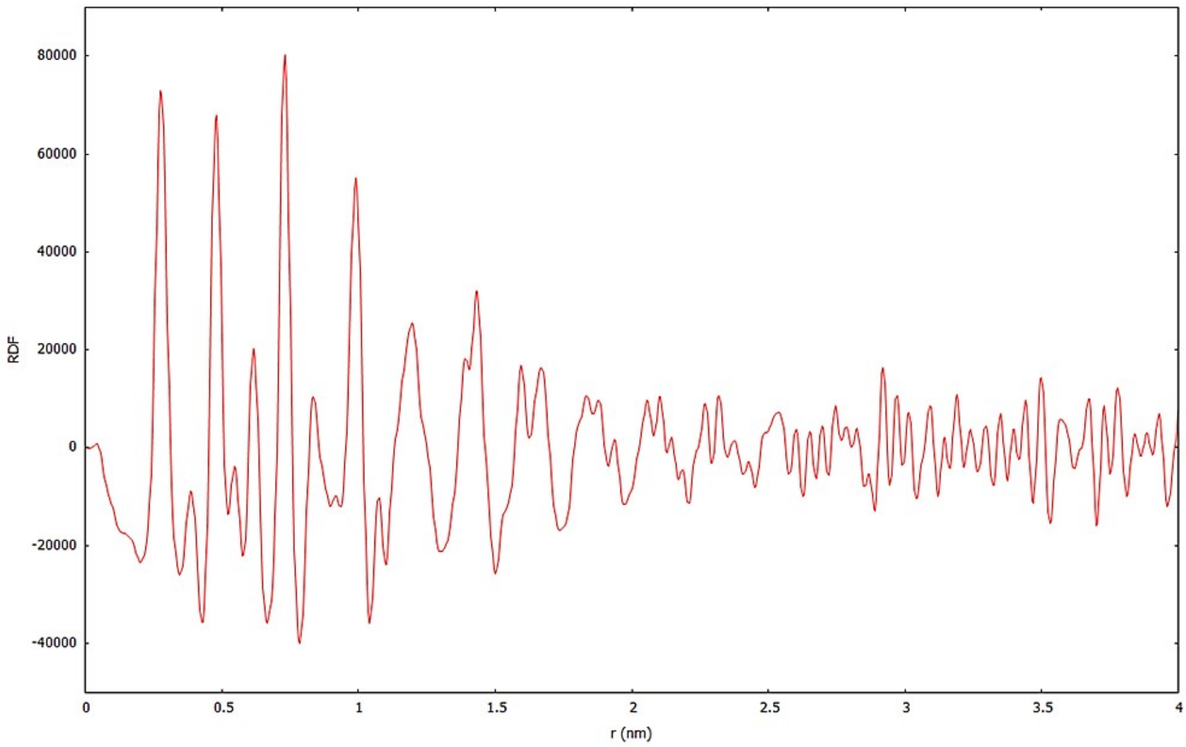

Figure S17. WAXS analysis of Pt/ICy$^{\cdot(p\text{-tol})}$NCN$_{0.1}$. Metallic Pt particles with a single domain. The pattern is consistent with *fcc* Pt and the coherence length is close to 2.5 nm.

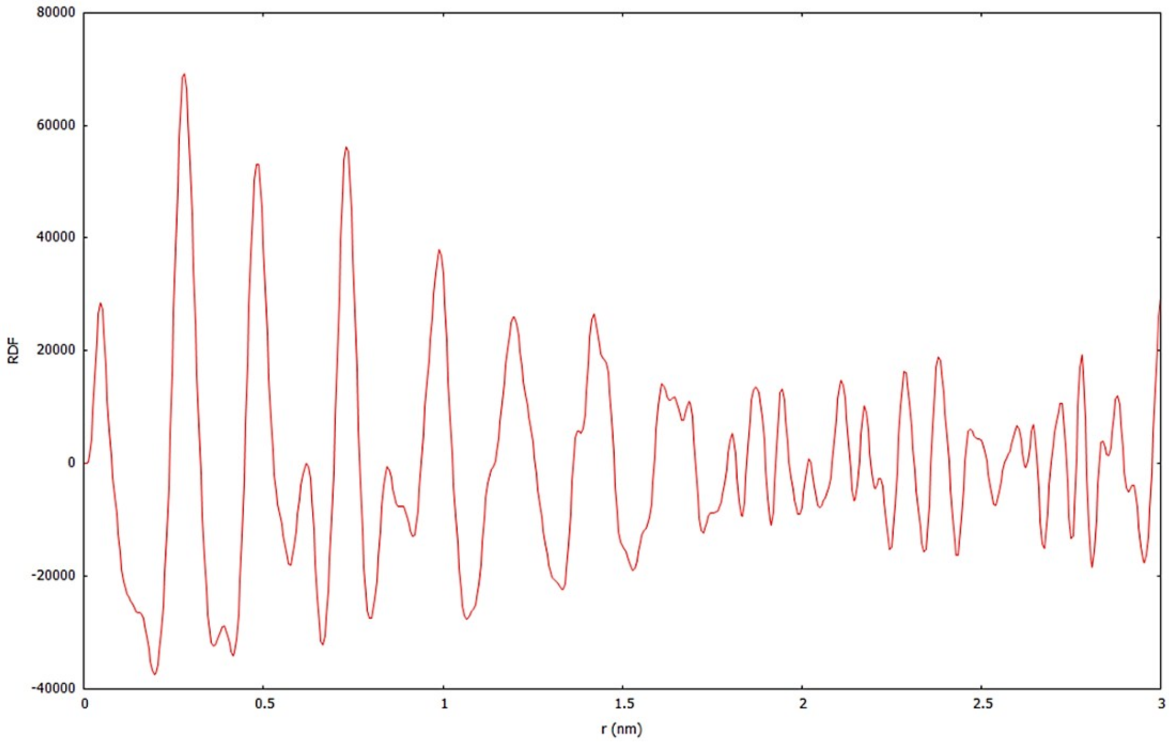

Figure S18. WAXS analysis of Pt/ICy$^{\cdot(p\text{-tol})}$NCN$_{0.2}$. Metallic Pt particles with a single domain. The pattern is consistent with *fcc* Pt and the coherence length is close to 2.2 nm.

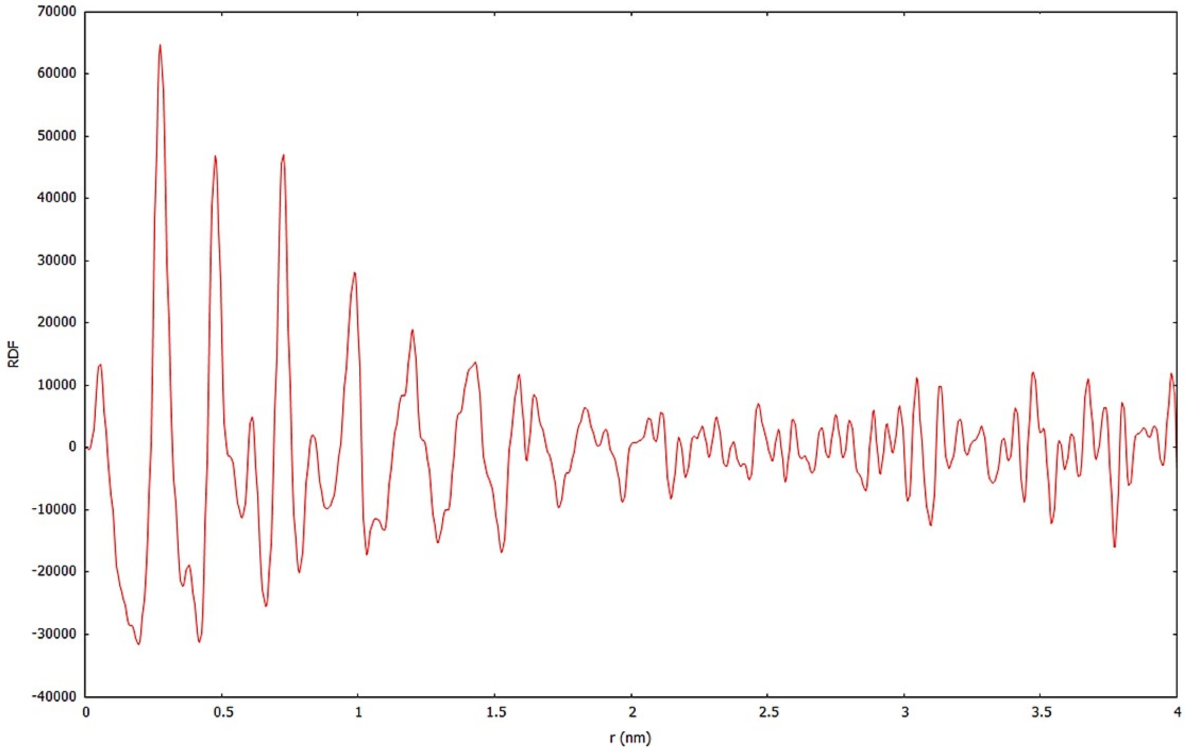

Figure S19. WAXS analysis of Pt/ICy$^{\cdot(p\text{-tol})}$NCN$_{0.5}$. Metallic Pt particles with a single domain. The pattern is consistent with *fcc* Pt and the coherence length is close to 2.1 nm.

FT-IR data

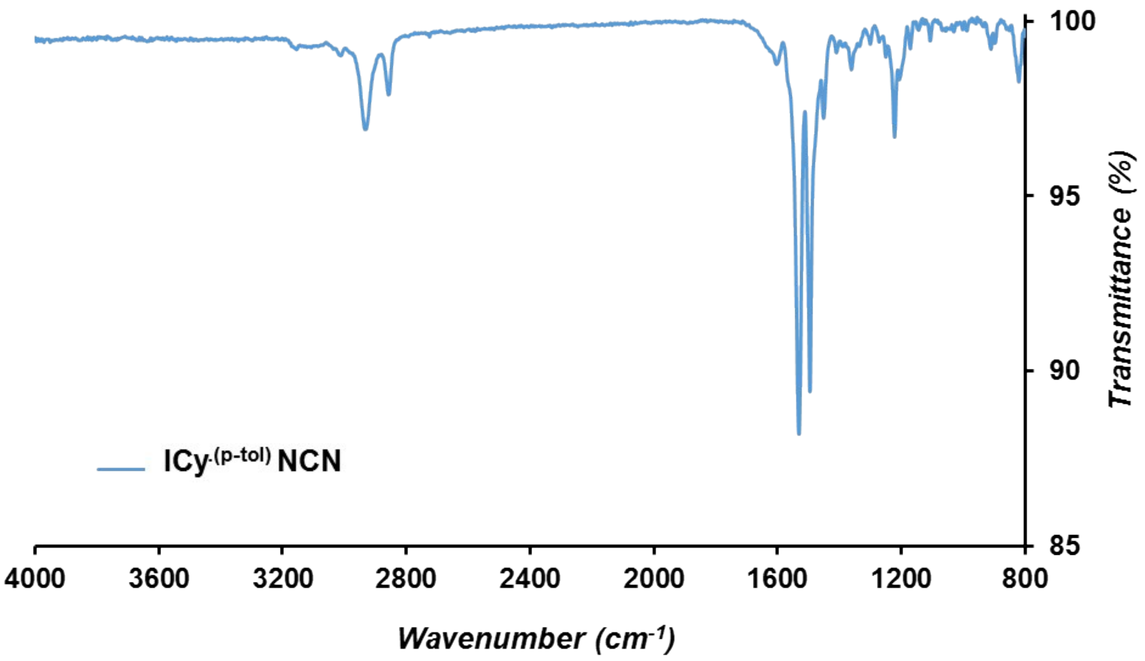

Figure S20. ATR FT-IR spectrum registered for ICy·$^{(p\text{-tol})}$NCN.

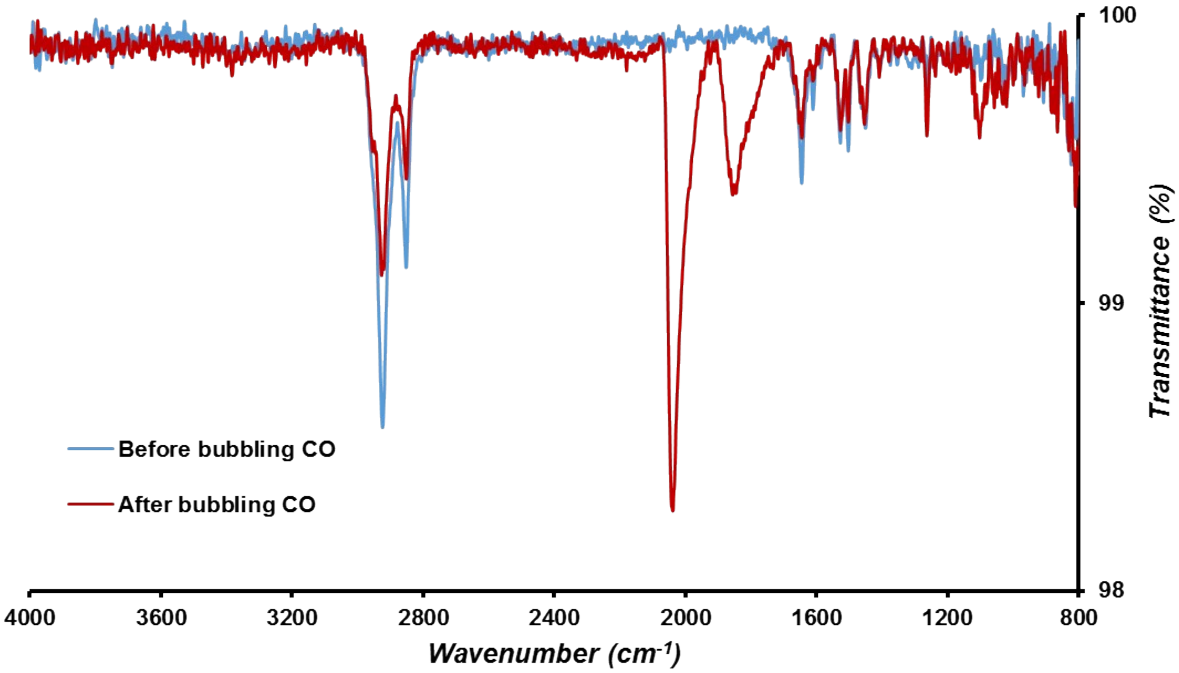

Figure S21. ATR FT-IR spectra registered for Pt/ICy·$^{(p\text{-tol})}$NCN$_{0.1}$ before (blue) and after (red) bubbling CO during 5 min.

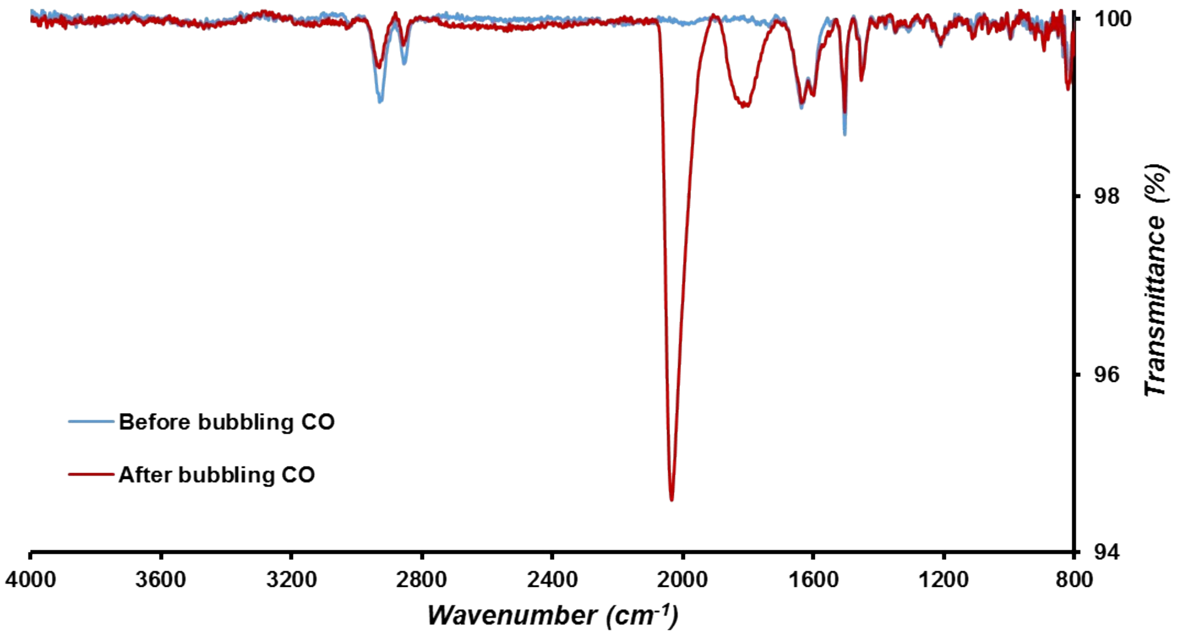

Figure S22. ATR FT-IR spectra registered for $Pt/ICy^{\cdot(p\text{-}tol)}NCN_{0.2}$ before (blue) and after (red) bubbling CO during 5 min.

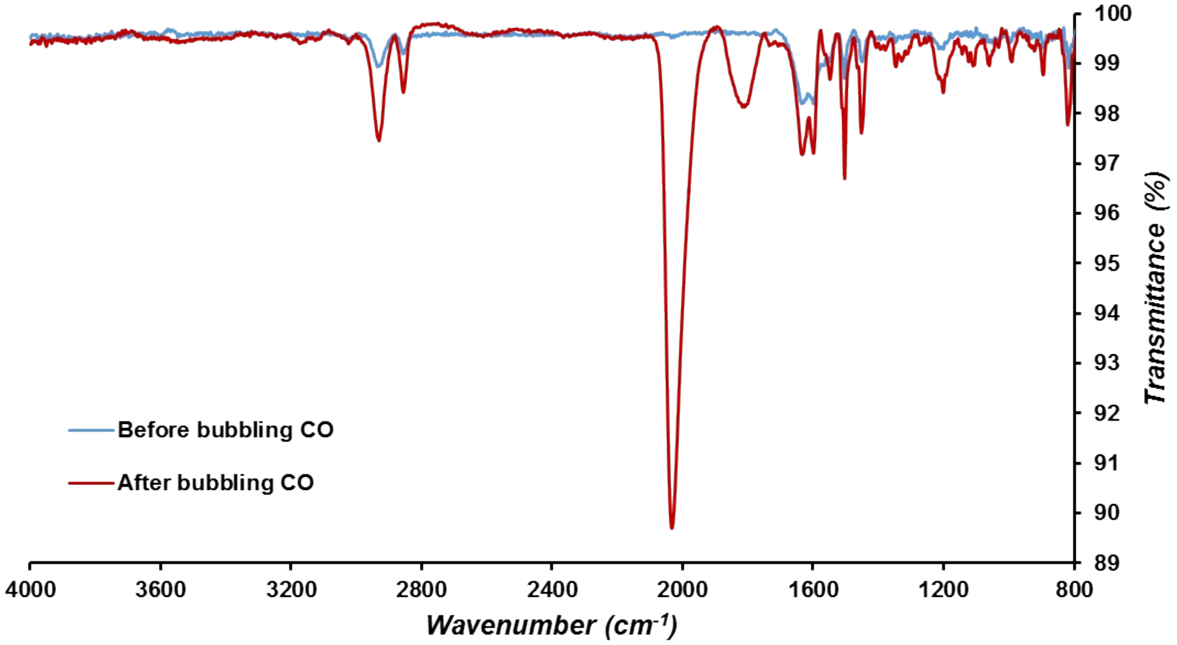

Figure S23. ATR FT-IR spectra registered for $Pt/ICy^{\cdot(p\text{-}tol)}NCN_{0.5}$ before (blue) and after (red) bubbling CO during 5 min.

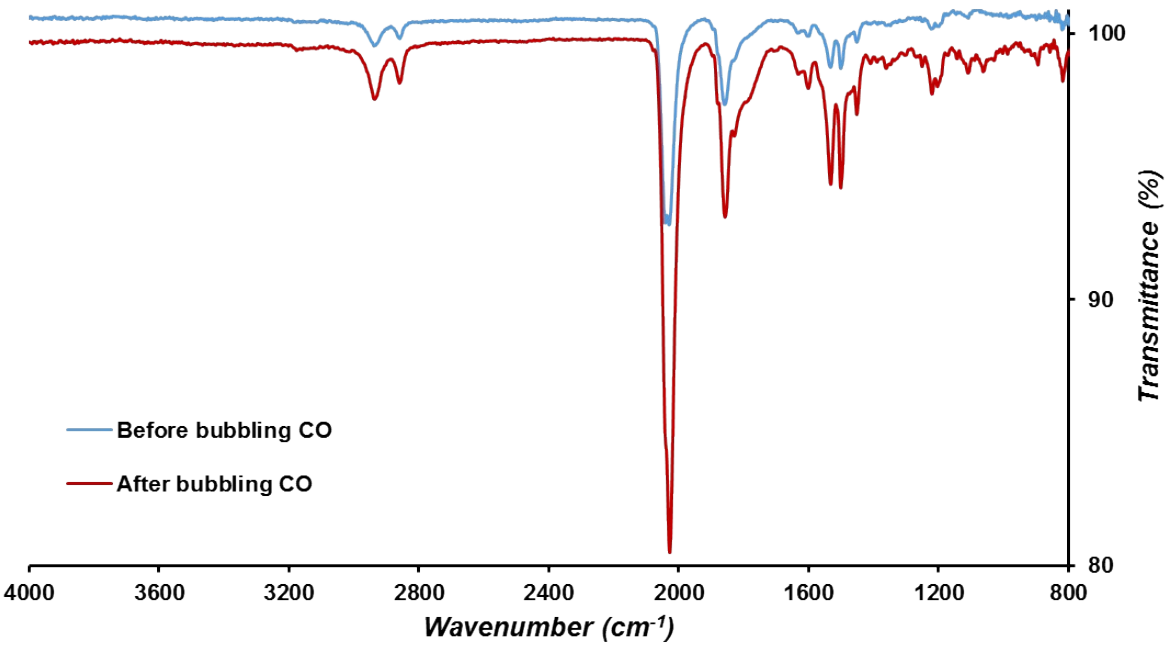

Figure S24. ATR FT-IR spectra registered for Pt/CO/ICy·(Ph)NC^{15}N$_{0.2}$ before (blue) and after (red) bubbling CO during 5 min.

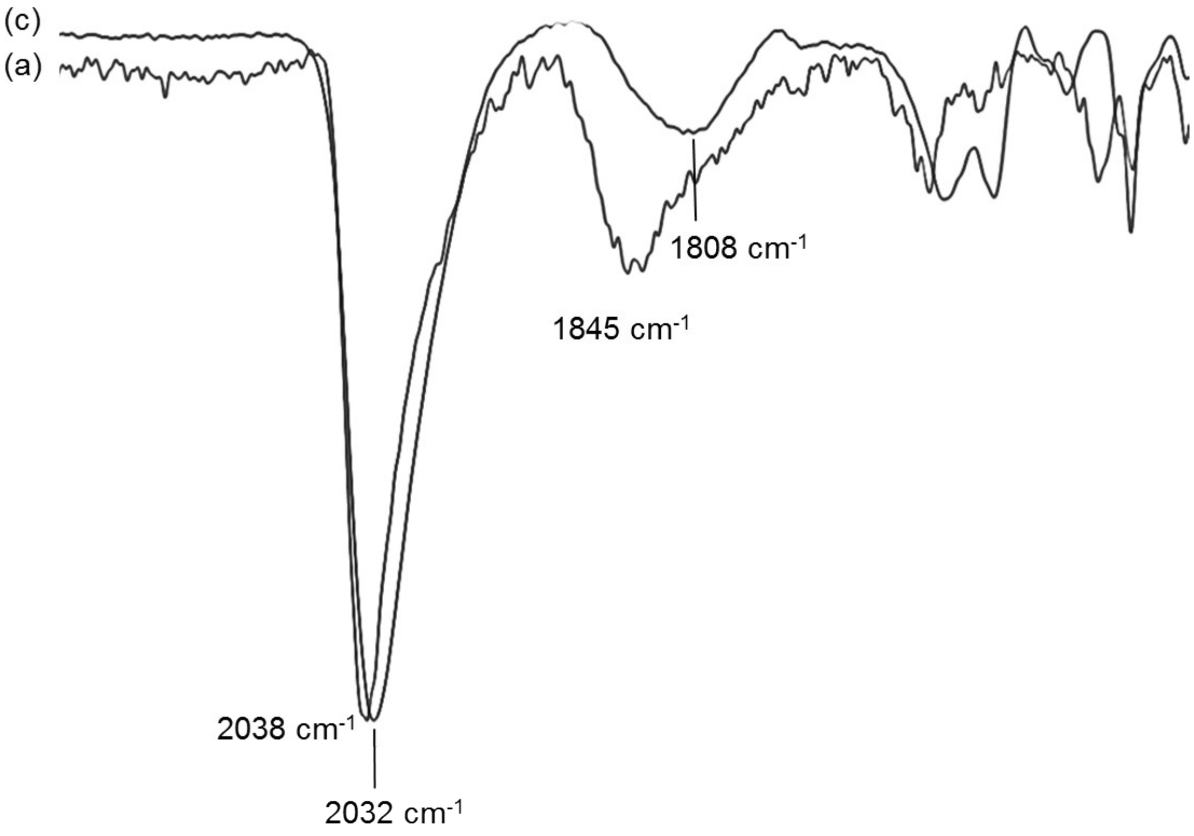

Figure S25. ATR FT-IR spectra of (a) Pt/ICy·(*p*-tol)NCN$_{0.1}$, (c) Pt/ICy·(*p*-tol)NCN$_{0.5}$.

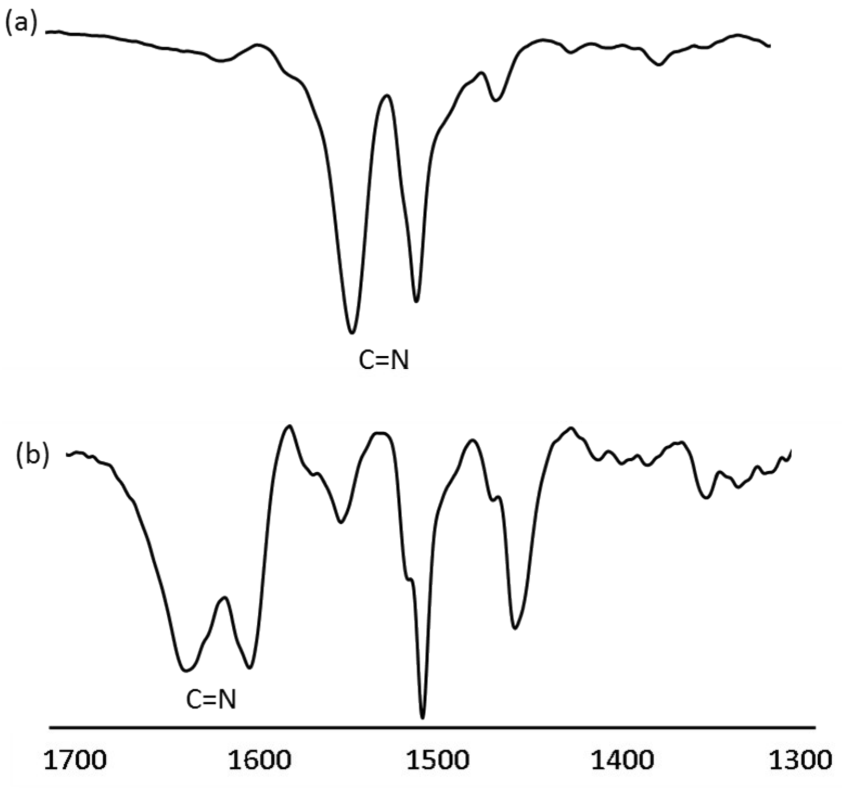

Figure S26. ATR FT-IR spectra of (a) ICy$^{\cdot(p\text{-tol})}$NCN and (c) Pt/ICy$^{\cdot(p\text{-tol})}$NCN$_{0.5}$.

Analytical data

Table S1. Analytical data of Pt/ICy$^{\cdot(p\text{-tol})}$NCN$_{0.1}$, Pt/ICy$^{\cdot(p\text{-tol})}$NCN$_{0.2}$ and Pt/ICy$^{\cdot(p\text{-tol})}$NCN$_{0.5}$.

NPs	Pt content (%)	C (%)	H (%)	N (%)	Pt : L Ratio
**Pt/ICy$^{\cdot(p\text{-tol})}$NCN$_{0.1}$**	90.1	7.34	0.79	1.13	1 : 0.04
**Pt/ICy$^{\cdot(p\text{-tol})}$NCN$_{0.2}$**	77.3	10.60	1.13	1.62	1 : 0.07
**Pt/ICy$^{\cdot(p\text{-tol})}$NCN$_{0.5}$**	61.0	29.90	3.19	4.57	1 : 0.26

Catalytical data

Table S2. Hydrogenation reactions catalyzed by Pt/ICy$^{\cdot(p\text{-tol})}$NCN$_{0.1}$, Pt/ICy$^{\cdot(p\text{-tol})}$NCN$_{0.2}$ and Pt/ICy$^{\cdot(p\text{-tol})}$NCN$_{0.5}$.[a]

Entry/ Ru-NP	Substrate	Products[b]	Conv. (%)[b] Select.[b]
**S1**/ Pt/ICy$^{\cdot(p\text{-tol})}$NCN$_{0.1}$	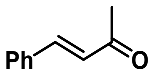 1	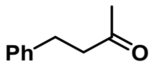 2 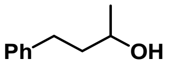 3	>99 **2/3**:77/23
**S2**/ Pt/ICy$^{\cdot(p\text{-tol})}$NCN$_{0.2}$			>99 **2/3**:81/19
**S3**/ Pt/ICy$^{\cdot(p\text{-tol})}$NCN$_{0.5}$			>99 **2/3**:81/19
**S4**/ Pt/ICy$^{\cdot(p\text{-tol})}$NCN$_{0.1}$	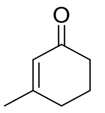 4	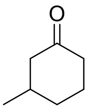 5 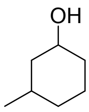 6	>99 **5/6**:56/44
**S5**/ Pt/ICy$^{\cdot(p\text{-tol})}$NCN$_{0.2}$			>99 **5/6**:68/32
**S6**/ Pt/ICy$^{\cdot(p\text{-tol})}$NCN$_{0.5}$			>99 **5/6**:71/29
**S7**/ Pt/ICy$^{\cdot(p\text{-tol})}$NCN$_{0.1}$	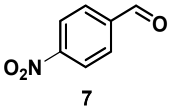 7	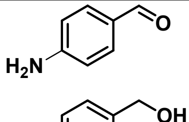 8 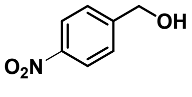 9 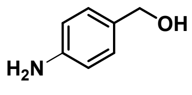 10	98 **8/9/10**:44/1/54
**S8**/ Pt/ICy$^{\cdot(p\text{-tol})}$NCN$_{0.2}$			98 **8/9/10**:53/1/44
**S9**/ Pt/ICy$^{\cdot(p\text{-tol})}$NCN$_{0.5}$			98 **8/9/10**:57/5/32

[a].Reaction conditions: Substrate (0.5 mmol), Ru-NPs (0.0025mmol of Pt assuming % of Pt from ICP analysis), THF (0.75 mL), 20 h, r.t., 5 bar.
[b] Conversions, selectivities and product identities were determined by ^{1}H NMR (average of two runs).

MAS NMR data

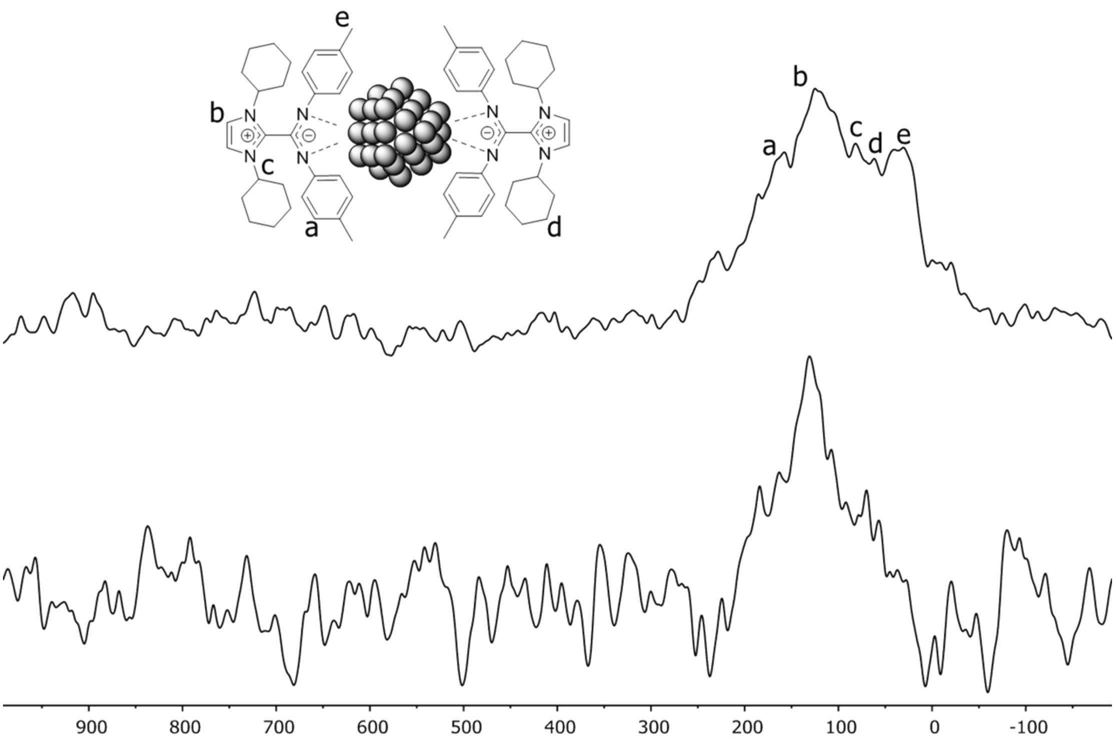

Figure S27. ^{13}C MAS (top) and CP-MAS (bottom) NMR spectra of Pt/ICy$^{\cdot(p\text{-tol})}$NCN$_{0.1}$.

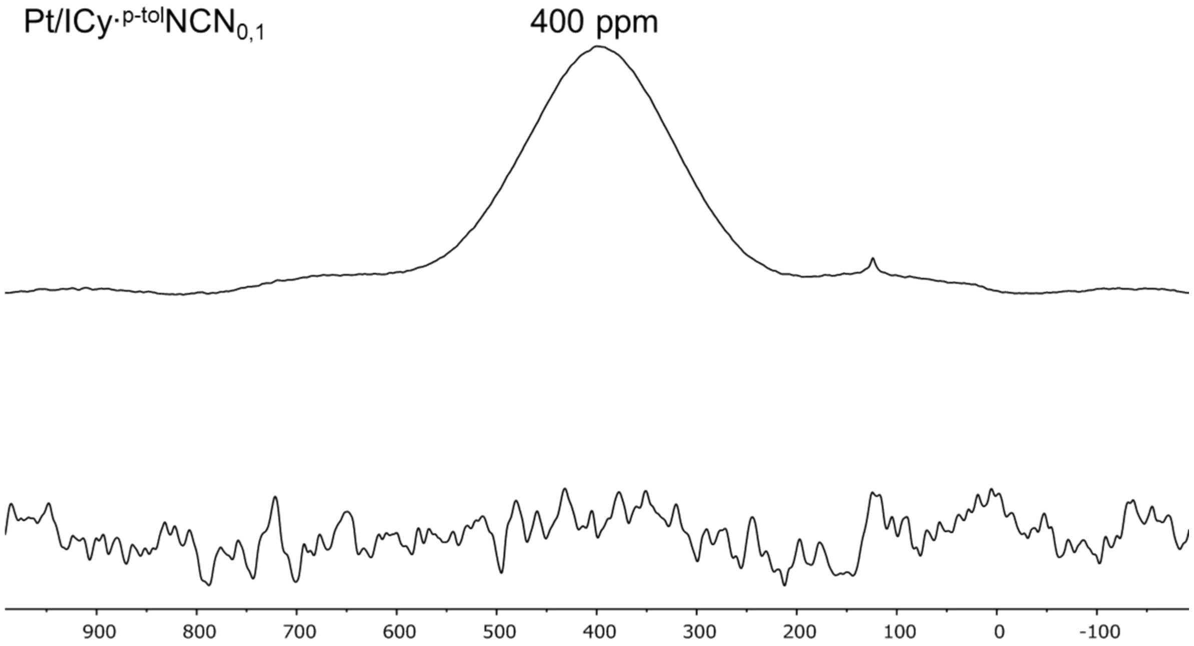

Figure S28. ^{13}C MAS (top) and CP-MAS (bottom) NMR spectra of Pt/ICy$^{\cdot(p\text{-tol})}$NCN$_{0.1}$ after exposure to ^{13}CO (1 bar, 20 h, at r.t.).

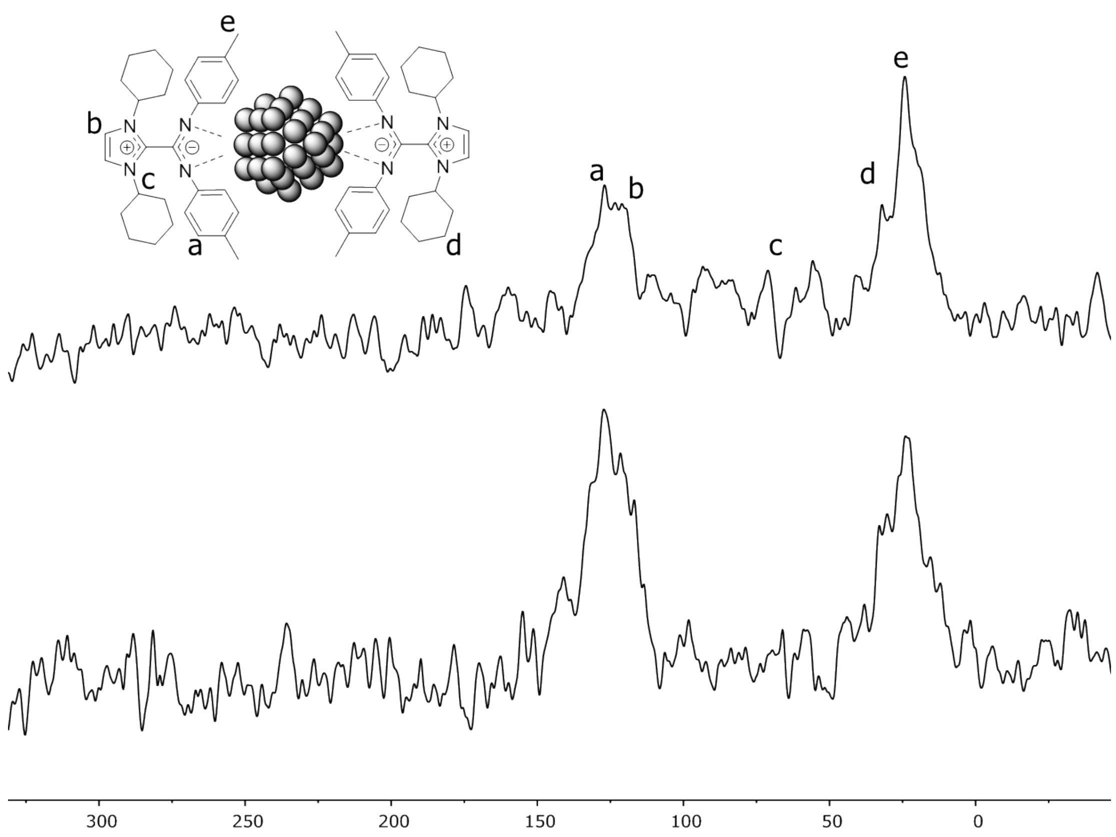

Figure S29. ^{13}C MAS (top) and CP-MAS (bottom) NMR spectra of Pt/ICy$^{\cdot(p\text{-tol})}$NCN$_{0.2}$.

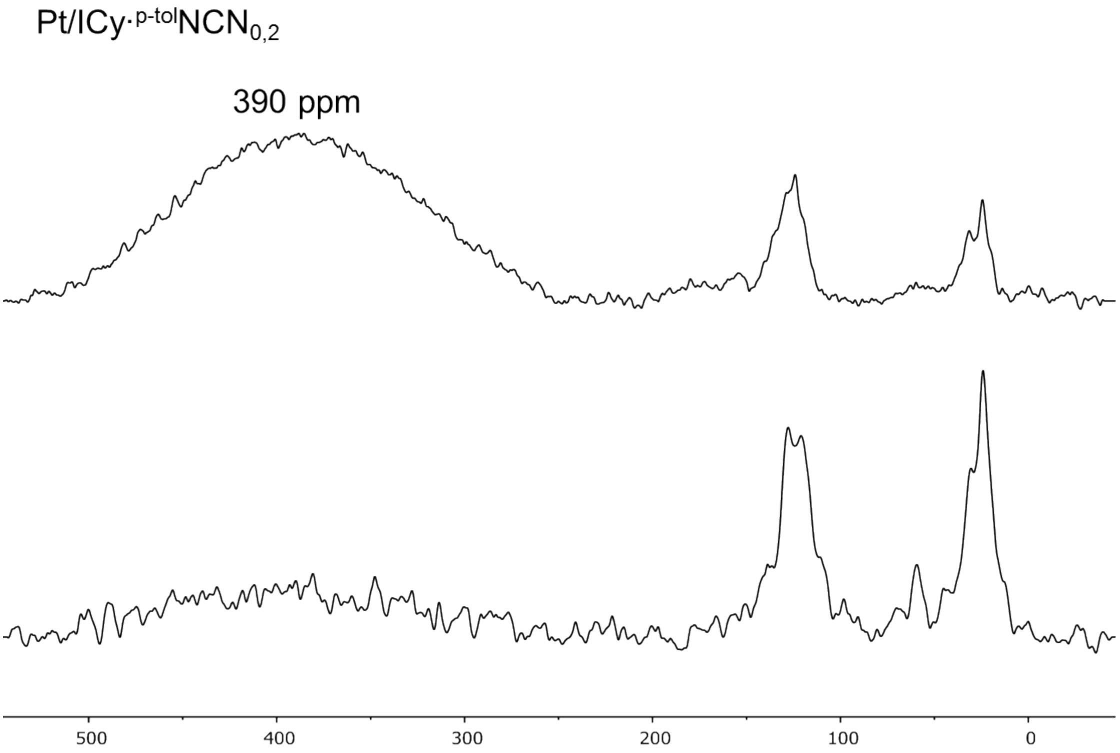

Figure S30. ^{13}C MAS (top) and CP-MAS (bottom) NMR spectra of Pt/ICy$^{\cdot(p\text{-tol})}$NCN$_{0.2}$ after exposure to ^{13}CO (1 bar, 20 h, at r.t.).

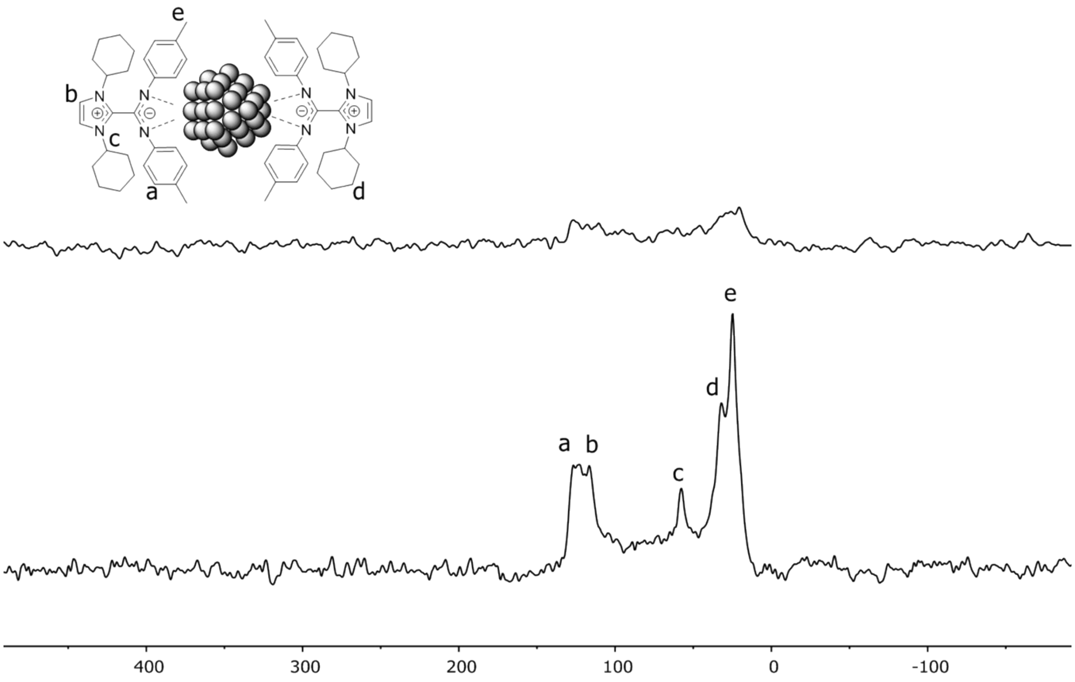

Figure S31. ^{13}C MAS (top) and CP-MAS (bottom) NMR spectra of Pt/ICy·$^{(p\text{-tol})}$NCN$_{0.5}$.

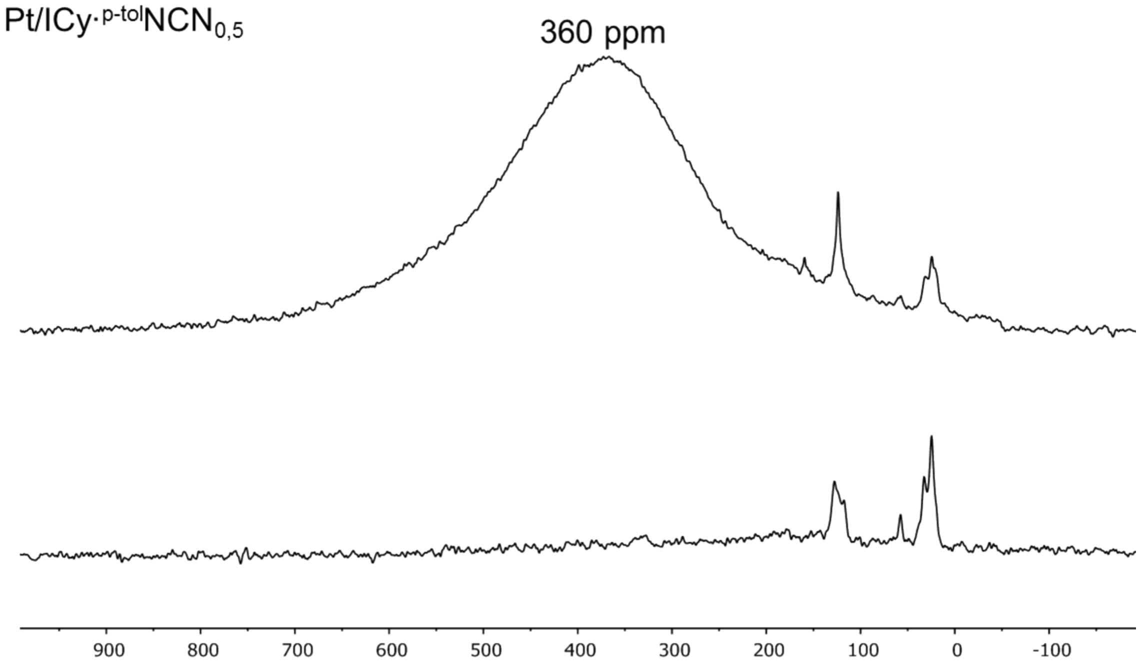

Figure S32. ^{13}C MAS (top) and CP-MAS (bottom) NMR spectra of Pt/ICy·$^{(p\text{-tol})}$NCN$_{0.5}$ after exposure to ^{13}CO (1 bar, 20 h, at r.t.).

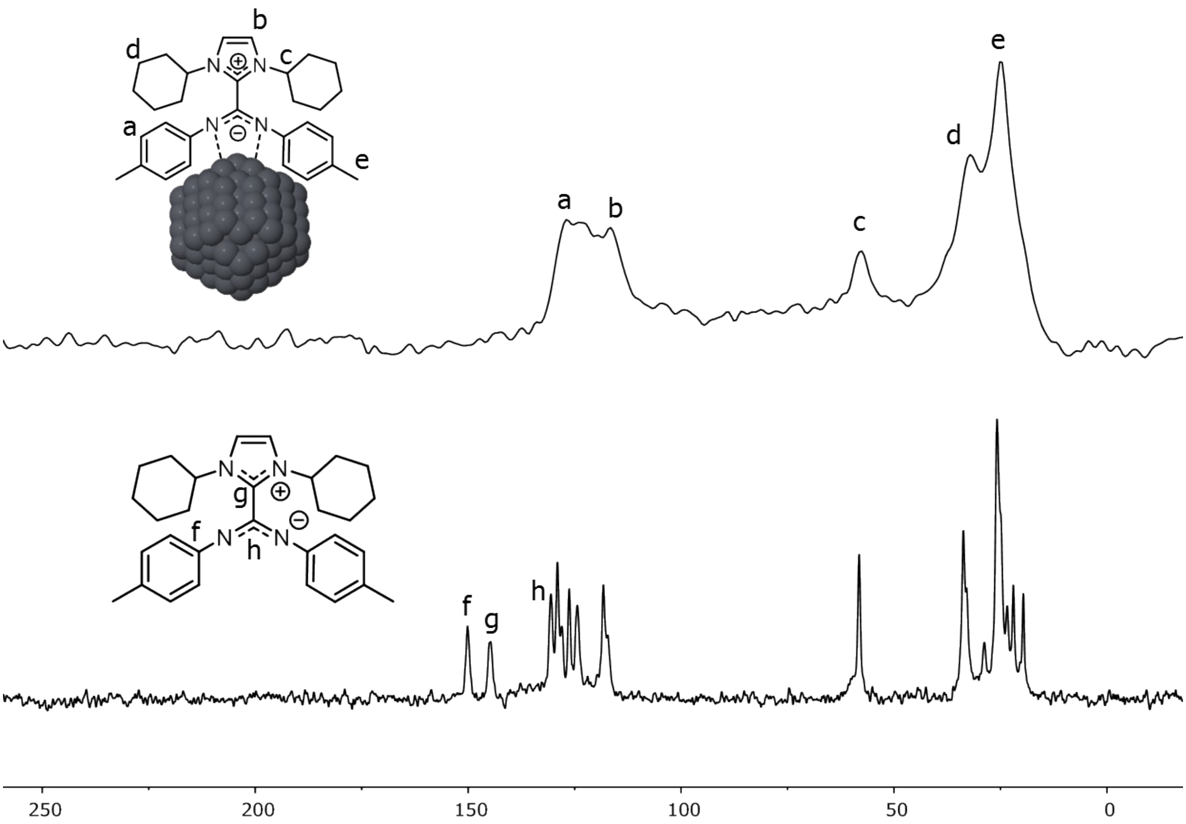

Figure S33. ^{13}C CP-MAS NMR spectra of ICy$^{\cdot(p\text{-tol})}$NCN (bottom) and Pt/ICy$^{\cdot(p\text{-tol})}$NCN$_{0.5}$ (top).

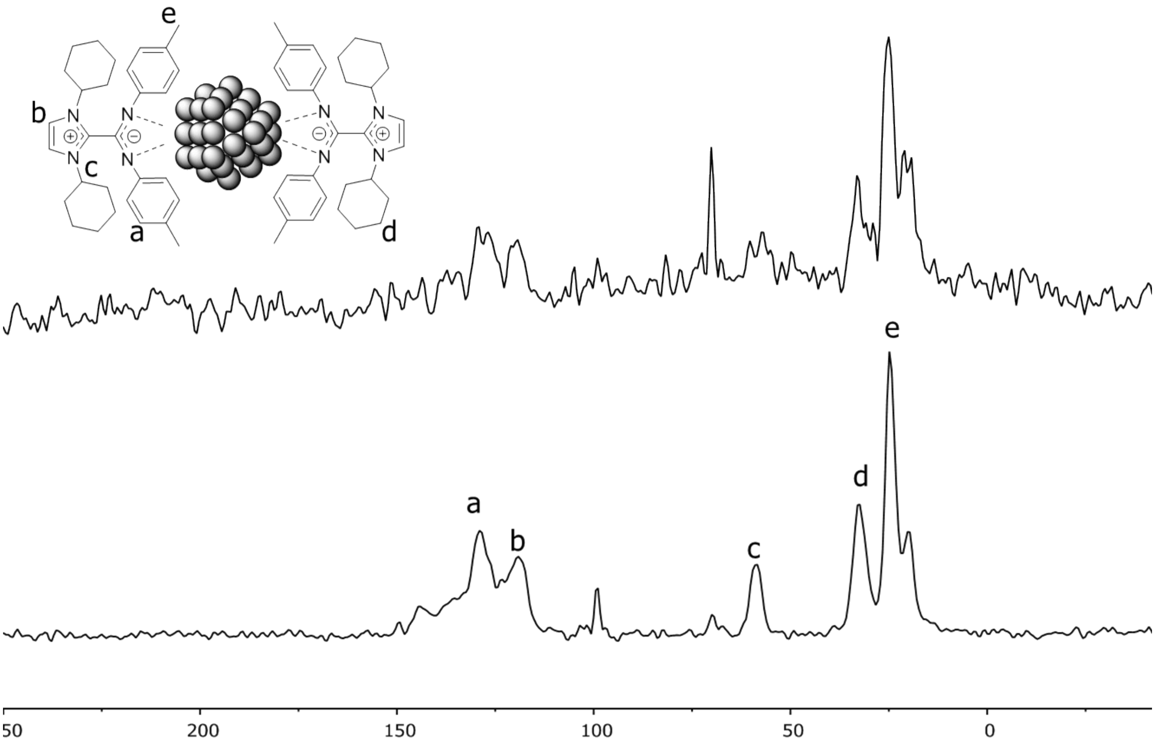

Figure S34. ^{13}C Hahn echo MAS (top) and CP-MAS (bottom) NMR spectra of Pt/CO/ICy$^{\cdot(p\text{-tol})}$NCN$_{0.2}$.

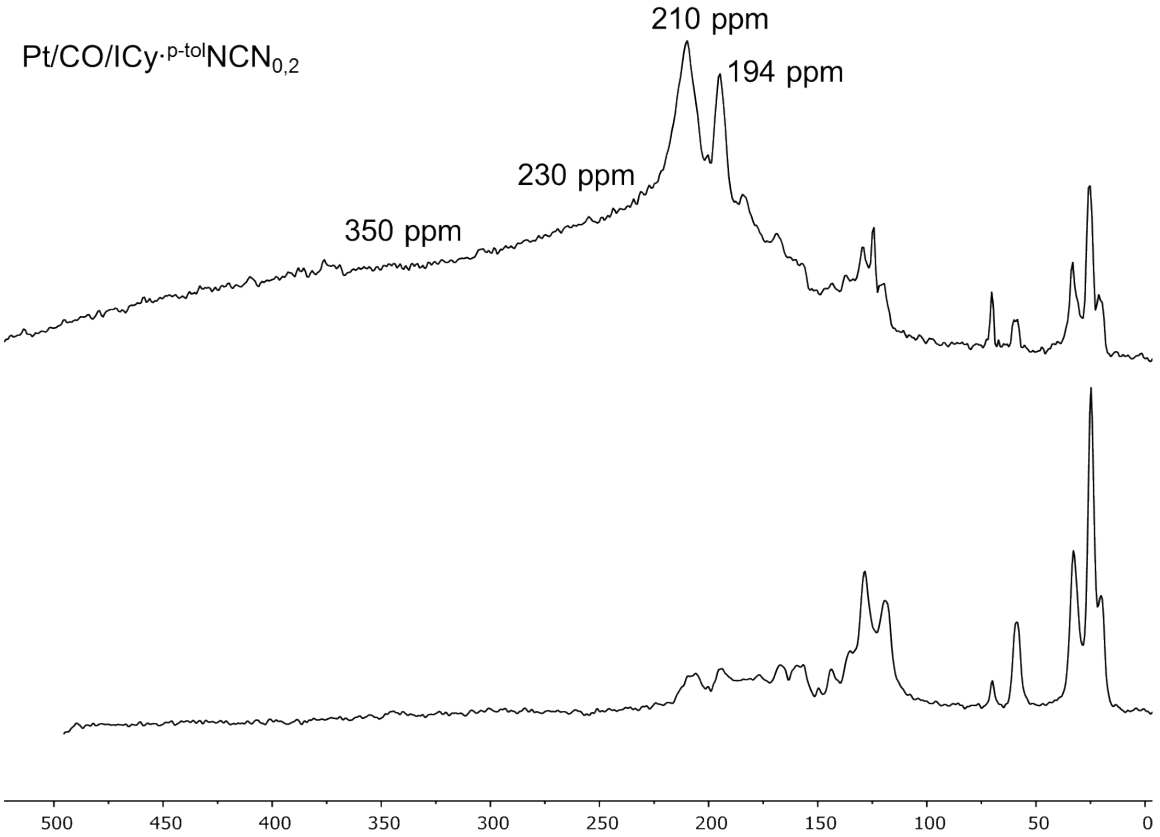

Figure S35. ^{13}C Hahn echo MAS (top) and CP-MAS (bottom) NMR spectra of Pt/CO/ICy$^{\cdot(p\text{-tol})}$NCN$_{0.2}$ after exposure to ^{13}CO (1 bar, 20 h, at r.t.).

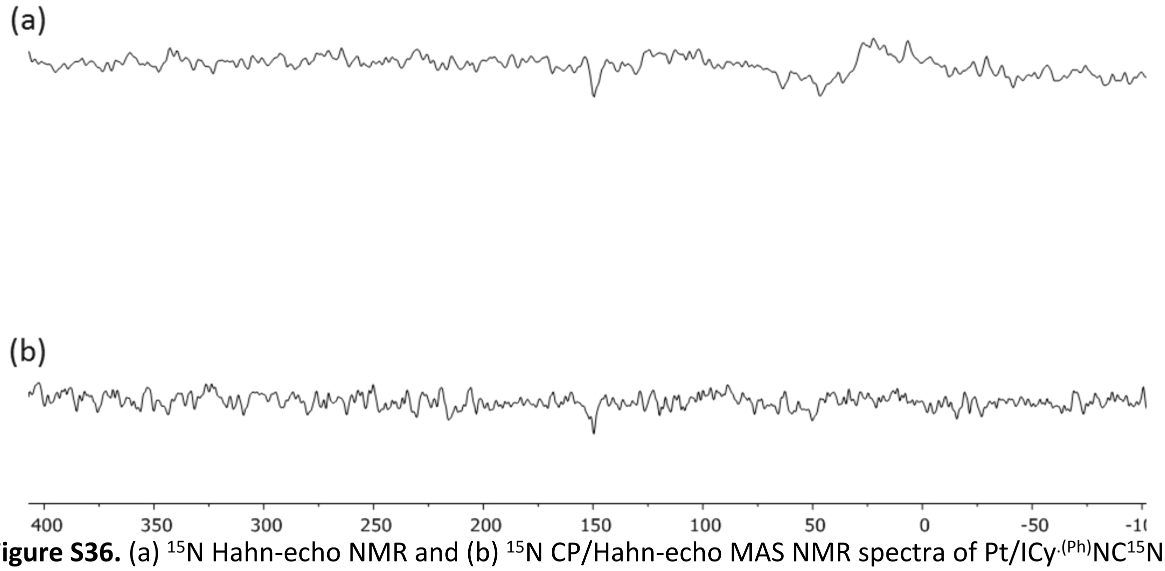

Figure S36. (a) ^{15}N Hahn-echo NMR and (b) ^{15}N CP/Hahn-echo MAS NMR spectra of Pt/ICy$^{\cdot(Ph)}$NC^{15}N$_{0.5}$.

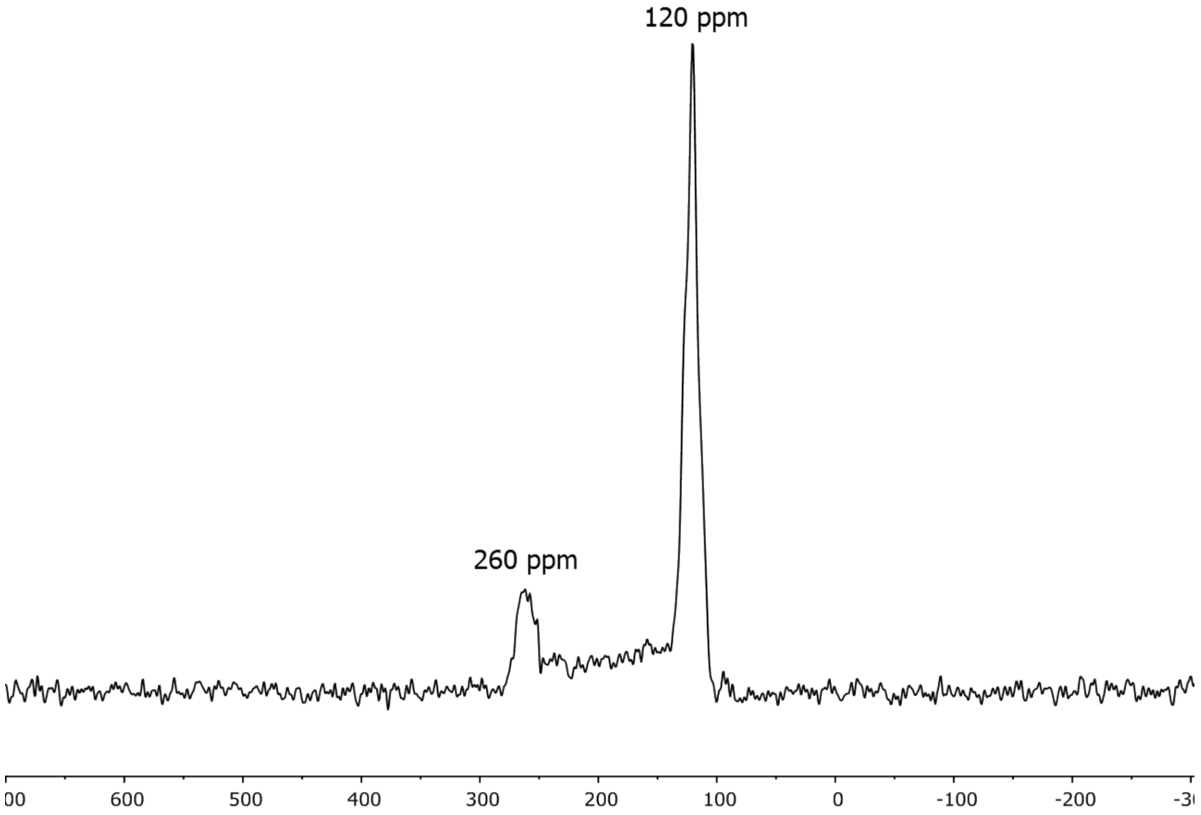

Figure S37. ^{15}N CP-Hahn echo MAS Pt/CO/ICy$^{\cdot(Ph)}$NC^{15}N$_{0.2}$.

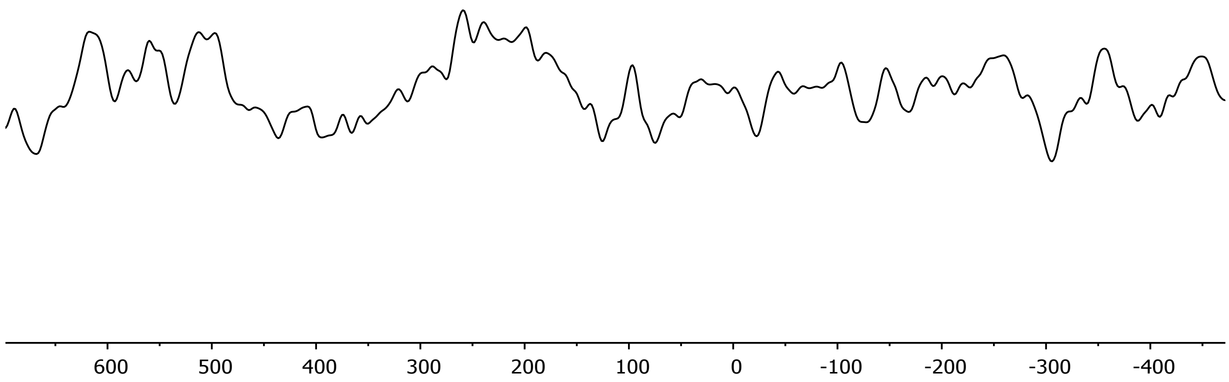

Figure S38. ^{15}N Hahn echo MAS Pt/CO/ICy$^{\cdot(Ph)}$NC^{15}N$_{0.2}$.

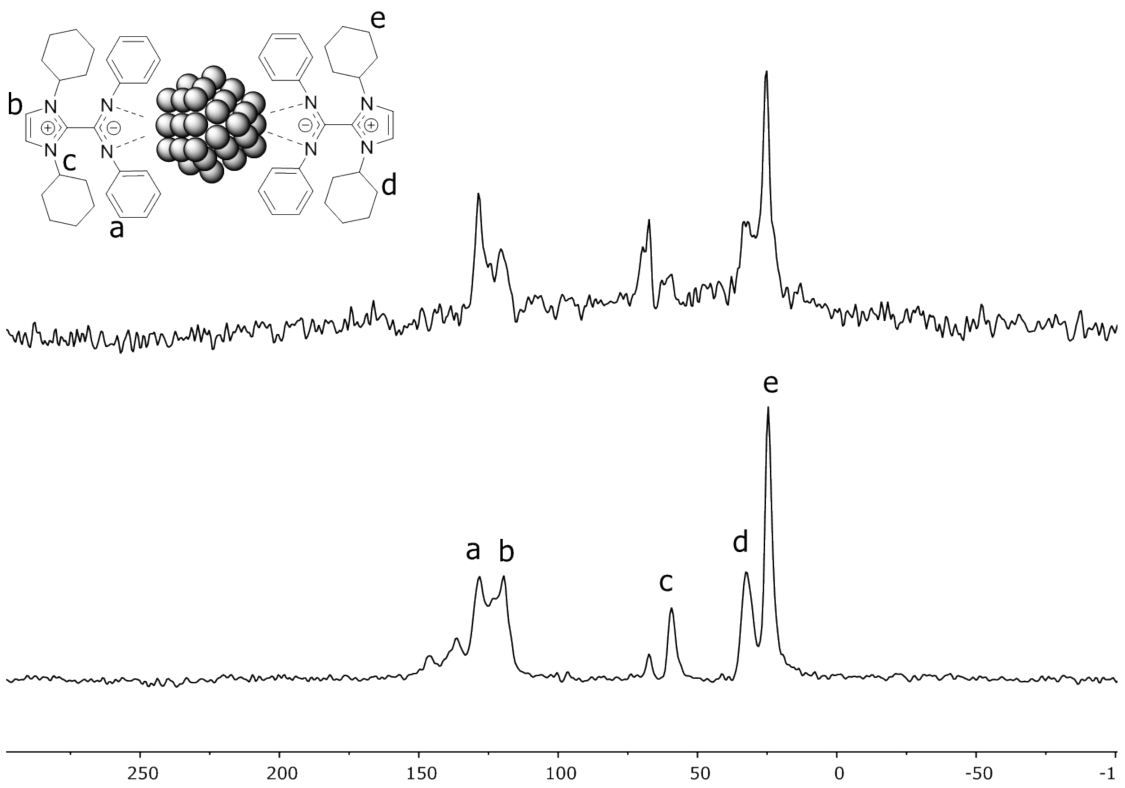

Figure S39. ^{13}C Hahn echo MAS (top) and CP-MAS (bottom) NMR spectra of Pt/CO/ICy·$^{(Ph)}$NC^{15}N$_{0.2}$.

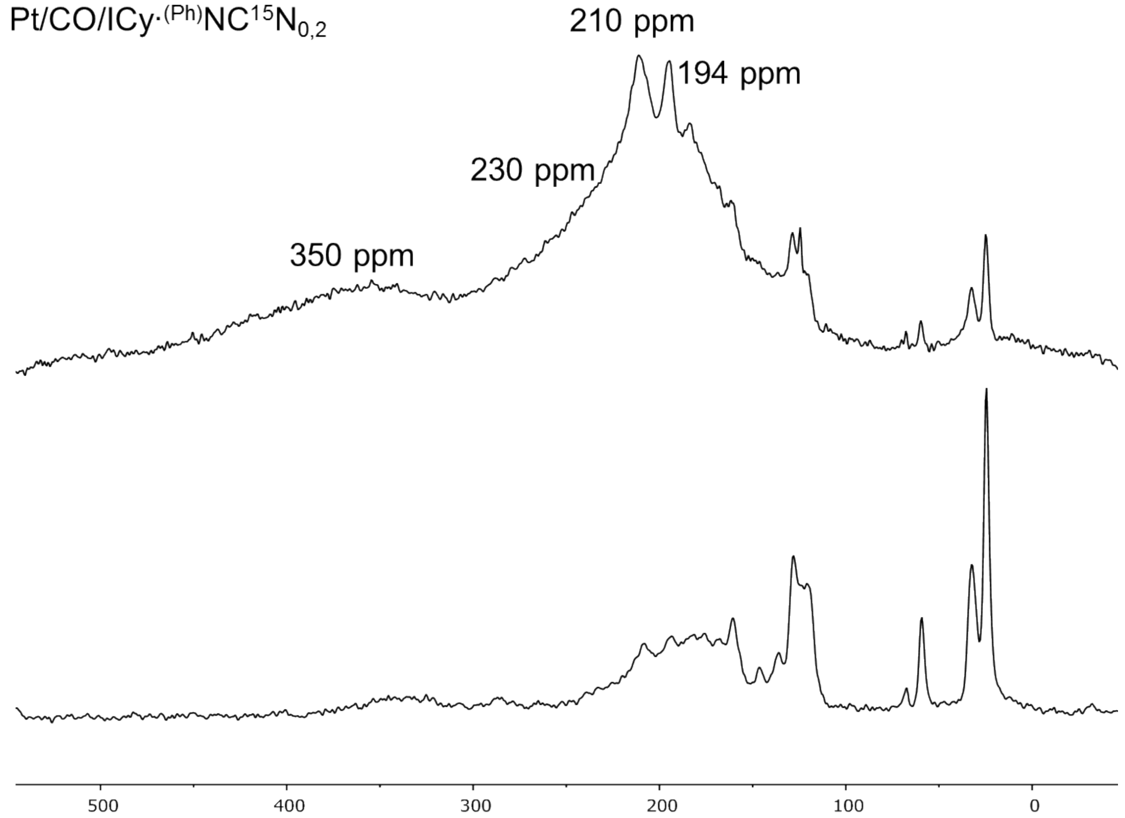

Figure S40. ^{13}C Hahn echo MAS (top) and CP-MAS (bottom) NMR spectra of Pt/CO/ICy·$^{(Ph)}$NC^{15}N$_{0.2}$ after exposure to ^{13}CO (1 bar, 20 h, at r.t.).

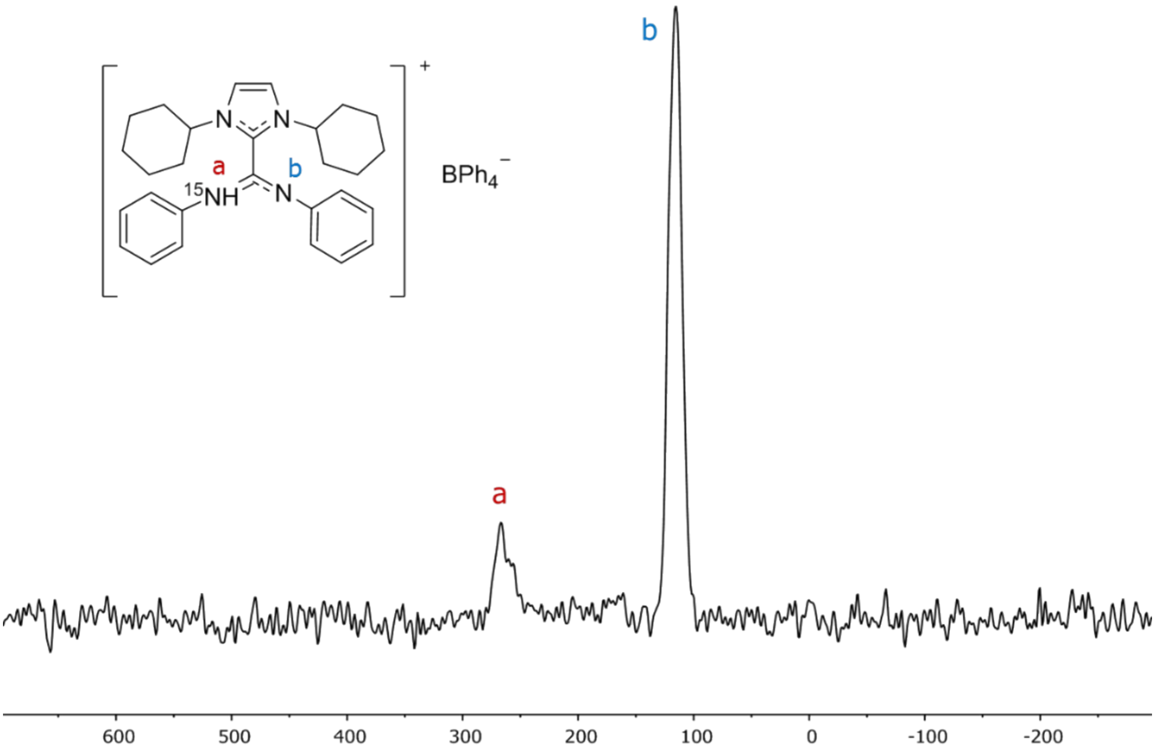

Figure S41. ^{15}N CP-Hahnecho MAS of [H·ICy·$^{(Ph)}$NC^{15}N]$^{+}$·[BPh4]

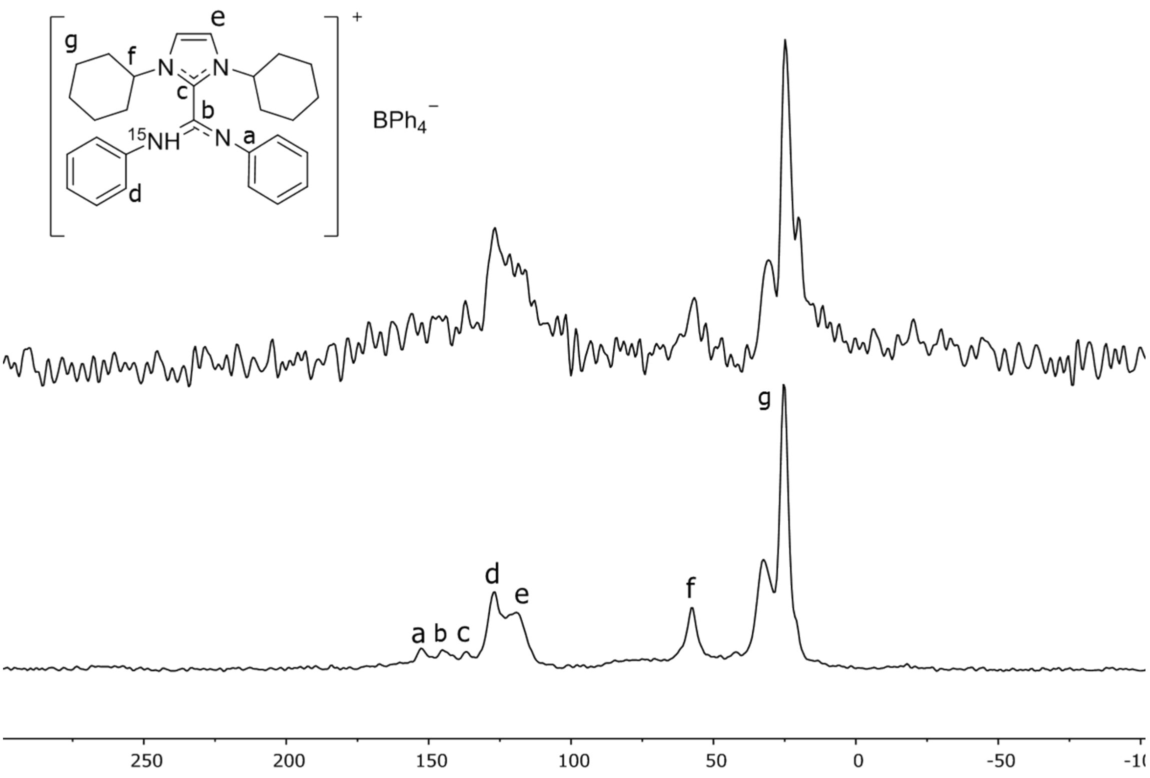

Figure S42. ^{13}C MAS (top) and CP-MAS (bottom) NMR spectra of [H·ICy·$^{(Ph)}$NC^{15}N]$^{+}$·[BPh4]$^{-}$.

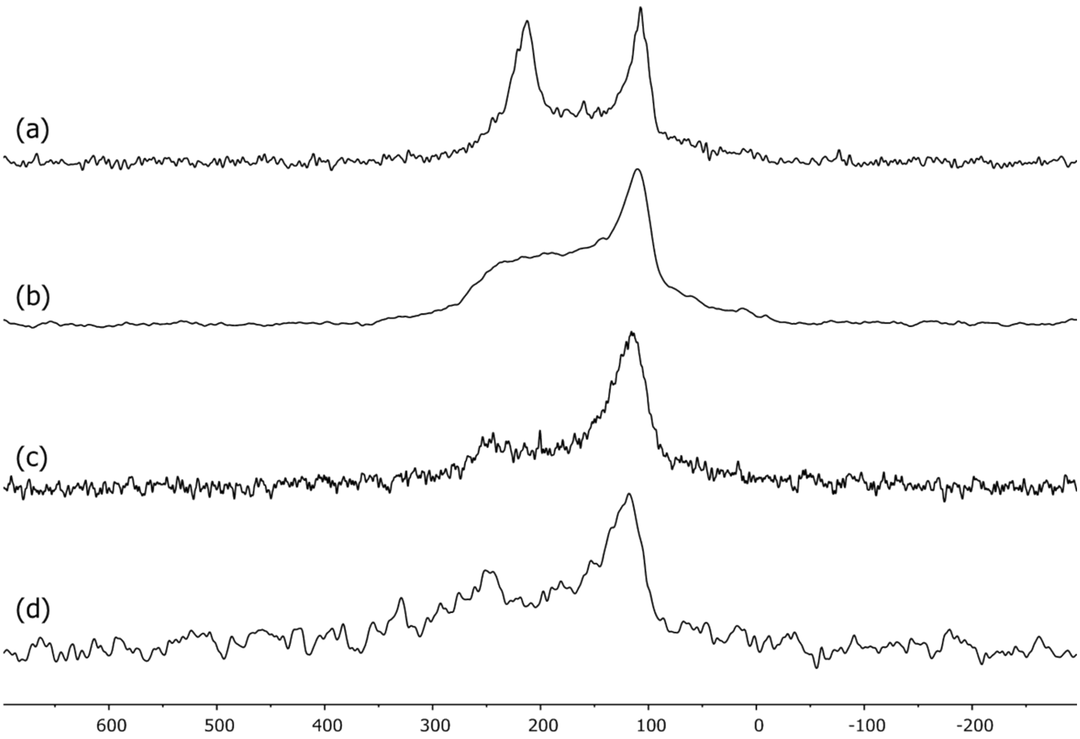

Figure S43. ^{15}N CP/Hahn-echo MAS NMR spectra of Ru/ICy$^{\cdot(Ph)}$NC^{15}N stabilized with (a) 1 equiv., (b) 0.5 equiv., (c) 0.2 equiv. and (d) 0.1 equiv.

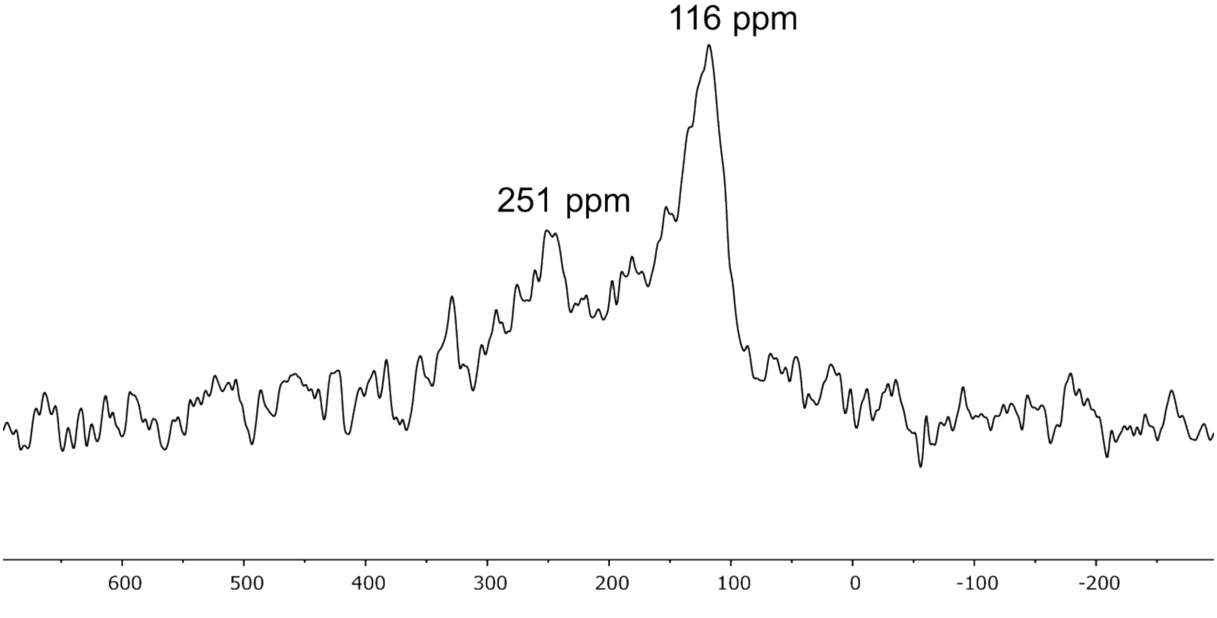

Figure S44. ^{15}N CP/Hahn-echo MAS NMR spectrum of Ru/ICy$^{\cdot(Ph)}$NC^{15}N$_{0.1}$.

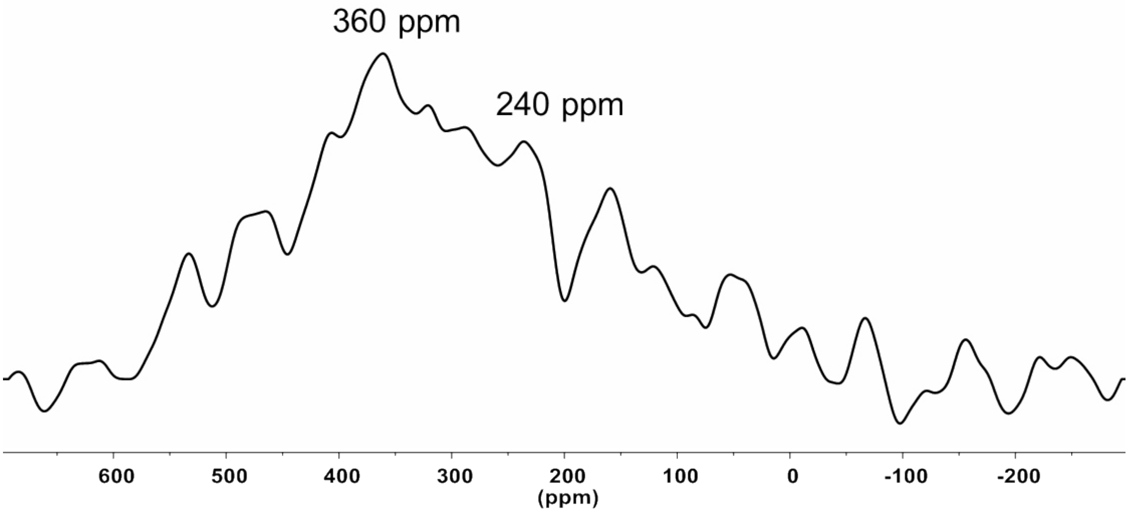

Figure S45. ^{15}N Hahn-echo MAS NMR spectrum of Ru/ICy$\cdot^{(Ph)}$NC$^{15}N_{0.1}$.

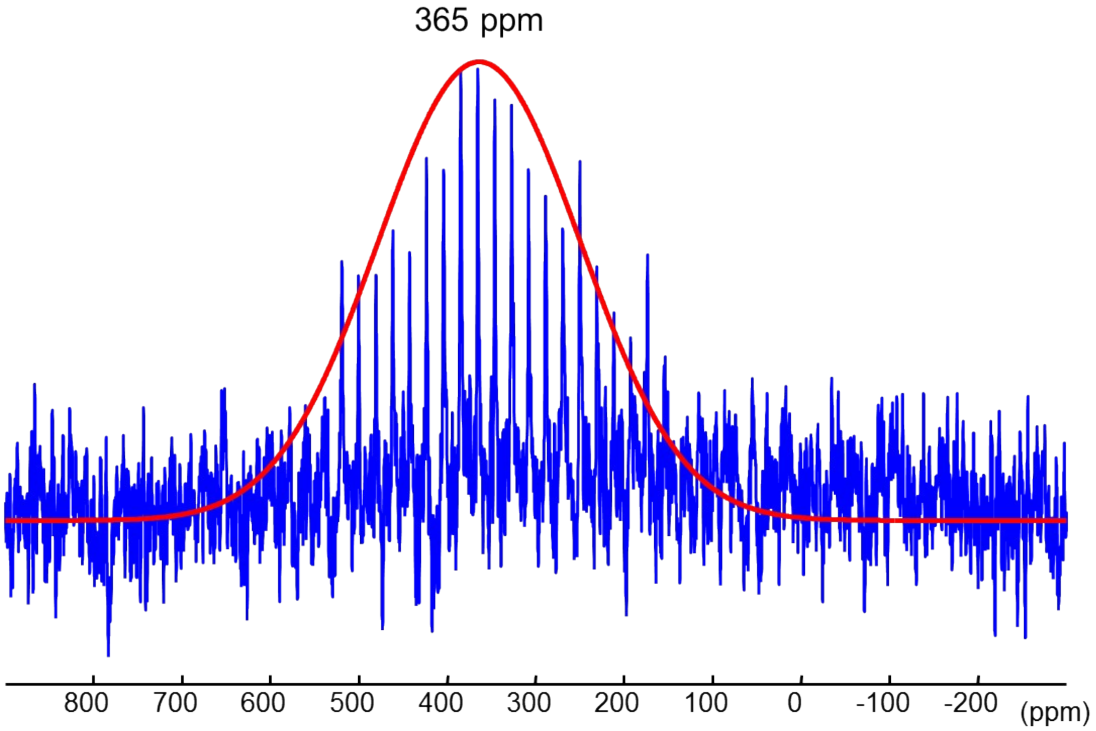

Figure S46. ^{15}N CPMG MAS NMR spectrum of Ru/ICy$\cdot^{(Ph)}$NC$^{15}N_{0.1}$.

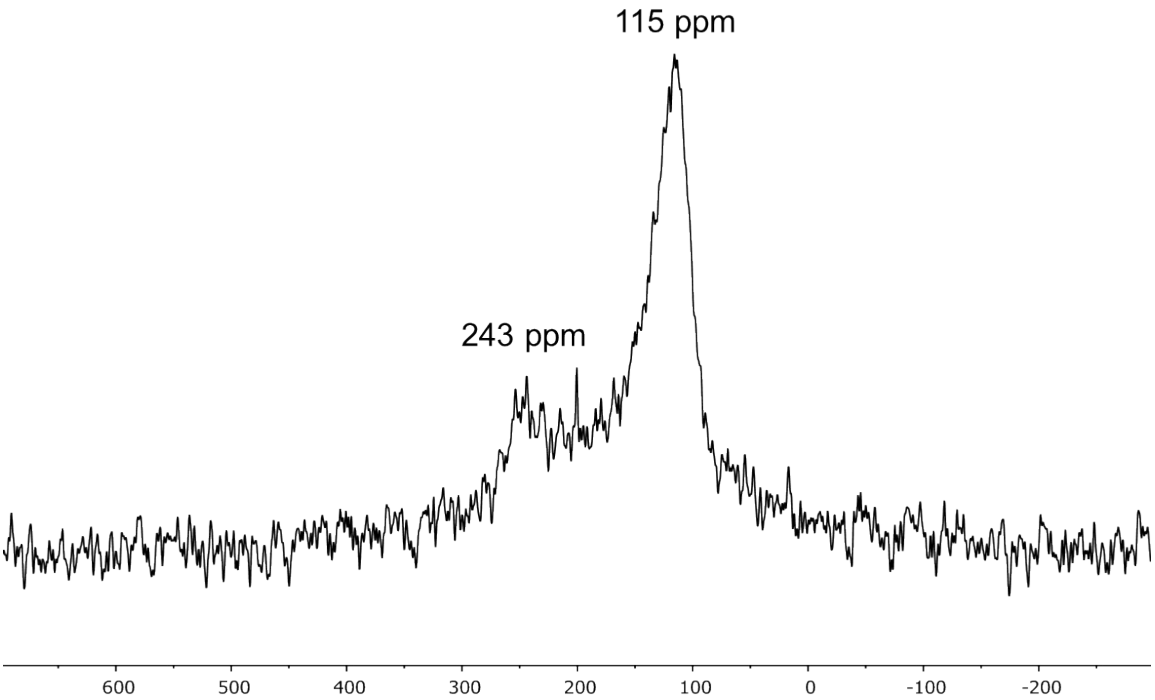

Figure S47. ^{15}N CP/Hahn-echo MAS NMR spectrum of Ru/ICy$^{\cdot(Ph)}$NC^{15}N$_{0.2}$.

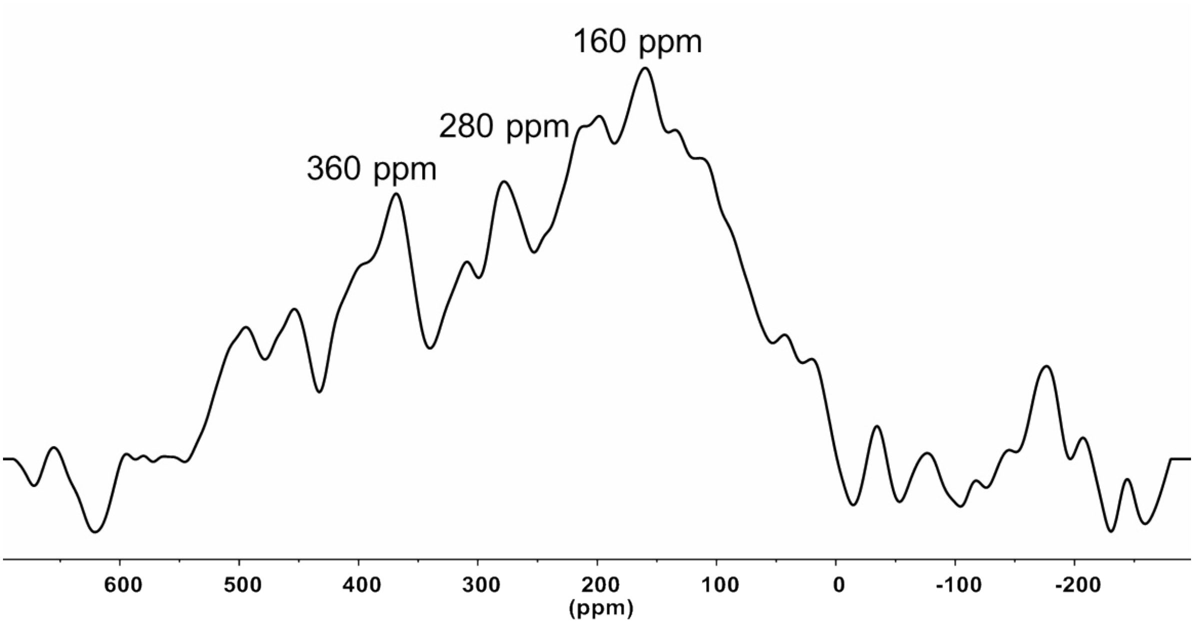

Figure S48. ^{15}N Hahn-echo MAS NMR spectrum of Ru/ICy$^{\cdot(Ph)}$NC^{15}N$_{0.2}$.

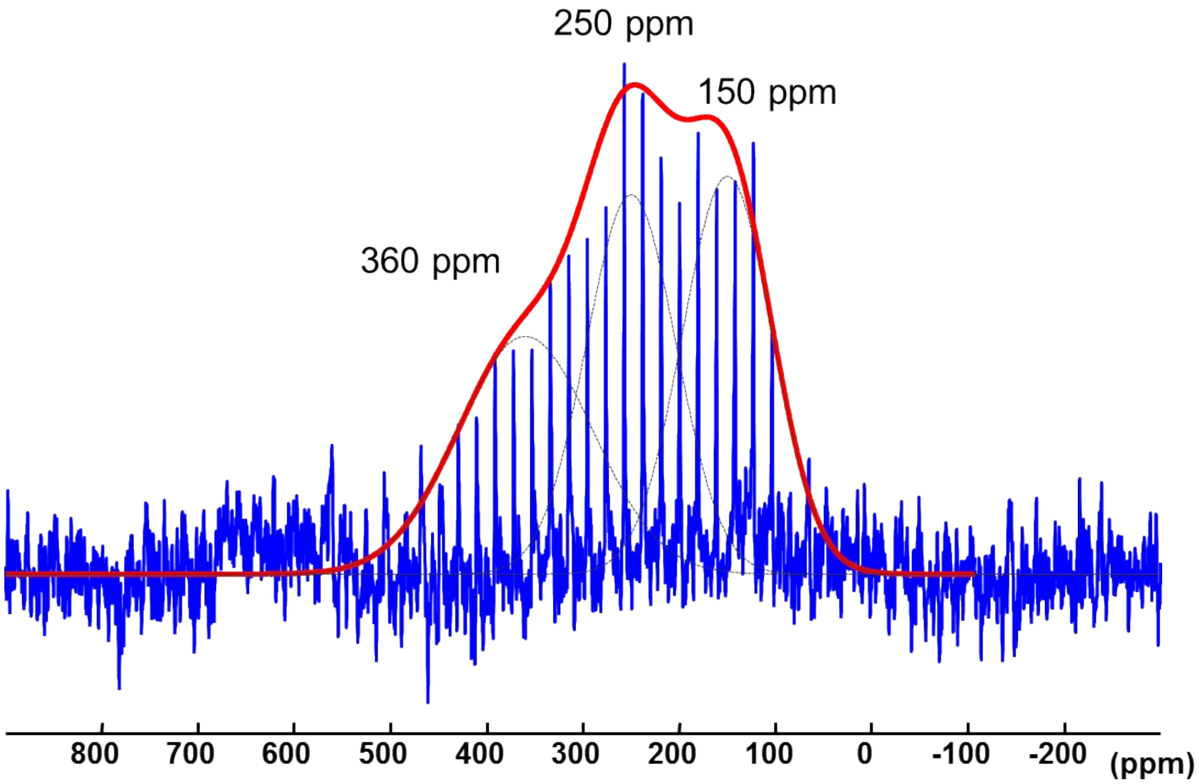

Figure S49. ^{15}N CPMG MAS NMR spectrum of Ru/ICy$^{\cdot(Ph)}$NC^{15}N$_{0.2}$.

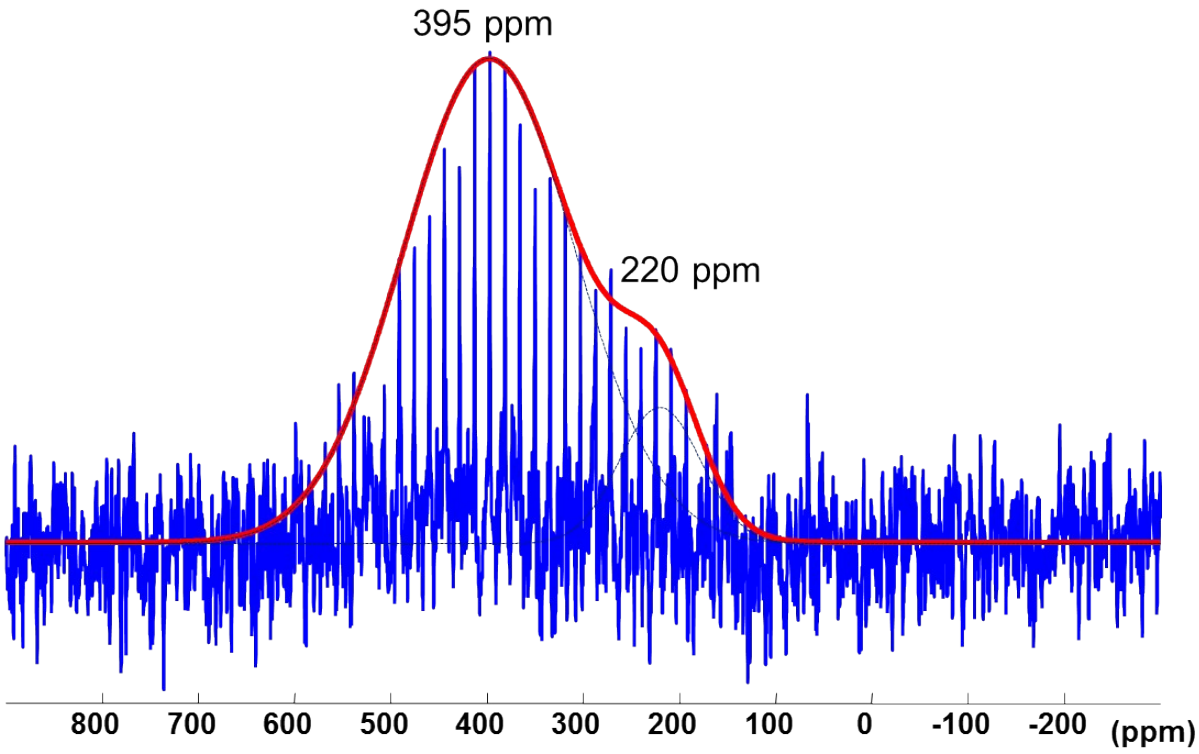

Figure S50. ^{15}N CPMG MAS NMR spectrum of Ru/ICy$^{\cdot(Ph)}$NC^{15}N$_{0.2}$ after exposure to ^{13}CO

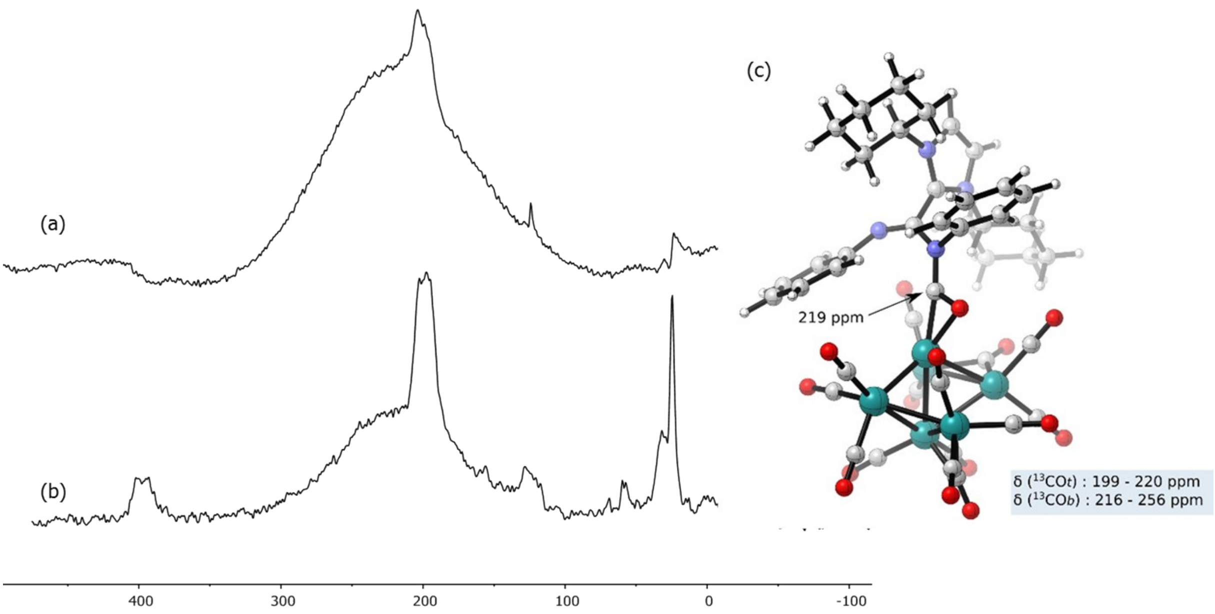

Figure S51. (a) ^{13}C Hahn-echo NMR MAS and (b) CP MAS spectra of Ru/ICy$^{\cdot(Ph)}$NC^{15}N$_{0.2}$ after exposure to ^{13}CO (1 bar, 20 h, at r.t.). (c) Calculated ^{13}C NMR displacements of ICy$^{\cdot(Ph)}$NCN after CO insertion on a [Ru$_6$] cluster model.

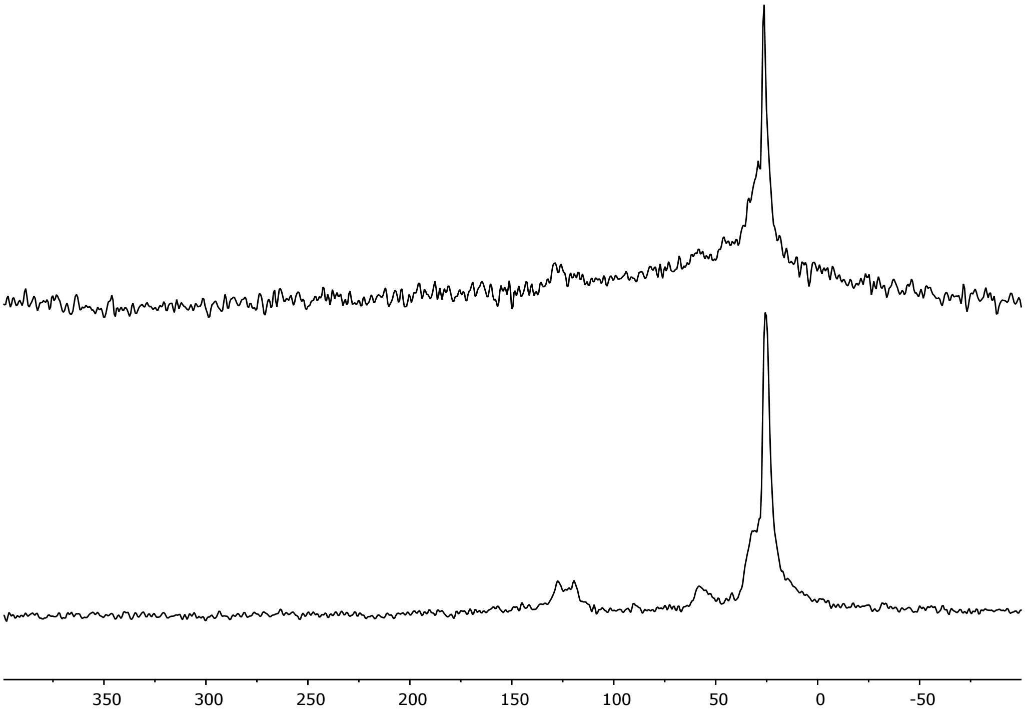

Figure S52.^{13}C Hahn-echo MAS (top) and CP MAS (bottom) NMR spectra of Ru/ICy$^{\cdot(Ph)}$NC^{15}N$_{0.2}$.

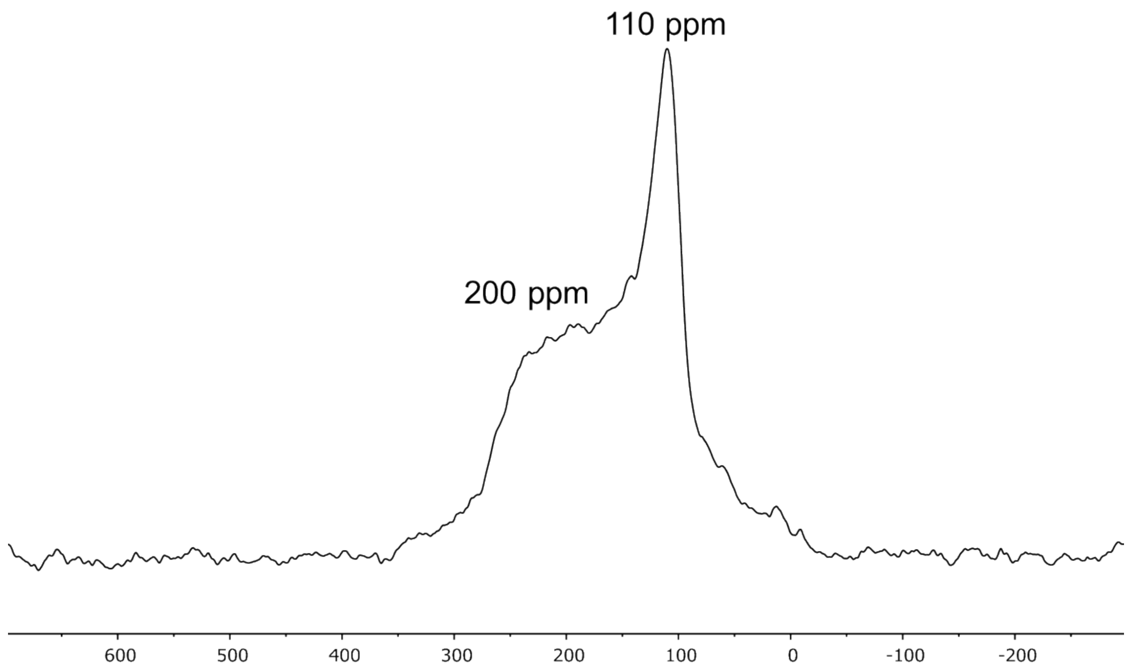

Figure S53. ^{15}N CP/Hahn-echo MAS NMR spectrum of Ru/ICy$^{\cdot(Ph)}$NC$^{15}N_{0.5}$.

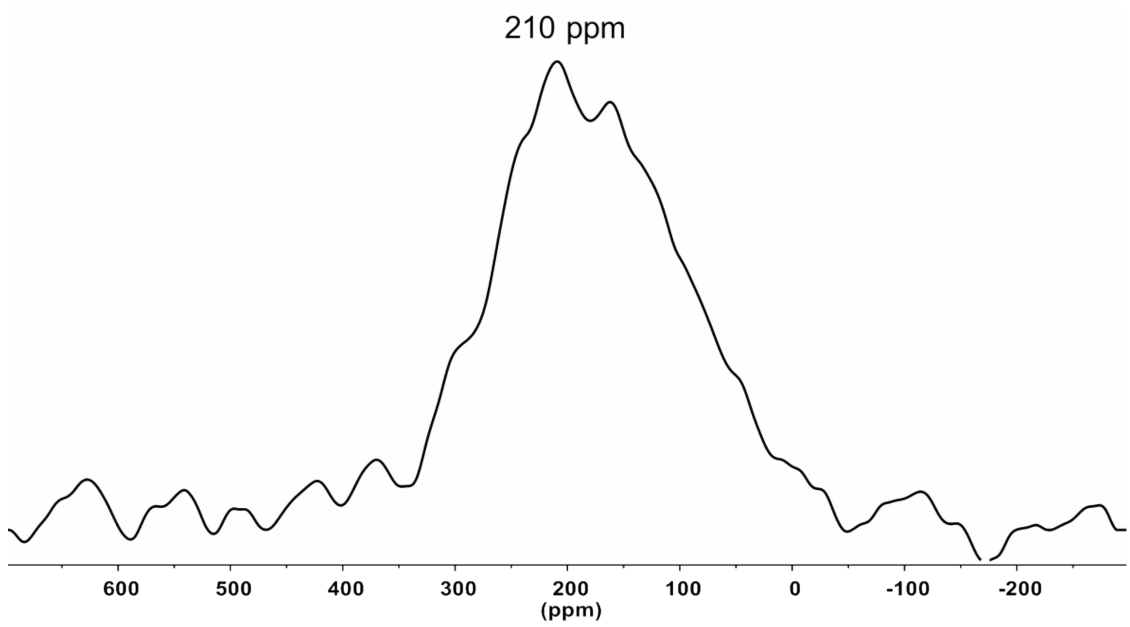

Figure S54. ^{15}N Hahn echo MAS (bottom) NMR spectrum of Ru/ICy$^{\cdot(Ph)}$NC$^{15}N_{0.5}$.

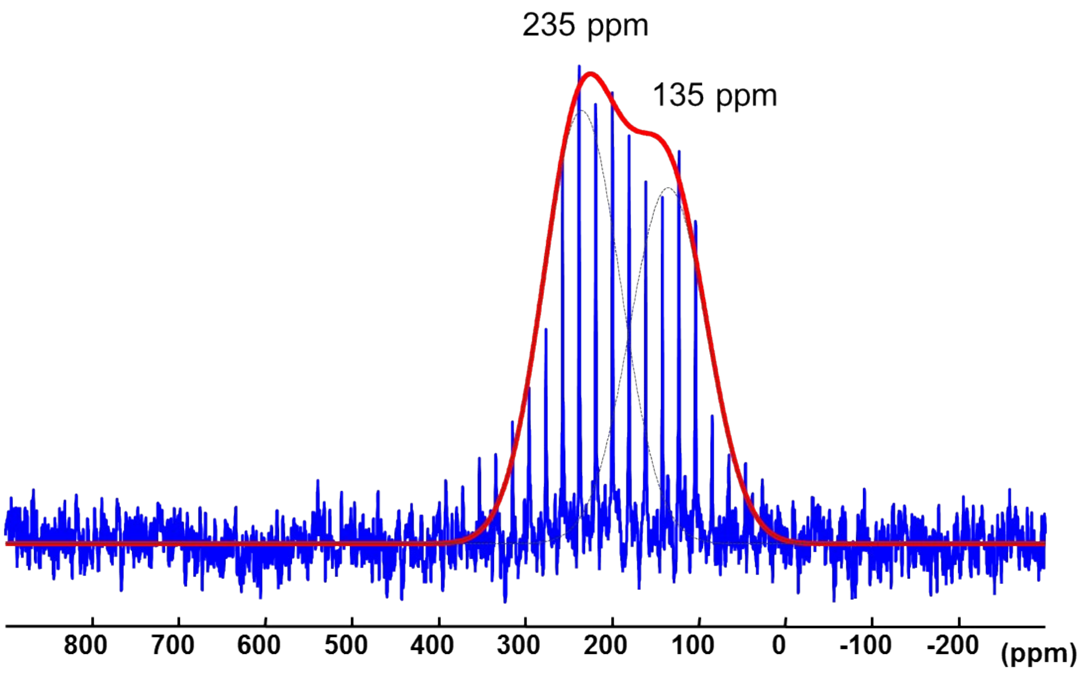

Figure S55. ^{15}N CPMG MAS NMR spectrum of Ru/ICy$^{\cdot(Ph)}$NC^{15}N$_{0.5}$.

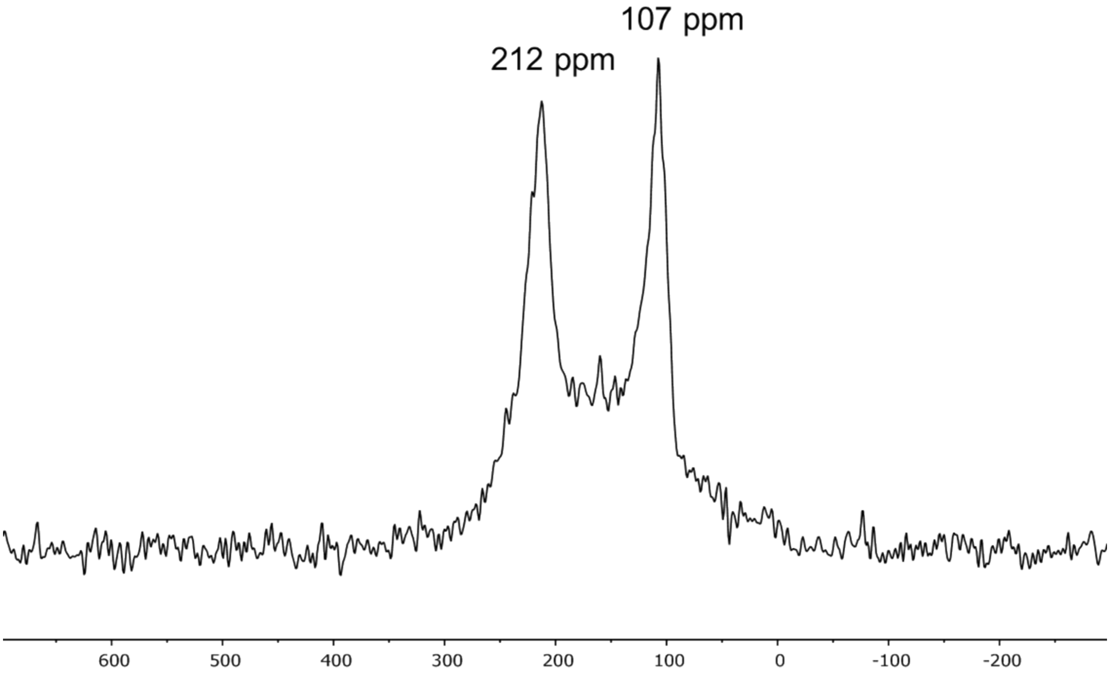

Figure S56. ^{15}N CP/Hahn-echo MAS NMR spectrum of Ru/ICy$^{\cdot(Ph)}$NC^{15}N$_1$.

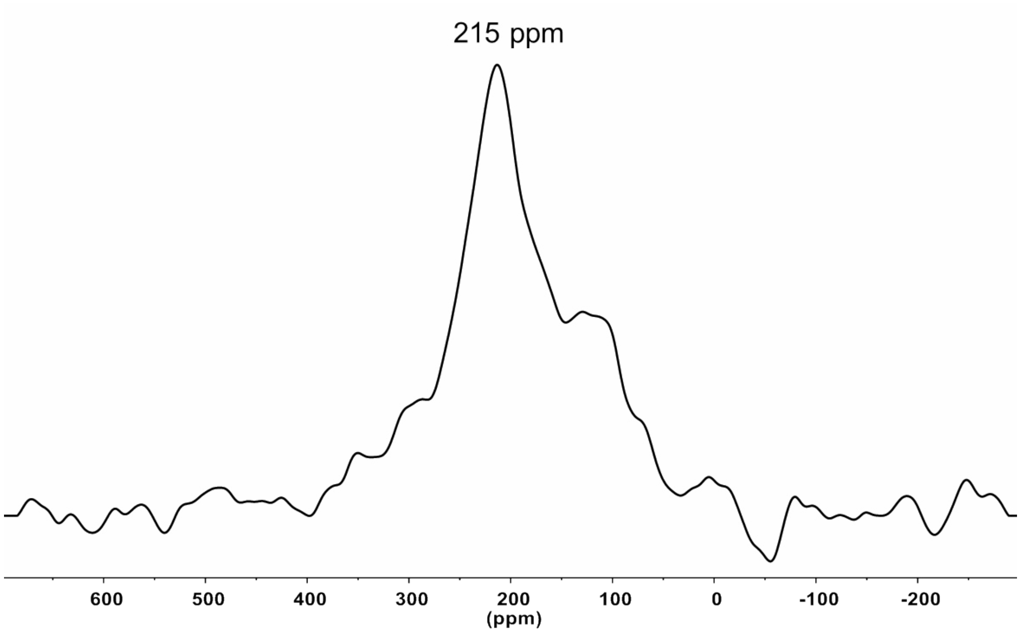

Figure S57. ^{15}N Hahn echo MAS (bottom) NMR spectrum of Ru/ICy$^{\cdot(Ph)}$NC^{15}N$_1$.

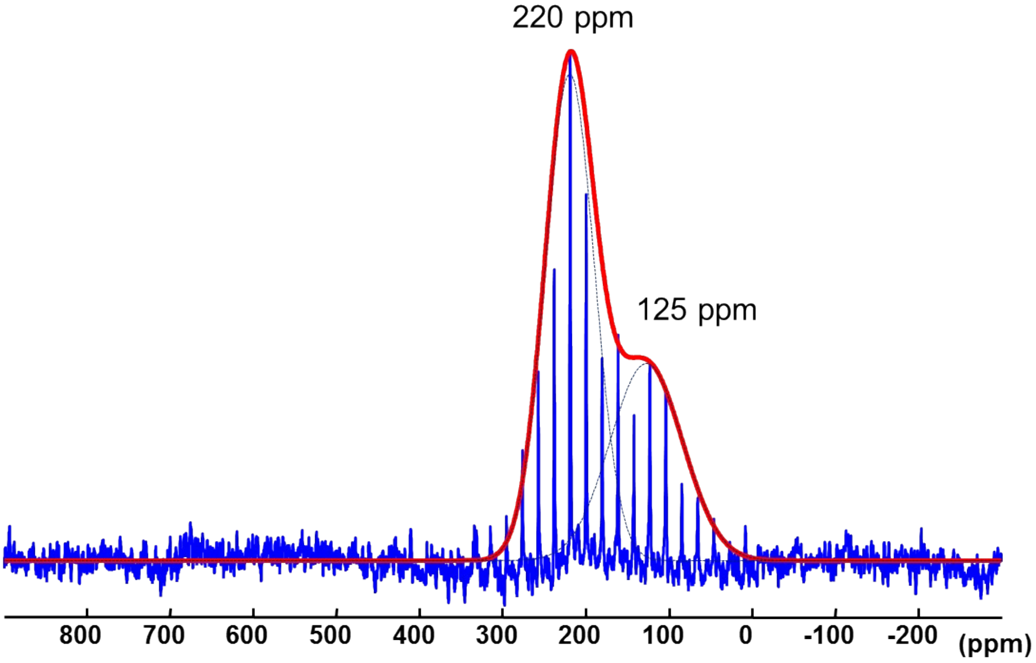

Figure S58. ^{15}N CPMG MAS NMR spectrum of Ru/ICy$^{\cdot(Ph)}$NC^{15}N$_1$.

DFT data

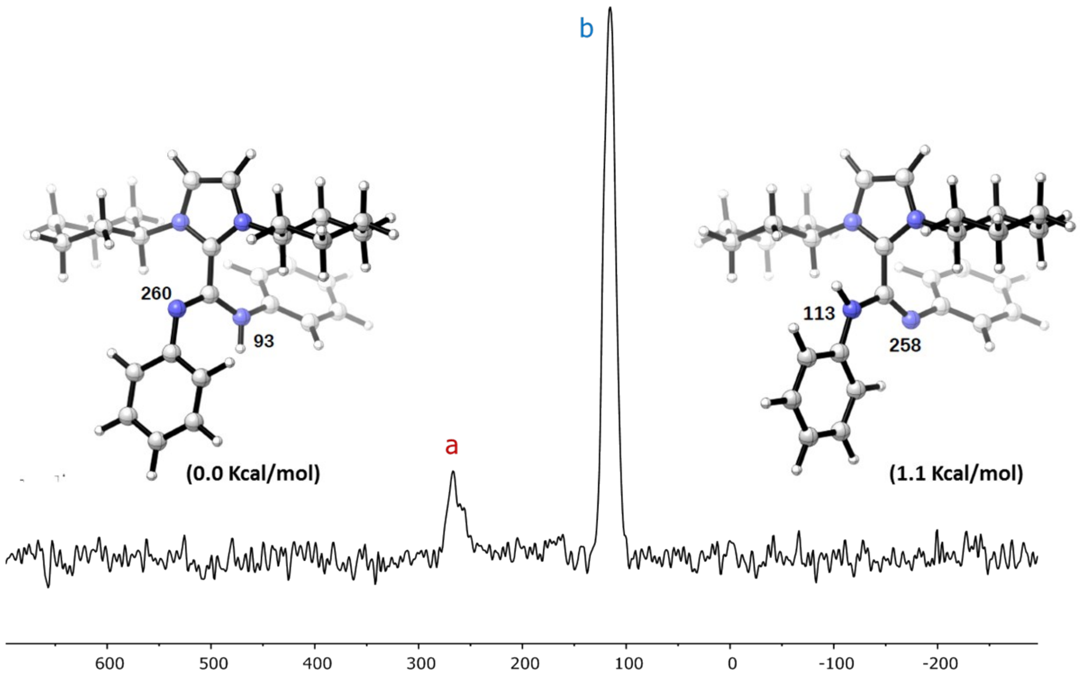

^{15}N signal	Observed (ppm)	Calculated (ppm)	Calculated (ppm)
a	266	260	258
b	115	93	113

Figure S59. Observed and calculated ^{15}N MAS NMR signals of [H·ICy·$^{(Ph)}$NC^{15}N]$^{+}$·[BPh4].

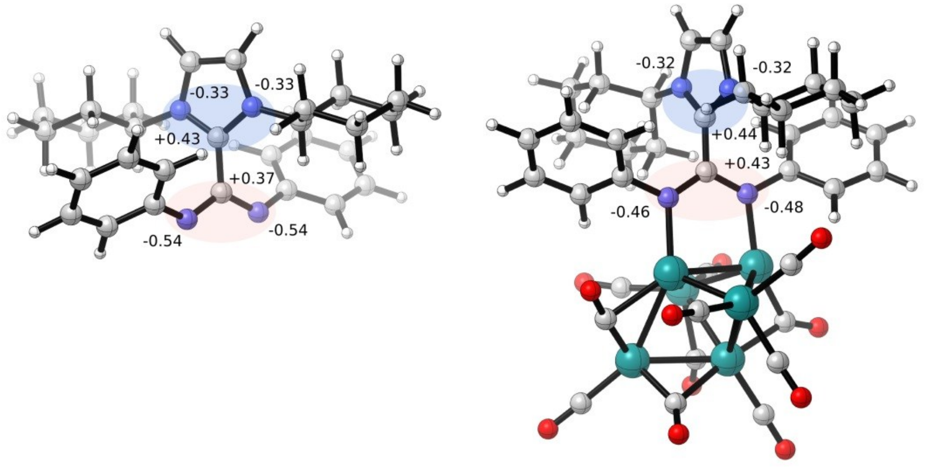

Figure S60. NPA charges[9], obtained with Gaussian 09 at the B3PW91 level of theory, of the N atoms of free and μ$_2$-κ ^{1}N, κ^{1}N´ ICy·$^{(Ph)}$NCN coordinated ligands.

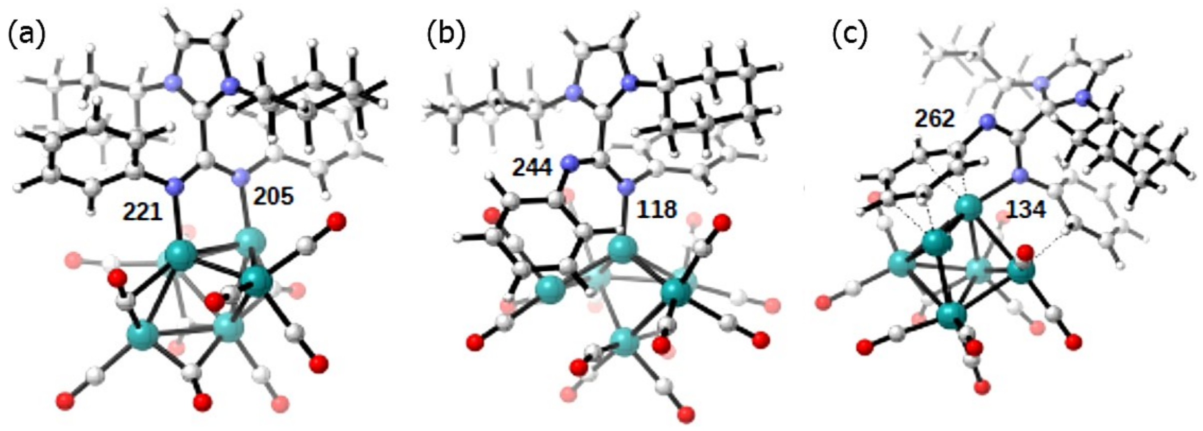

Figure S61. Estimated ^{15}N NMR displacements of ICy·$^{(Ph)}$NCN coordinated to a model Ru carbonyl cluster through: (a) two N atoms, (b) one N atom and (c) one N atom and π-stacking.

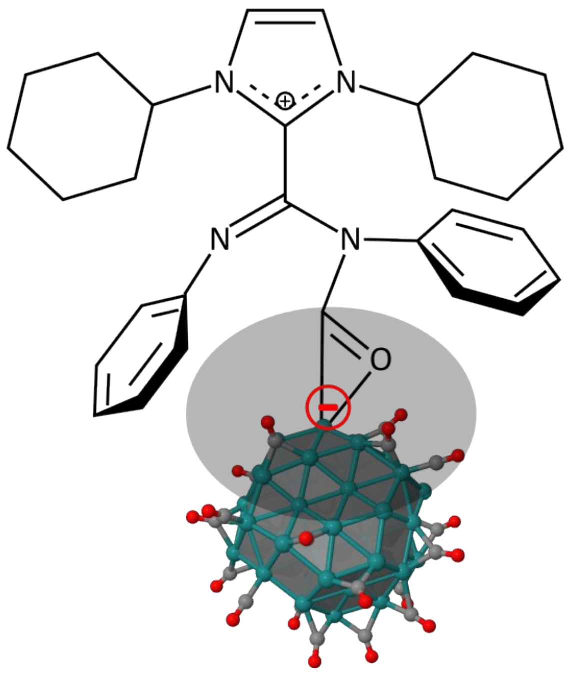

Figure S62. Reorganization of the bonding scheme due to CO insertion. It is supported by bond lengths comparison and by charge analysis (NPA on the cluster model and Mulliken-like analysis on nanoparticles, see **Figures S63 and S64**).

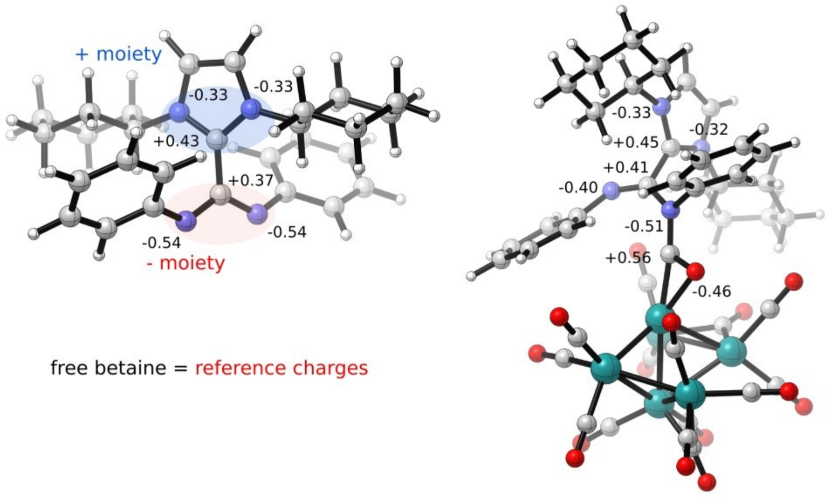

Figure S63. NPA charges[9] obtained with Gaussian09, at the DFT-B3PW91 level of theory.

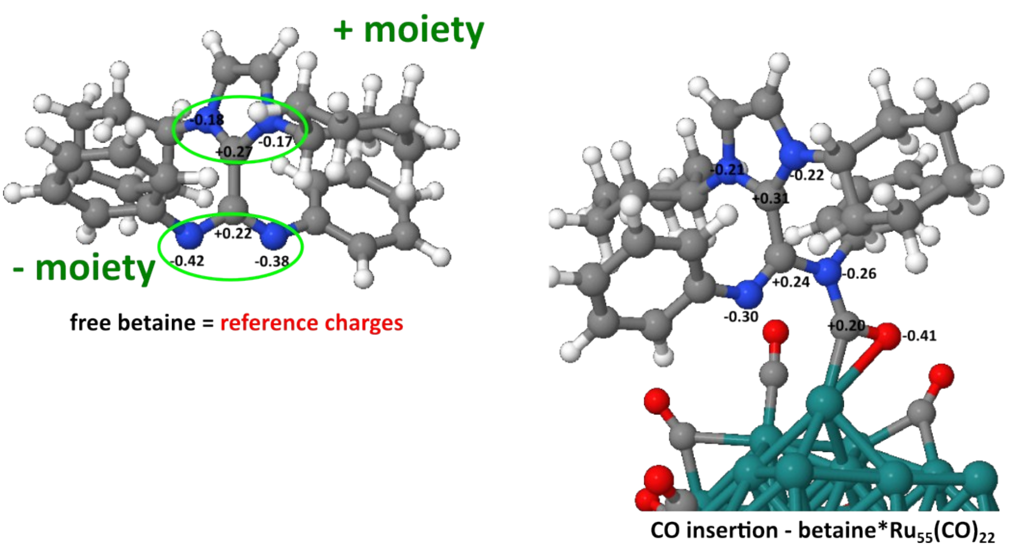

Figure S64. Mulliken-like analysis obtained after integration of the pDOS calculated with the Lobster package.[15] It has been preferred to Bader analysis, which was unable to converge on the zwitterionic feature of ICy·(Ph)NCN.

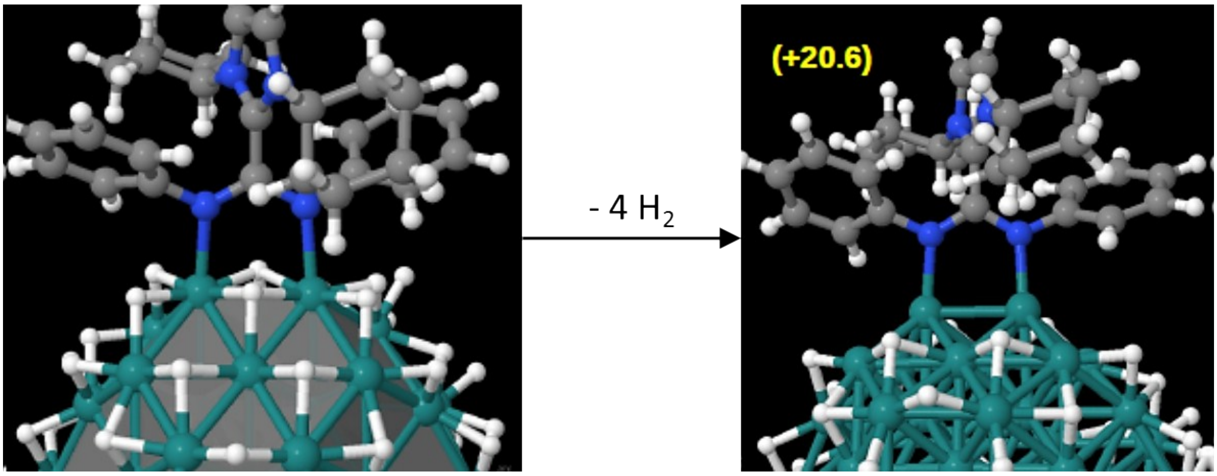

Figure S65. Adsorption energy calculated (Kcal/mol) of ICy$^{\cdot(Ph)}$NCN at the Ru_{55} surface after the lost of 4 H_2.

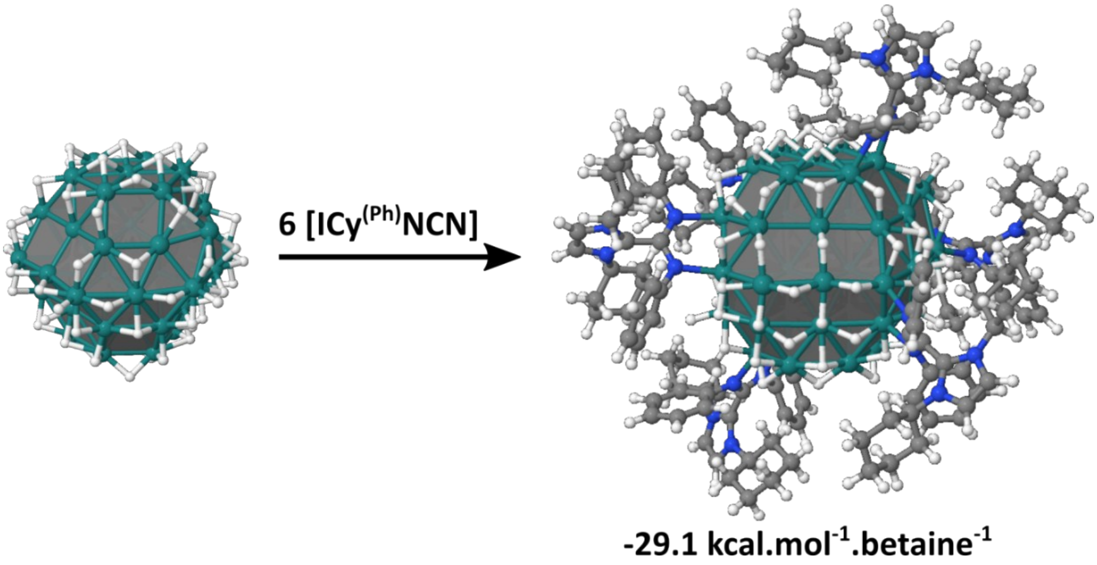

Figure S66. $Ru_{55}H_{70}$ with six molecules of ICy$^{\cdot(Ph)}$NCN.

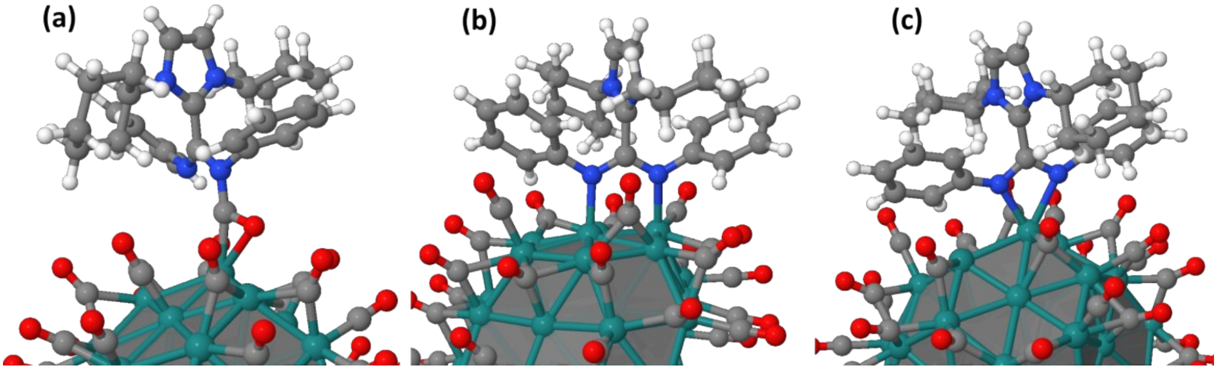

Figure S67. Adsorption modes of ICy$^{\cdot(Ph)}$NCN at the $Ru_{55}CO_{59}$ surface . (a) after CO insertion; CO^{ins} compound (-27.3 kcal.mol^{-1}); (b) bicoordinated mode on a Ru(001) facet, μ_2-κ^1N, κ^1N′ (-47.1 kcal.mol^{-1}); (c) bicoordinated κ^2N, N′ (-76.0 kcal.mol^{-1})

XPS data

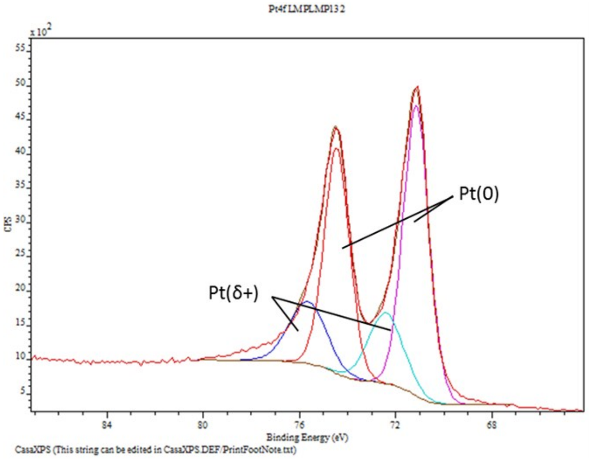

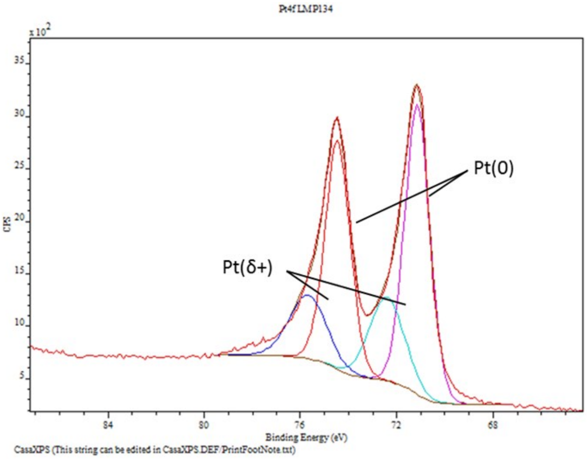

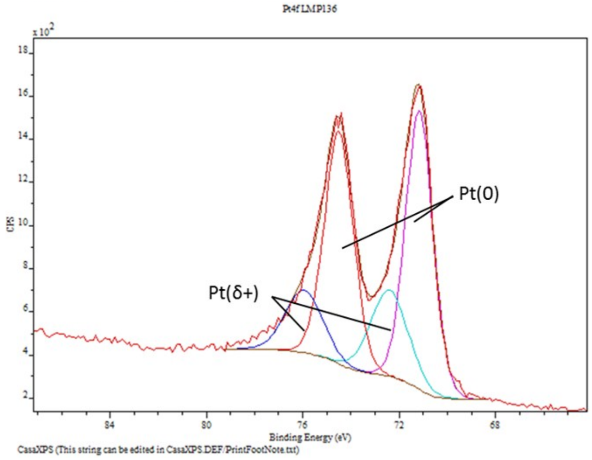

	pTol	PhCl	PhOMe
Ptcore/Pt	0.74	0.71	0.72

Figure S68. X-ray photoelectron spectroscopy (XPS) of the Pt(4f) signals of (a) Pt/ICy$^{\cdot(p\text{-tol})}$NCN$_{0.1}$, (b) Pt/ICy$^{\cdot(p\text{-tol})}$NCN$_{0.2}$ and (c) Pt/ICy$^{\cdot(p\text{-tol})}$NCN$_{0.5}$.
